# Agent‐based modelling reveals strategies to reduce the fitness and metastatic potential of circulating tumour cell clusters

**DOI:** 10.1111/eva.12943

**Published:** 2020-03-18

**Authors:** Marco Campenni, Alexander N. May, Amy Boddy, Valerie Harris, Aurora M. Nedelcu

**Affiliations:** ^1^ Biosciences University of Exeter Penryn UK; ^2^ Department of Psychology Arizona State University Tempe AZ USA; ^3^ Research Casting International Quinte West ON Canada; ^4^ Biodesign Institute Arizona State University Tempe AZ USA; ^5^ Department of Anthropology University of California Santa Barbara Santa Barbara CA USA; ^6^ Biology Department University of New Brunswick Fredericton NB Canada

**Keywords:** agent‐based model, anoikis, cancer, circulating tumour cell clusters, metastasis

## Abstract

Metastasis—the ability of cancer cells to disperse throughout the body and establish new tumours at distant locations—is responsible for most cancer‐related deaths. Although both single and clusters of circulating tumour cells (CTCs) have been isolated from cancer patients, CTC clusters are generally associated with higher metastatic potential and worse prognosis. From an evolutionary perspective, being part of a cluster can provide cells with several benefits both in terms of survival (e.g. protection) and reproduction (group dispersal). Thus, strategies aimed at inducing cluster dissociation could decrease the metastatic potential of CTCs. However, finding agents or conditions that induce the dissociation of CTC clusters is hampered by the fact that their detection, isolation and propagation remain challenging. Here, we used a mechanistic agent‐based model to (a) investigate the response of CTC clusters of various sizes and densities to different challenges—in terms of cell survival and cluster stability, and (b) make predictions as to the combination of factors and parameter values that could decrease the fitness and metastatic potential of CTC clusters. Our model shows that the resilience and stability of CTC clusters are dependent on both their size and density. Also, CTC clusters of distinct sizes and densities respond differently to changes in resource availability, with high‐density clusters being least affected. In terms of responses to microenvironmental threats (such as drugs), increasing their intensity is, generally, least effective on high‐density clusters. Lastly, we found that combining various levels of resource availability and threat intensity can be more effective at decreasing the survival of CTC clusters than each factor alone. We suggest that the complex effects that cluster density and size showed on both the resilience and stability of the CTC clusters are likely to have significant consequences for their metastatic potential and responses to therapies.

## 1 INTRODUCTION

Metastasis—the ability of cancer cells to disperse throughout the body and establish new tumours at distant locations—is responsible for the majority of cancer‐related deaths (Chaffer & Weinberg, 2011). The initiation of secondary tumours was originally believed to involve the dispersal of individual cancer cells, and consistent with this view, single circulating tumour cells (CTCs) have been isolated from the blood of cancer patients as early as mid‐19th century (Ashworth, 1869). More recently, however, groups of CTCs have also been found in blood samples (Aceto, Toner, Maheswaran, & Haber, 2015; Massagué & Obenauf, 2016; Meunier et al., 2019). CTC clusters have been shown to detach from tumours as groups of cells and are known to increase during metastasis (Aceto et al., 2014; Cheung et al., 2016; Fabisiewicz & Grzybowska, 2017). Despite their lower frequency relative to single CTCs, CTC clusters exhibit higher metastatic potential and are associated with worse clinical outcome (Aceto et al., 2014; Cheung et al., 2016; Suo et al., 2017; Wang et al., 2017; Zhang et al., 2017).

**Figure 1:**
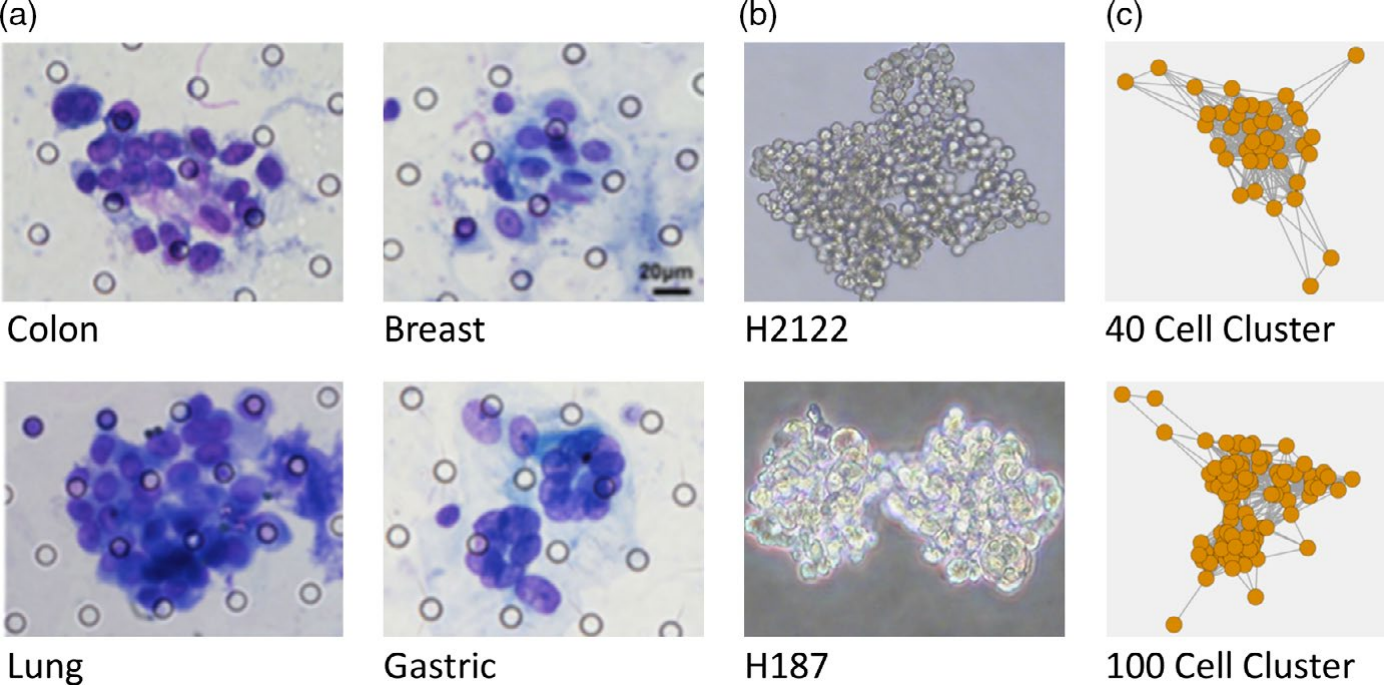
Examples of (a) CTC clusters isolated from patients with different types of cancer, showing distinct morphologies (shape and density) and cell numbers (adapted from Chen et al., 2017); (b) clusters from two lung cancer cell lines—a non‐small‐cell (H2122) and a small cell (H187), showing differences in cluster density; and (c) virtual clusters in our model at the start of the simulation

CTC clusters isolated from cancer patients vary in size between 2 and 100 cells (though most clusters range between 20 and 40 cells), with larger clusters being most aggressive (Liotta, Saidel, & Kleinerman, 1976; Rostami et al., 2019; Wang et al., 2017). The morphology and composition of CTC clusters also varies broadly. Clusters can be spherical, triangular or linear (Manjunath et al., 2019), and can form tightly packed, loosely connected or branched structures (Balakrishnan et al., 2019) (Figure 1a). Many clusters have been found to contain noncancer cells (e.g. fibroblasts, platelets) and are referred to as circulating tumour microemboli (Aceto et al., 2015). CTC clusters have a short life in circulation (minutes to hours; Aceto et al., 2014). Although some CTC clusters can assume linear configurations in transit (Au et al., 2016), they are generally assumed to become lodged in narrow capillaries, where they can remain dormant for extended periods of time, until they resume cell proliferation and establish secondary tumours (Giuliano et al., 2018). Nevertheless, some CTC clusters can also extravasate as multicellular clusters and have increased proliferative ability relative to single CTCs (Allen et al., 2019).

From an evolutionary and ecological perspective, being part of a cluster can provide cells with several benefits in terms of both survival (e.g. protection) and reproduction (group dispersal). Indeed, the dispersal of CTCs as clusters instead of single cells is thought to increase the likelihood of successful metastasis in several ways (Giuliano et al., 2018). For instance, cells in CTC clusters maintain strong cell–cell connections (including desmosomes and adherens junctions), the presence of which might confer resistance to anoikis (Hou et al., 2012; Yu et al., 2013)—a special form of apoptosis generally induced in single cells that detach from the extracellular matrix. Also, contrary to single CTCs, cells in CTC clusters have been found to lack proliferation markers and express more mesenchymal markers, which might explain their relatively higher survival and resistance to chemotherapy (Hou et al., 2012; Krebs et al., 2012). Cellular plasticity and cooperativity among cells in a cluster is also thought to confer resistance to stress during circulation (Micalizzi, Haber, & Maheswaran, 2017), protection against immune predation and more successful colonization at secondary sites (Hong, Li, & Zhang, 2016).

Despite the accepted view that CTC clusters are extremely relevant to the metastatic process and that their presence correlates with poor clinical outcome, we know little about their biology and possible means to decrease their metastatic potential (Giuliano et al., 2018). Based on the proposed and experimentally demonstrated increased metastatic potential of CTC clusters (relative to single CTCs), it has been suggested that their dissociation into single cells might be a valid therapeutic strategy to decrease their aggressivity (Aceto et al., 2014; Hong, Fang, & Zhang, 2016). Studies that directly targeted cell–cell connections and adhesion within clusters by downregulating the expression of plakoglobin (a protein involved in desmosomes and adherens junctions), inhibiting heparanase or administering a thrombolytic agent (Aceto et al., 2014; Choi et al., 2015; Lu, Zeng, Gu, & Ma, 2015; Wei et al., 2018) showed promise, but concerns regarding the side effects of such treatments have been raised (Choi, Yoon, & Yun, 2016; Mirshahi et al., 2016).

Nevertheless, considering the clinical significance of CTC clusters, it is important to develop strategies that specifically target them as a means to prevent and reduce the negative effects of metastasis (Harryman et al., 2016). However, research on the potential of CTC clusters as therapeutic targets is hampered by the fact that their detection, isolation and propagation remain challenging (Alix‐Panabières, Bartkowiak, & Pantel, 2016; Au et al., 2017; Hamilton, Burghuber, & Zeillinger, 2015). To mitigate these challenges, we have recently been proposed that available established cell lines that grow as clusters in suspension can be used as in vitro “surrogates” to explore the biology of CTC clusters and develop prospective therapeutic strategies against them (Jong, Chan, & Nedelcu, 2019; May, Crawford, & Nedelcu, 2018).

Here, we suggest a complementary approach using mechanistic agent‐based models (ABMs) and an evolutionary/ecological perspective to investigate the effect of different microenvironmental challenges (e.g. resource availability, environmental threats—such as drugs or immune predation) on the resilience (cell survival) and stability of CTC clusters. The goal of this approach is to make predictions as to the combination of factors and parameter values that can reduce cell fitness and directly decrease the size of CTC clusters or induce their dissociation into smaller clusters or single CTCs, as a strategy that could lower the metastatic potential of CTC clusters. Furthermore, given the variability of CTC clusters in terms of both size and density (Figure 1a), we consider the effect of microenvironmental challenges on clusters of different sizes and densities and how such differences can affect the metastatic potential and response to therapies of CTC clusters. These predictions can then be tested both in vitro and in vivo using available cell lines that grow as clusters of various sizes and densities (Figure 1b). Combining these two approaches could help direct the development of new therapeutic strategies to increase survival prognosis in cancer patients by specifically targeting CTC clusters and their role in metastasis.

## 2 METHODS

### 2.1 The Model

Our model is a mechanistic agent‐based model (Railsback, Steven, & Grimm, 2012) designed to help understand the dynamics of circulating tumour cell cluster populations in various biologically relevant environments characterized by several factors that can vary in their parameter values. The goal of the model is to (a) understand how cluster size and cell survival are affected by different combinations of environmental and biological parameters, and (b) identify the best strategies that can decrease the size (i.e. number of cells) of CTC clusters directly or indirectly—by maximizing cluster disruption and dissociation in smaller clusters.

**Figure 2:**
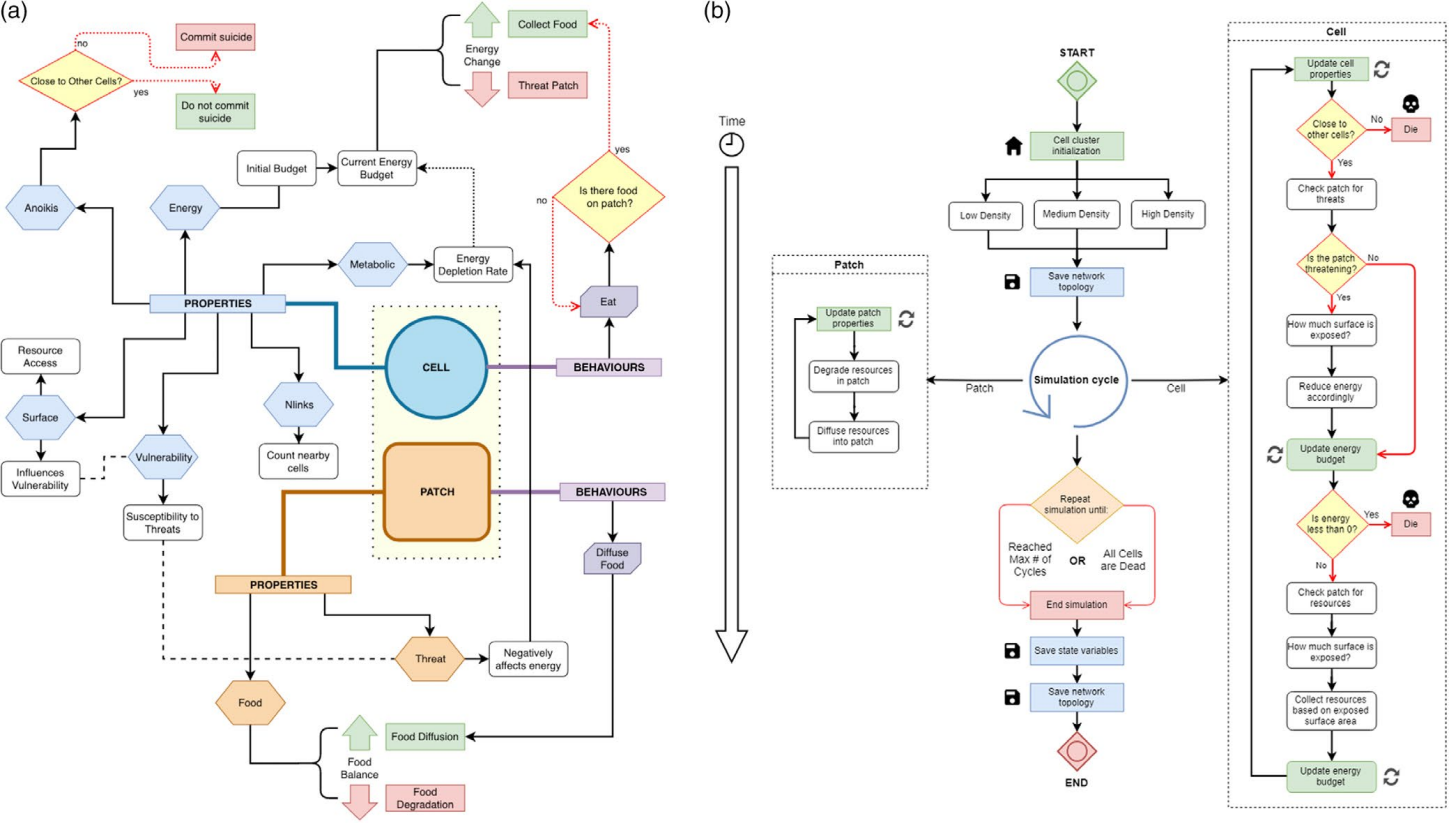
A schematic representation of the (a) properties and behaviours expressed by the two agents (cells and patches) in the model, and (b) flow chart of the simulation showing specific behavioural modules of cells and patches. For (a), cell properties are shown in blue hexagons (anoikis, energy, metabolic, Nlinks, surface and vulnerability), with their downstream effects linked via black arrows to white boxes. Patch properties are shown in orange hexagons (danger and resource). Agent behaviours are shown in purple notched boxes, with their downstream consequences linked via black arrows. Decision events are shown in yellow diamonds, with their subsequent decisions and effects linked via green or red dotted lines. Properties that are subject to fluctuations during the model are indicated with branching black lines (resource balance and energy change). For (b), agent status updates are indicated in green boxes, generalized agent actions are shown in white boxes, saved states are shown in blue boxes, termination events are shown in red boxes, and decision events with branching outcomes are shown in yellow diamonds, as in (a). Both the patch and cell behavioural modules are run concurrently as the simulation proceeds through each cycle, and the overall simulation terminates either when it reaches a maximum predetermined cycle number or when all cells are dead

Specifically, this model investigates the complex, potentially nonlinear dynamics emerging from interactions between two types of agents: (a) cells in clusters exposed to a specific environment and (b) patches where clusters reside. Agents do not move; rather, agents experience changing resource levels in their environmental patches, which is assumed to be analogous to agents experiencing new environments (in different body areas) with different resource levels. Each cellular agent has a metabolic parameter—which is an individual cell property affecting the way cells can get energy from surrounding resources, and an energy budget that allows cells to survive under optimal conditions (cells do not reproduce). Cellular agents spend additional energy when faced with potential environmental threats (immune system, drugs, etc.) and can acquire new energy from the resources available on patches. Both cells and patches are characterized by several specific properties and behaviours (Figure 2a). The model also investigates the specific spatial configurations (i.e. network topologies) resulting from cell–patch interactions over time. The environment is a torus of 33 × 33 patches (i.e. 1,089 patches), and the relative size of cells is 0.5 on both dimensions (x‐axis and y‐axis), which means that a cell is 0.25 the size of a patch. A detailed protocol following the well‐established standard of ODD (Overview, Design concepts and Details) for agent‐based models (26) and a table including the list of the model's parameters, value range and initial values (Table S1) are presented in Section 1 of Supporting information. The model is implemented in NetLogo v. 6.0.1 and freely available on the GitHub webpage (Wilensky, 1999); https://github.com/mcampenni/abm_cell_clusters/.

#### 2.1.1 Agents’ properties and behaviours

Below we provide brief descriptions of the parameters and the equations used in the model. Additional information and detailed justifications for the specific equations are presented in Section 2 of Supporting information.

##### Cell properties

Cells are defined by a set of properties:

- “Surface” is a property that reflects the cell surface area in contact with the environment (see Equation 1); it affects the amount of resources the cell has access to and how much the cell is affected by the “Vulnerability” property.

(1) $$surface_{x_{t}}\;=\;\frac{1}{links_{x_{t}}\;+\;1}$$

where *x* is the current cell agent, *t* is time, and *links* is the number of links the current cell agent has with other cell agents.

- “Vulnerability” determines how susceptible cells are to environmental threats; it is a nonlinear (sigmoid) function of the “Surface” property (see Equation 2 for details).

(2) $$vulnerability_{x_{t}}\;=\;\frac{1}{\left(1\;+\;e^{-(\left(\left(surface_{x_{t}}\;*a\right)-1\right)*b)}\right)}$$

where *x* is the current cell agent, *t* is time, surface is the current surface of the cell agent exposed to the environment, and *a* and *b* are constants.

- “Energy” is a property describing the energy budget that each cell is equipped with at the beginning of each run of simulation, and it is constantly updated at each step of the simulation. “Energy” increases when a cell acquires resources and decreases when a cell is affected by threat in a local patch (see Equation 3).
- “Metabolic” is a property that models the metabolic rate of each cell.
- “Nlinks” reflects the number of physical links that each cell has to other cells in a cluster (i.e. the number of other cells the current cell is linked to); cells are considered linked if the spatial distance between each other is below a simple threshold value.
- “Anoikis” is a property that determines the tendency of cells to commit suicide when they become physically isolated from other cells.

##### Cell behaviours

The behaviour of each cell is governed by “Eat”—which allows cells to collect energy from resources in the patch in which they currently reside.

(3) $$energy_{x_{t+1}}\;=\;energy_{x_{t}}\;+\;\left(resource_{y_{t+1}}\;*\;metabolic_{x}\;*\;surface_{x_{t+1}}\right)\;-\;\left(threat_{y}\;*\;vulnerability_{x_{t+1}}\right)$$

where *x* is the current cell agent, *t* is time, *y* is the current patch, *metabolic* is an invariant property of the cell agent, and *surface* is a property of the cell agent updated considering the current number of other cell agents the current cell agent is linked with; *threat* is the invariant threat effect of the current patch *y*, and *vulnerability* is the current vulnerability of cell agent *x*.

##### Patch properties

Patches are defined by two properties: “Threat”—a property that models the effect that the patch has on the energy level of any local cell; and “Resource”—a property that models the current availability of resources on the patch. “Resource” varies over time, as it can decrease due to degradation or increase due to diffusion from adjacent patches (see Equation 4 for details).

(4) $$resource_{y_{t+1}}=resource_{y_{t}}-\left(resource_{y_{t}}*d\right)+f\left(resource_{y_{t}},resource_{neighbors_{t}},k\right)$$

where $resource_{y_{t+1}}$ is the amount of resource available at time *t + *1 on the current patch *y*, *d* is a parameter of resource degradation over time, and *f* is a diffusion function incrementing the amount of resource available at time *t* on the patch *y* by a proportion *k* of the amount of resource available at time *t* on the eight neighbouring patches.

##### Patch behaviour

The behaviour of each patch is governed by “Diffuse‐resource”—a behaviour that provides new resources for cells to consume.

#### 2.1.2 Process overview and scheduling

The model consists of several submodels (Figure 2b), which include: (a) initialization of the environment to determine the initial availability of resources and local threat in patches; (b) initialization of the cells and their properties; (c) the behaviour of cells, which consists of: collecting resources, checking the effects of the local threat level and checking if a critical threshold is reached to trigger cell death; and (d) finally, updating, for each patch, the availability of resources diffused at a regular intervals from surrounding patches. There is no input from external sources used in the model. At the onset, a particular environment is initialized in a 2‐dimensional grid of patches, and a cluster of cells is generated based on a specific size (*N* = 20, 40, 80, 100 cells) and density (low, medium and high) centred around the middle of the grid. Then, the particular initial spatial configuration of the cluster (i.e. the network topology) is saved, as clusters do not migrate around the grid individually. The initial network topologies are not specified. Rather, the initialization procedures create a cluster of cells taking into consideration a given set of parameters about group size and cluster density. In other words, each initial cluster of cells is basically a random network of cells with varying number of connections to other surrounding cells. The specific network topology emerging at the end of each simulation is the result of the response of the system to a specific combination of environmental resource availability and threats.

A typical run of the model starts with the degradation of local resources and the update of cells’ properties (e.g. energy budget, surface in contact with the environment) based on their patch characteristics. Then, cells check whether there is a local threat affecting their energy or not (threat affects cells in accordance with the actual surface they expose to the environment), which determines whether they die or not. Cells die as soon as they reach the critical energetic threshold of zero, or due to the induction of anoikis (when there are no other cells in a given distance). Surviving cells have access to environmental resources in accordance with the actual surface they expose to the environment. After a fixed period of time, new resources are available to cells. The run ends when the model reaches a given time “*t*” or when all cells have died. At the end of the run, information about state variables (N, density, availability of resources, patches’ threat, median energy of cells, median availability of resources and the median metabolic rate of cells) is saved. Moreover, the particular spatial configuration of the remaining clusters (i.e. the network topology) is saved.

### 2.2 Data analyses

For each combination of the parameters, we considered median values over all runs and used those values to calculate the survival ratio and stability index; data are presented as boxplots. Survival ratio (SR) is defined as the number of surviving cells at the end of the simulation (*N_final_*) relative to the number of cells at the beginning of the specific simulation run.

$$SR\;=\;\frac{N_{\mathrm{final}}}{N_{\mathrm{initial}}}$$

Stability index (SI) represents the proportion of surviving cells normalized by the number of components (i.e. the number of unconnected subnetworks: a single isolated node is itself a component, and a network that is itself connected has exactly one component, consisting of the whole network).

$$SI\;=\;\frac{\left(\frac{N_{\mathrm{final}}}{N_{\mathrm{initial}}}\right)}{\mathrm{components}}$$

Or

$$SI\;=\;\frac{\mathrm{SR}}{\mathrm{components}}$$

**Figure 3:**
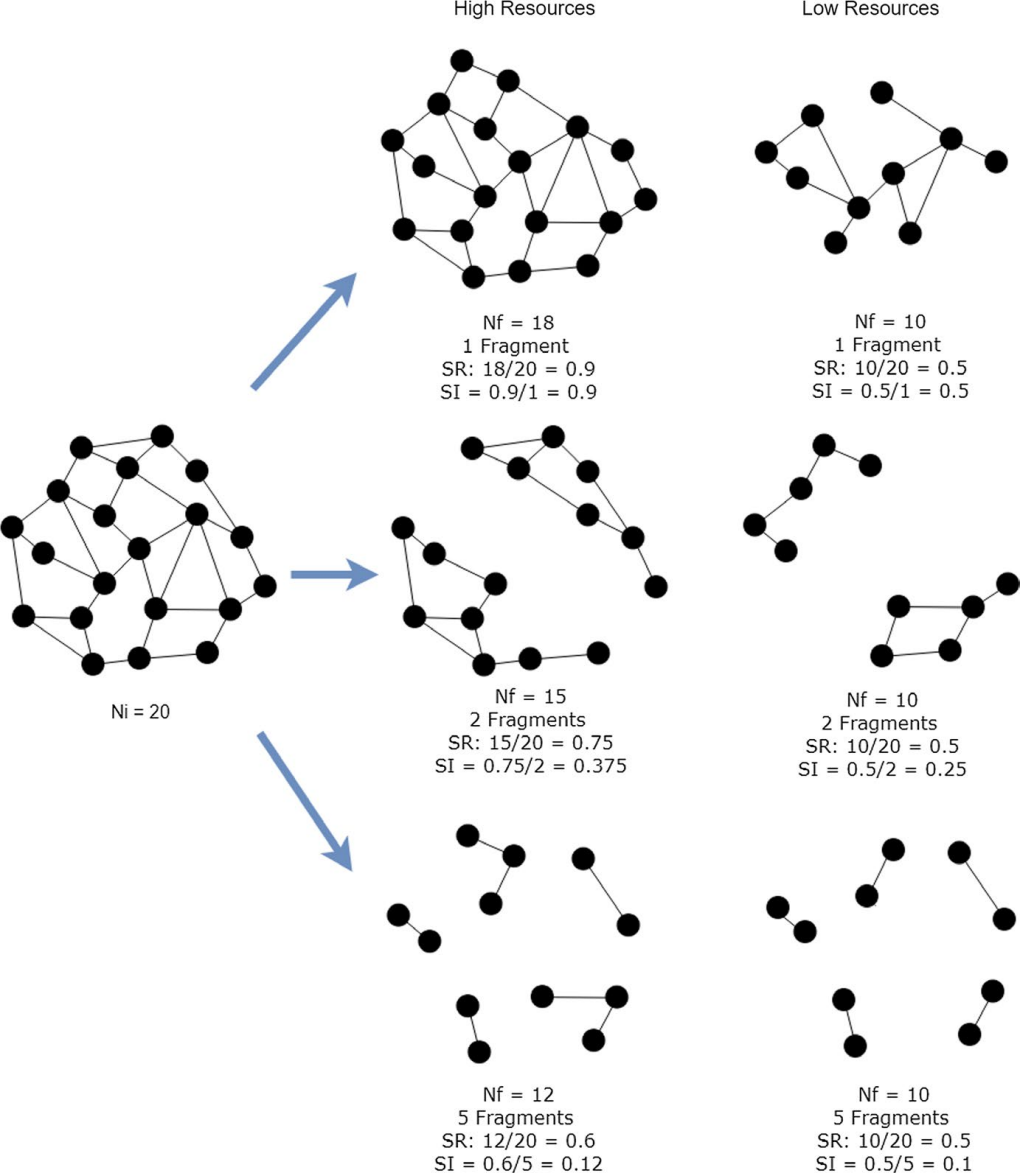
Hypothetical scenario displaying several responses of a 20‐celled cluster (Ni—initial number of cells) to high‐ and low‐resource environments, and resulting in outcomes with various final total number of cells (Nf), survival ratios (SR) and stability indexes (SI)

These two measures are similar with respect to the type of information they take into consideration, but they differ in their meaning. While the survival ratio provides a measure of the resilience of the system/cluster in terms of the survival of cells over the simulated process, the stability index is a measure of the survival of cells normalized by the specific topological configuration of the considered cluster. Thus, clusters with the same *N_inital_* and *N_final_* that exhibit different final different topological configurations will be characterized by the same survival ratio, but different stability indexes (see examples in Figure 3). The difference between SR and SI is indicative of the degree of fragmentation; the more different SR and SI are, the higher the dissociation of the cluster.

To evaluate possible correlations and interplay between different parameters, we ran separate analyses considering one of the parameters as independent variable, the measures we defined (i.e. survival ratio, stability index) as dependent variables and other parameters as parameters defining particular experimental settings (see Results for details). Analyses were performed using R version 3.5.3 (2019‐03‐11) and Tidyverse, which is a collection of R packages designed for data science (R Core Team, 2019; Wickham, 2017).

## 3 RESULTS

### 3.1 Overview

The goal of our mechanistic agent‐based model is twofold: (a) to investigate the response of clusters of various densities and sizes to different environmental conditions, and (b) to find the combination of factors (i.e. various levels of environmental threats and resources) that is most effective at decreasing the survival of cells (and thus cluster size) in clusters with various initial sizes and densities. We have run the model varying four different parameters: cluster density, cluster size, threat intensity and availability of resources; for each specific combination of these parameters, we have run the model 1,000 times (i.e. runs or replicates) totalling 720,000 simulations. Below we present the most relevant findings for a subset of the parameter values used (see Section 3 of the Supporting information for additional data). The data presented here reflect the models’ results at the equilibrium, that is at the end of the simulations when the model reached the steady state. The analyses of the dynamics of the system (i.e. how models’ outputs change over time) are included in Section 4 of the Supporting information.

To allow biologically relevant comparisons among results using various combinations of parameters and values, we expressed the outcome of all simulations in two ways. First, we calculated the survival ratios, that is the number of surviving cells at the end of the simulation relative to the initial number of cells in a cluster (see Methods). Higher values (i.e. higher survival ratios) are indicative of more resilient clusters. For clusters subjected to different environmental challenges (high threats or low resource levels), higher values are also indicative of better persistence in those conditions. Second, because at the end of the run the surviving cells might be distributed among more than one cluster (due to the fragmentation of the initial clusters as internal cells die), we also calculated a stability index, specifically, the proportion of survival cells normalized by the number of cluster fragments (see Methods). For clusters with the same survival ratio, a higher stability index is indicative of clusters with lower propensity to dissociate (see example in Figure 3). From a biological perspective, a higher stability index implies increased stability or resistance to treatment, which could reflect in overall higher metastatic potential.

Results sections below highlight a subset of analyses that address the impact of initial cluster size and density on cluster resilience and stability in (a) optimal environments, (b) low‐threat environments with variable levels of resources, (c) rich environments with variable threat levels and (d) environments with variable levels of both resources and threat. Both survival ratios and stability indexes were calculated for different combinations of parameters (see Section 3 of Supporting information for additional analyses).

### 3.2 The impact of density and size on cluster resilience and stability in optimal environments

**Figure 4:**
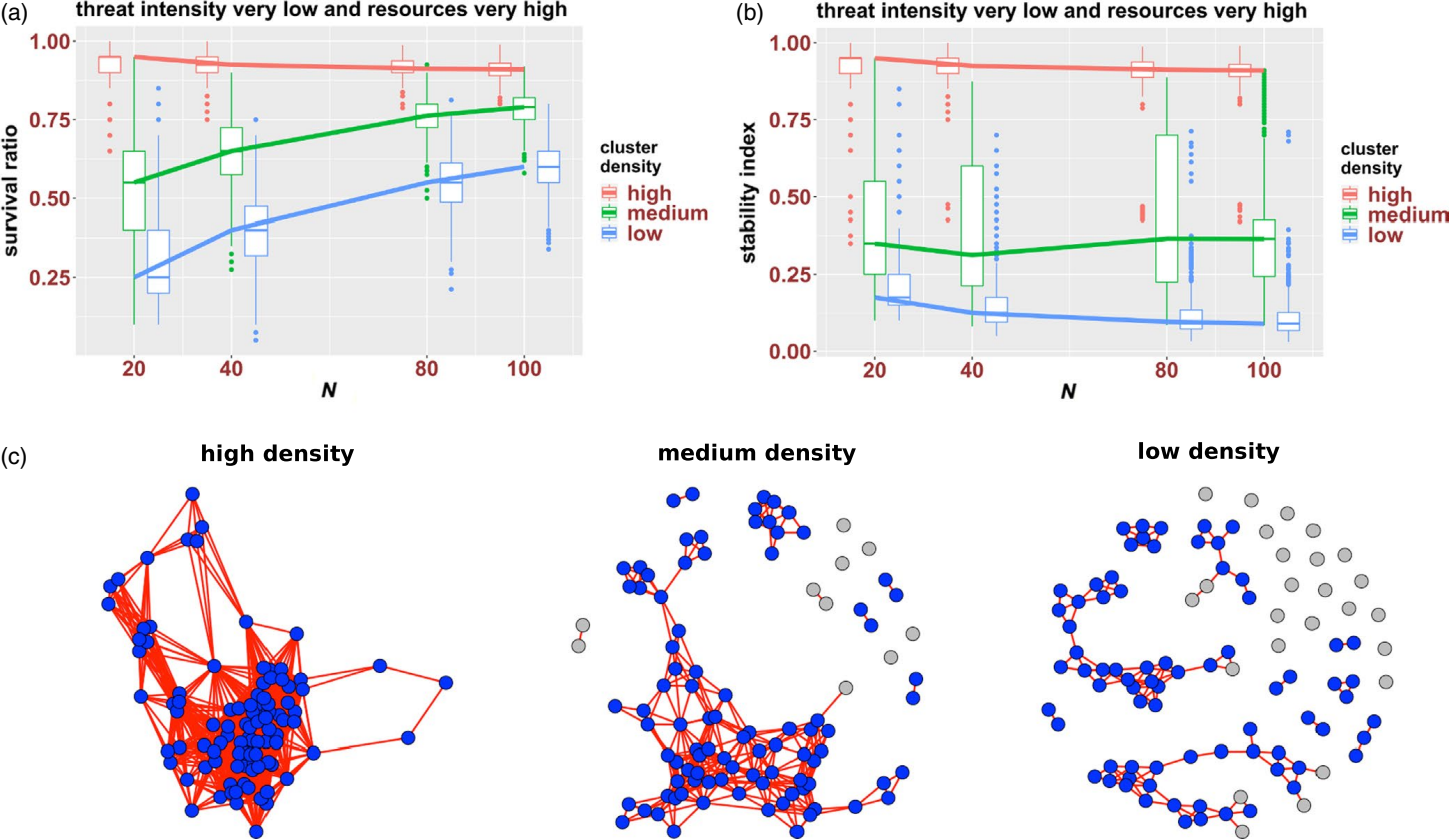
The impact of cluster density and size on the survival ratios, stability index and network structures of high‐, medium‐ and low‐density clusters in very high‐resource environments with low threat levels. Data on survival ratios—(a) and stability index—(b) of high‐, medium‐ and low‐density clusters of 20, 40, 80 and 100 cells are presented as boxplots, where the box includes the first, second (i.e. median) and third quartile of the data; bars show minimum and maximum values, and outliers are represented as dots. Solid lines link the median values of each boxplot (corresponding to a value on the x‐axis) for the different box‐plotted data points. Network structures in panel C are representative examples of the final configurations of single runs for 100‐cell clusters of high, medium and low density; networks were displayed using the Fruchterman–Reingold force‐directed algorithm, which distributes nodes in space using attraction and repulsion forces (Fruchterman & Reingold, 1991). Blue and grey nodes represent surviving and dead cells at the time the networks were sampled. Nodes that correspond to cells separated by a distance equal or <1 patch (i.e. the two cells are in a radius of 1 patch) are linked by a red line; nodes that correspond to cells farther apart—as in medium‐ and low‐density clusters, are not connected. The length of a connection is proportional to the strength of the degree of the nodes (i.e. the number of connections with other nodes), not to the actual distance between cells in a cluster

The first set of simulations addressed the resilience and stability of clusters of various densities and sizes in environments with very low threat and very high resource availability. We found that both cluster density (high, medium, low) and initial size (*N* = 20, 40, 80, 100) influenced survival ratios and stability indexes, but in different ways (Figure 4).

Survival ratios were strongly affected by cluster density and size. High‐density (HD) clusters were more resilient than medium‐density (MD) and low‐density (LD) clusters, for all size classes; and differences in density impacted most the SR of smaller clusters (Figure 4a). Furthermore, the initial size of the cluster had no impact on the resilience of HD clusters (i.e. survival ratios are very similar for all size classes); however, it did impact the survival ratios of MD and LD clusters, with large clusters being more resilient than small clusters of the same density (Figure 4a).

In contrast to SRs, stability indexes were only affected by cluster density. The initial cluster size did not have an impact on the stability index of either HD or MD and LD clusters; clusters of same density but of different sizes had similar stability indexes (Figure 4b). However, density had a strong impact on the SI, with HD clusters having much higher stability indexes than MD and LD clusters, for all size classes (Figure 4b). Overall, these analyses indicate that cluster stability is strongly dependent on density but not size.

Comparisons between survival ratios and stability indexes reveal some interesting differences among clusters (Figure 4a and b). While for HD clusters, the two measures are rather similar (suggesting a low degree of fragmentation), for MD and LD clusters, SIs were much lower than SRs (suggesting a high degree of dissociation; see Figure 3 for examples). Furthermore, among MD and LD clusters, the difference between SRs and SIs (and thus the degree of fragmentation) is higher in larger clusters. For instance, the SRs of 100‐cell and 20‐cell MD clusters are ca. 0.75 and 0.50, respectively, while the stability index is around 0.25 for both cluster sizes. Higher degrees of fragmentations in larger clusters (relative to smaller cluster) are to be expected as—all else being equal, the larger the cluster, the higher the possible number of fragments. This is because the highest number of fragments for any cluster equals N/2 (2 is the smallest size possible of a fragment, as single cells are expected to undergo anoikis). However, similar stability indexes for clusters of different sizes indicate proportional propensities to dissociate.

### 3.3 The effect of resource availability on cluster persistence and stability

**Figure 5:**
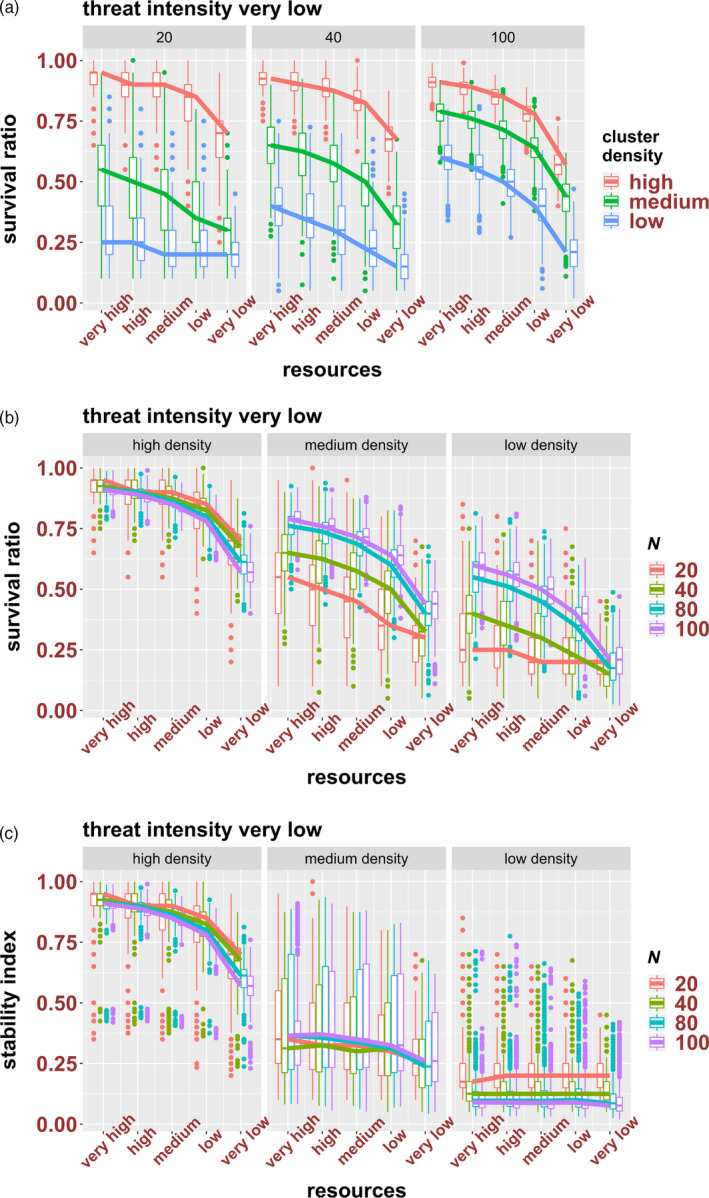
The effect of resource availability on the survival ratios (a and b) and stability index (c) of high‐, medium‐ and low‐density clusters of 20, 40, 80 and 100 cells; see Figure 4 for explanation of the plots

In the second set of simulations, we investigated the SRs and SIs of clusters of various densities and sizes subjected to different levels of resources (very low, low, medium, high, very high), in environments with very low threat levels. Although resource availability affected the survival of all clusters, the effect was dependent on cluster density and size. Overall, HD clusters were more resilient than MD and LD clusters, irrespective of cluster size and resource availability, and differences in density were most relevant to smaller clusters (Figure 5a). In terms of size, the response of HD clusters was least affected by initial size (i.e. for a specific resource level, SRs are similar among clusters of different sizes), while size did affect the response of MD and LD clusters, with larger clusters being more resilient than smaller clusters of the same density (although differences between large and small clusters were less evident at low resource levels) (Figure 5b).

Comparisons between SRs (Figure 5b) and SIs (Figure 5c) revealed again interesting differences among clusters of different densities. The SRs and SIs are similar for HD clusters (for all size classes and resource levels), indicating that they incurred low levels of fragmentation. On the other hand, SIs are much lower than SRs for MD and LD clusters, suggestive of high degrees of dissociation for these clusters, for all class sizes and resource levels. Thus, for MD and LD clusters, although larger clusters and/or higher resource availability can result in an overall higher proportion of surviving cells, the dissociation of clusters can drastically impact the final composition of the populations. Furthermore, similar SIs for clusters of the same density but of different sizes and at different resource levels (Figure 5c) indicate that dissociation levels are not affected by cluster size or resource levels; they are an intrinsic property of clusters of a specific density (i.e. they are only affected by density; as in Figure 4d).

### 3.4 The effect of environmental threats on cluster persistence and stability

**Figure 6:**
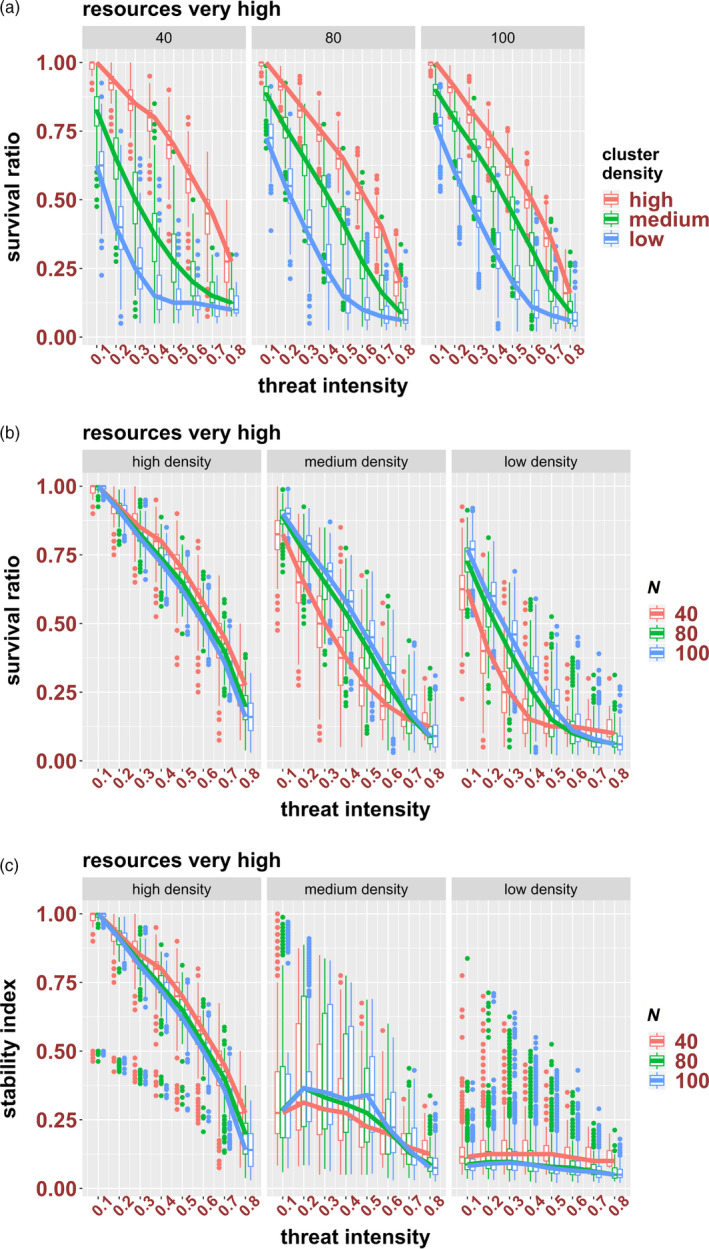
The effect of threat intensity on the survival ratio (a and b) stability index (c) of high‐, medium‐ and low‐density clusters of 40, 80 and 100 cells; see Figure 4 for explanation of the plots

To investigate the response to environmental threats, clusters of different densities and sizes were subjected to various levels of threat (0.1–0.8) at very high resource levels. As expected, the survival ratios of clusters were dependent on the level of environmental threat, with higher levels of threat resulting in lower SRs. However, the response was also dependent on the density and size of the cluster. Specifically, HD clusters were most resistant to threat, irrespective of their size and threat level, and differences in density impacted most the response of small clusters (Figure 6a). In terms of size, the response of HD clusters was not impacted by initial size (for a specific threat level, SRs are similar among clusters of different sizes), but for MD and LD clusters, larger clusters were generally more resistant than smaller clusters (Figure 6b).

Similar to what we observed for the effect of resource availability on cluster stability, the SRs and SIs are similar for HD clusters—indicating that they incurred low levels of fragmentation, while SIs are much lower than SRs for MD and LD clusters—suggestive of high degrees of dissociation for these clusters (Figures 6b vs 5c). However, in contrast to resource availability, threat intensity appears to have an effect on the dissociation of the MD clusters, as SIs seem to be decreasing as threat intensity increases (Figure 6c).

### 3.5 The combined effect of environmental threats and resource availability on cluster survival

**Figure 7:**
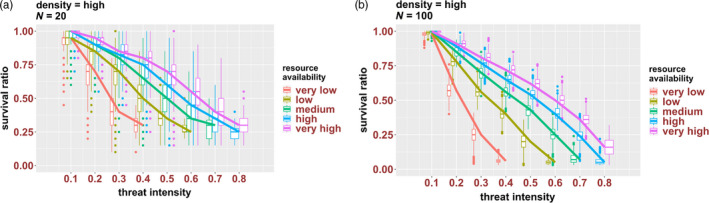
Combined effects of resource availability and threat intensity for (a) 20‐cell HD clusters and (b) 100‐cell HD clusters; see Figure 4 for explanation of the plots

In the last set of simulations, we have compared the survival ratios of clusters subjected to various levels of threat or resource availabilities alone with those of clusters experiencing various combinations of both factors. Overall, we found that simultaneously applying threat and reduced resource availability can lower cluster survival ratios more than each of the factors individually (see Figure S4 in Supporting information). This is extremely important for HD clusters, which tend to be more resilient and more stable than MD and LD clusters, irrespective of size (e.g. Figures 4a and b, and 5a). For instance, for a small size HD cluster of 20 cells, a medium threat level of 0.4 in a high‐resource environment can only reduce its survival ratio to ca. 0.75. However, the same threat level applied at low resource availability can reduce its survival ratio down to ca. 0.50 (Figure 7a). Similarly, for a 100‐cell HD cluster, the same combination of threat (0.4) and low resource levels can decrease its survival ratio to ca. 0.40, compared to only ca. 0.70 when subjected to threat alone at high resource levels (Figure 7b). Conversely, resource deprivation alone (very low resource levels) has a weak effect on the SRs of both 20‐ and 100‐cell clusters (SR between 0.9 and 1), but it can fully eliminate both cluster types when combined with medium threat levels (0.5) (Figure 7).

## 4 DISCUSSION

The model we presented here was designed to investigate the response of cell clusters of various sizes and densities to environments with various levels of resources and threat intensities, with the goal of identifying the set of conditions that would maximize cluster size reduction directly (through cell loss) and/or indirectly (through cluster dissociation). In theory, cluster dissociation could result in improved dispersal through increasing the number of clusters. But the decreased size of the fragments will negatively affect the likelihood of these clusters to survive and establish new tumours. Indeed, although large clusters are less frequent compared with small clusters (e.g. Bocci, Jolly, & Onuchic, 2019), studies (including longitudinal analyses; i.e. enumeration of CTC and CTC clusters at baseline and follow‐ups) have shown that the presence of larger‐size CTC (>3 cells) confers the highest risk of death (Wang et al., 2017). Changes in the size and density of the CTC clusters in response to therapy have been recently reported. For instance, it has been shown that chemotherapy reduced not only the number but also the size of CTC clusters in a patient with ovarian cancer (Meunier et al., 2019). Also, successful therapy was correlated with loose cluster formation, while tight clusters correlated with therapy resistance (Balakrishnan et al., 2019).

Because the aim of the model is to help develop strategies to reduce the size of real CTC clusters, we have used agent properties, parameters and parameter values that are biologically relevant. However, we are not implying that the values we used should be directly applied (or assumed to correspond exactly) to biological contexts. Rather, the interpretation of the model will be focused on general trends and findings that can be tested in vitro and/or vivo. We are also aware that, for simplicity, a number of other potentially relevant aspects (such as flow or fluid dynamics and the presence of noncancer cells in clusters) have not been incorporated in the current model. Below we discuss the main findings of our model and their relevance to therapy. We also highlight the general significance and future applications of agent‐based models to improving our understanding of the behaviour of CTC clusters and the development of strategies to decrease their metastatic potential. Finally, we consider ways to test the predictions of our model and suggest future directions.

### 4.1 Main findings of the model and therapeutic implications

Understanding the behaviour of CTC clusters of different densities/sizes and the effect of resource availability and drug levels on their resilience and stability has significant clinical and therapeutic significance. If differences in CTC cluster size and density correlate with clusters’ metastatic potentials, these two parameters can have important prognostic values. Furthermore, understanding the differential response to resource availability and threat levels of clusters of various densities and sizes will allow the development and administration of more individualized therapies directed at decreasing the metastatic potential of specific CTC cluster types with fewer side effects.

#### 4.1.1 Both size and density might affect the metastatic potential of CTC clusters

Early experimental studies in mice found a link between the size of cell clusters released from tumours and their metastatic potential (Liotta et al., 1976), and more recent studies correlated the size of CTC clusters with poor prognosis (Wang et al., 2017). Our simulations indicate that for MD and LD clusters (but not HD clusters), larger clusters are indeed more resilient (and thus, possibly more aggressive) than smaller clusters of the same density. However, in addition to size, the resilience of clusters is also strongly affected by their density. Specifically, under optimal conditions (i.e. high resource and low threat levels), HD clusters are more resilient than MD and LD clusters, for all size classes (likely due to increased cell–cell contact area, which lowers the propensity of cells to undergo anoikis).

Information on differences in density among CTC clusters is currently limited. However, available images of isolated CTC clusters suggest that such differences do exist (Figure 1a). Furthermore, although it is not clear how variable cluster densities are, and whether different types of cancer or individuals exhibit specific CTC cluster densities, a recent study observed predominantly dense/tight clusters in breast and lung cancer patients, but more loose clusters in patients with oesophageal and bladder cancers (Balakrishnan et al., 2019). Likewise, although it also remains to be determined whether the density of CTC clusters does correlate with their metastatic potential, the same study found that the presence of tight clusters was associated with shorter patient survival (Balakrishnan et al., 2019). To further support such a correlation, two cell lines derived from cancers with different aggressivity levels appear to form clusters of different densities: compact clusters in a very aggressive small cell lung cancer line (H187), and less dense clusters in a less aggressive non‐small‐cell lung cancer line (H2122) (Figure 1b).

Our simulations also showed an important interaction between cluster size and density. Specifically, while size has a strong impact on the resilience of low‐ and medium‐density clusters (with larger clusters being more resilient than smaller clusters of the same density size), size has little or no impact on the resilience of high‐density clusters (both small and large HD clusters are quite resilient). The latter finding implies that the dissociation of large HD clusters into smaller clusters might be, by itself, less efficient in decreasing their metastatic potential. Also, when compared to small clusters, the resilience of large clusters is less impacted by their density. These effects are likely related to the propensity of MD and LD clusters to dissociate easier, as well as to the increased likelihood of smaller clusters to dissociate into single cells that will undergo anoikis.

#### 4.1.2 Stability might be as important as resilience when assessing the metastatic potential of CTC clusters

As cluster size is thought to correlate with aggressivity, strategies that can reduce their size directly or indirectly (by inducing their dissociation into smaller clusters and—ultimately, single CTCs) could contribute to decreasing their metastatic potential. Our model showed that density can greatly impact the stability of clusters of all sizes. Specifically, HD clusters are much more stable and difficult to dissociate than MD and LD clusters, regardless of their initial size. This resilience to dissociation is likely linked to their overall high survival ratios (irrespective of size) and increased survival relative to same‐size clusters of lower density. Note that in our model, cluster dissociation is strictly dependent on cell death (i.e. fragmentation is strictly due to the death of internal cells). The potential of cell death to result in cluster fragmentation (as well as a positive correlation between cell death and fragmentation rates) has been previously demonstrated in experimentally evolved multicellular clusters/flakes in yeast (Ratcliff, Denison, Borrello, & Travisano, 2012).

The complex effects that cluster density and size showed on both the resilience and stability of the clusters are likely to have significant consequences for the metastatic potential of CTC clusters and their responses to therapies. Furthermore, our findings suggest that, at least for MD and LD clusters, strategies that might not have a strong negative effect on their overall survival ratio could still decrease their metastatic potential by inducing cluster dissociation. Conversely, strategies that could have a strong negative effect on survival ratios might be less effective than strategies that affect less the overall proportion of surviving cells, but induce higher cluster dissociation. Thus, survival ratios by themselves might not necessarily be the best predictors of the metastatic potential of CTC clusters. Consequently, in vitro testing of therapies designed to specifically target CTC clusters should take into account both parameters.

#### 4.1.3 Proposed fasting‐based therapies can have differential effectiveness on CTC clusters

Several starvation/fasting‐based therapies have been proposed to be efficient at decreasing tumour burden and mitigating the side effects of chemotherapy (Lee et al., 2012, Nencioni, Caffa, Cortellino, & Longo, 2018, Raffaghello et al., 2008). Although the effect of such therapies on CTC clusters has not been investigated, our findings suggest that the outcome might be influenced by the density and size of CTC clusters. As expected, low resource levels negatively impacted the survival ratios of all clusters. However, our simulations showed that resource levels have a differential effect on the survival of clusters of different densities and sizes. For instance, HD clusters are more resistant than MD and LD clusters in all resource levels and for all size classes (likely due to increased anoikis and cluster dissociation in lower‐density clusters), and among MD and LD clusters, large clusters survived better than small clusters in all resource levels (also due to increased likelihood/rate of anoikis in small clusters as the number of connections is lower, especially as clusters start dissociating). Thus, HD clusters and large MD and LD clusters might still be surviving well even at low resource levels. Note that in our model, resources (even at the lowest levels) are not limiting. That is, low resource levels by themselves do not trigger death; it is only the combination of factors that decrease the energy budget below a threshold that can trigger death. Furthermore, the efficiency of reducing resources (as denoted by changes in survival ratio from very high to very low resource levels, i.e. the slopes in Figure 5a and b) can be impacted by cluster density and size. For instance, lowering resource levels is likely to have the strongest effect (in terms of efficiency in decreasing the survival of a particular of cluster type, i.e. within‐group comparison) on large LD clusters, due to both higher competition for resources and increased anoikis. Conversely, small LD clusters are least affected by changes in resource availability; thus, fasting‐based therapies might be less efficient in cases associated with small low‐density CTC clusters.

#### 4.1.4 “One size fits all” might not be the best strategy in terms of clusters’ responses to drugs

The threat parameter we have implemented in our model can be interpreted as any extrinsic factor that can negatively affect the survival of clusters, from cytotoxic drugs to the immune system. Since CTC clusters are known to be refractory to treatments (Hou et al., 2012; Kaushik, Yakisich, Way, Azad, & Iyer, 2019; Krebs et al., 2012), the development of strategies (chemotherapies or immunotherapies) directed at specifically targeting them is of great significance. As expected, our model showed that threats are most effective on LD clusters, especially those of smaller sizes. However, the model can also provide insight into drug–response dynamics, that is the relationship between changes in threat intensity and changes in survival. For instance, our simulations suggest that increasing the intensity of threat is, generally, least effective (in terms of additional decrease in SR with increase in threat level, i.e. slopes in Figure 6a and b) on HD clusters. Also, LD clusters (especially of small size) tend to reach a plateau whereby increases in threat intensity are not associated with a proportional decrease in survival ratios.

Investigations into the specific responses of clusters of different size and density to drug levels can have significant therapeutic significance as they can direct more effective treatments (in terms of dosage) based on the specifics (density and size) of CTC clusters associated with a particular type of cancer or a specific individual. For instance, a dosage level that is required to decrease the survival of HD and/or large MD and LD clusters might be unnecessary for small LD clusters. Such information would avoid unnecessary negative side effects of therapies based on agents that can also affect healthy cells (like most standard chemotherapies). The phenotype of CTC clusters (pre‐ and posttherapy) has recently been proposed to guide and select specific drugs for more effective personalized treatments (Balakrishnan et al., 2019).

#### 4.1.5 Combination therapies can be more effective at decreasing the metastatic potential of CTC clusters

Combination therapies (i.e. various combinations of surgery, radiotherapy, chemotherapy, immunotherapy) have been successfully used in decreasing tumour burden (Mokhtari et al., 2017). Our model shows that combining various levels of resources and threat intensity can also be more effective at decreasing the survival of CTC clusters than each factor alone. For instance, while decreasing resource availability or increasing the threat level alone can decrease the survival of clusters (especially those of smaller size and/or lower density), applying both pressures can result in better outcomes, especially for large and HD clusters. Such additive or synergistic effects might allow for the development of new therapeutic strategies that can achieve improved outcomes by combining different factors (resource levels and chemotherapeutic agents; different chemotherapies; chemo‐ and immunotherapy), each of which acting in a different way. As CTC clusters are refractory to many chemotherapies, such combinations might have the same effect as administering higher doses of one specific drug and thus could reduce drug toxicity and negative side effects. Agent‐based models can direct the development of such therapies.

### 4.2 The utility of mechanistic agent‐based models for cancer research

A mechanistic model assumes that a complex system can be understood by examining the workings of its individual parts and the manner in which they are coupled. Mechanistic models typically have a tangible, physical aspect, in that system components are real, solid and visible. Traditionally, "mechanistic models" are those that are based on the mathematical description of a mechanical, chemical, biological phenomenon or process. Such models can have multiple uses in cancer research: (a) they can be used to identify crucial elements and factors affecting agents’ behaviour; (b) they can be used to test theoretical hypotheses about the role and relevance of specific elements and factors by manipulating the different parameters of the model; (c) they can generate a big amount of in silico data that can be used to run analyses that otherwise it would be difficult to run with biological data (both in vivo and in vitro) because of many different reasons (ethical, financial and time–space constraints); and (d) they can be integrated with experimental work.

Agent‐based models have already been used in cancer research to address a variety of scenarios, including preventative vaccination (Palladini et al., 2010), hematopoietic cell mutations (Rodriguez‐Brenes, Komarova, & Wodarz, 2014), adaptive therapy (Gallaher, Enriquez‐Navas, Luddy, Gatenby, & Anderson, 2018), and the development of combinational immunotherapies for colorectal cancer (Kather et al., 2017, 2018). The model we presented here extends these approaches to a recently acknowledged problem of increasing interest in cancer treatment and management. We argue that ABM approaches are extremely valuable complements to current research efforts to decrease the metastatic potential of CTC clusters and improve prognosis for cancer patients. Although the model presented here does not explicitly use an evolutionary framework, it lays the ground for future studies that can take into account the well‐known genetic and phenotypic heterogeneity of cells in a CTC cluster and differences in their fitness potential.

### 4.3 Testing the predictions and assessing the utility of the model

As with all models, the main value of our mechanistic agent‐based model is that it provides a set of predictions that can be experimentally tested. We have already proposed that established cell lines that grow as clusters in suspension (such as the ones in Figure 1b) can be used as in vitro surrogates for CTC clusters to investigate their biology and direct the development of new therapeutic strategies (May et al., 2018). These cell lines produce clusters of various sizes and densities, which can enable the direct testing of the predictions of our model. For instance, the suggested correlation between the aggressivity of CTC clusters and their density can be addressed by xenografting clusters of different densities in mouse models. Similarly, resource (e.g. glucose or glutamine) and drug levels can be manipulated (alone or in combination) both in vitro and in vivo, and their effect on CTC clusters of different initial sizes and densities can be addressed.

The model can be further developed and refined to address particular aspects of the biology of CTC clusters and investigate specific responses to particular therapies. For example, we assume that cells that are not in close contact with other cells will undergo anoikis. Nevertheless, mutations in the anoikis pathway or cell plasticity (i.e. induction of the mesenchymal fate) might affect this property. The model can also be extended to include other parameters, such as drug concentration, drug degradation, fluctuations in drugs and resource availability. In addition, ecological and evolutionary principles can be applied to the model, which will provide a theoretical framework to test the variables and predictions of the model.

Extending the findings of the model (both theoretical and experimental) to real applications requires a better understanding of the characteristics of CTC clusters as they relate to specific types and cancers and patients. The increased interest in the isolation and ex vivo proliferation of CTC and CTC clusters (Alix‐Panabières & Pantel, 2014; Ferreira, Ramani, & Jeffrey, 2016; Meunier et al., 2019; Pantel & Speicher, 2016) to provide personalized information on the genetic make‐up of individual patients could also be used to address whether correlations between particular cancer types (or individuals) and specific characteristics of the corresponding CTC clusters (size and density) exist. A recent study that reports a new gravity‐based microfiltration system to capture CTC and CTC clusters also reveals interesting observations on the prevalence and size of CTC clusters in patients with different types of ovarian cancer and in different clinical stages (including before and after chemotherapy) (Meunier et al., 2019). For instance, in patients with advanced high‐grade serous carcinoma, clusters varied between 2 and 50 cells (with most clusters being of only 2 cells), whereas in patient with endometrioid carcinoma, 57% of clusters were larger than 4 cells (including clusters with more than 50 and 100 cells) (Meunier et al., 2019). However, even in the absence of such correlations, models such as the one we presented here can provide invaluable information that can be applied to increase the effectiveness of therapies to specifically target CTC clusters.

## AUTHOR CONTRIBUTIONS

MC carried out the simulations, collected the data, performed data and statistical analyses, participated in the design of the study and the drafting of the manuscript, and critically revised the manuscript. ANM participated in the design of the study, data analyses and the drafting of the manuscript and figures, and critically revised the manuscript. AB participated in the design of the study, data analyses and the drafting of the manuscript, and critically revised the manuscript. VH participated in the design of the study, data analyses and the drafting of the manuscript, and critically revised the manuscript. AMN conceived and coordinated the study, participated in the design of the study and data analyses, and wrote/finalized the manuscript. All authors gave final approval for publication and agree to be held accountable for the work performed therein.

## Supporting information

Supinfo

## Data Availability

The model and code are freely available on the GitHub webpage at https://github.com/mcampenni/abm_cell_clusters/
