# Supplementary material for: Agent‐based modelling reveals strategies to reduce the fitness and metastatic potential of circulating tumour cell clusters
Source: Evol Appl. 2020 Mar 18;13(7):1635–50. doi: 10.1111/eva.12943 (PMC7428819; doi:10.1111/eva.12943)
Supplement: Supplementary file 1 — Supinfo [file EVA-13-1635-s001.pdf]

Supplementary Material to:

Simulating the behaviour of circulating tumour cell clusters: Agent-based modelling reveals strategies to reduce their metastatic potential

Marco Campenni[1,5], Alexander N. May[2,4], Amy Boddy[3,4], Valerie Harris[4], Aurora M. Nedelcu[6*]

[1]University of Exeter, Biosciences, Penryn, Cornwall, UK
[2] Research Casting International, Quinte West, ON, Canada

[3]Department of Anthropology, University of California Santa Barbara, Santa Barbara, CA, USA

[4]Arizona State University, Biodesign Institute, Tempe, AZ, USA

[5]Arizona State University, Department of Psychology, Tempe, AZ, USA
[6]University of New Brunswick, Biology Department, Fredericton, NB, Canada

[*] Corresponding author: Aurora M. Nedelcu – anedelcu@unb.ca

In this document we present:

- the full version of the ODD (Overview, Design concepts, Details) protocol of the mechanistic agent-based model
- explanations for the equations used in the model
- supplementary analyses of the models' results at the equilibrium (i.e., at the end of the simulations)
- supplementary analyses of the dynamics of the models (i.e., results over time).

1. The ODD

The Overview, Design concepts, Details protocol (shortly, ODD - Grimm et al. 2010) is becoming the accepted standard to present and share an agent-based model.
An ODD develops around seven main points.

1. **Purpose**
    - *Question*: What is the purpose of the model?
    - This is a mechanistic agent-based model of cluster cell survival into a risky environment. This model is designed to clarify the dynamics of circulating tumor cell (CTC) clusters. The model considers two different types of agents: cells and environmental patches. It investigates the complex, potentially non-linear dynamics emerging from interactions between cells from clusters moving into a specific environment, and patches where cells reside. Both cell agents and patch agents do not move; this is because patches do not move per definition, and because we decided to model cells movement through the flow of resources around them instead of actually moving cells. Cells have an energy budget to survive and they get new energy from the resources available on patches; but they also spend energy in dealing with potential environmental threats (the threat on patches may be represented by the immune system, by toxicity induced by a treatment, etc). Both cells and patches are characterized by some properties and behaviors. We are interested in better understanding the different possible combinations of environmental/cell parameters affecting the survival of cells. This may have a big impact on the design of tumor treatment strategies, and more broadly in better understanding the behavior of clustered cells. The model also investigates the specific spatial configurations (i.e., network topologies) resulting from cell-patch interactions over time. This particular result may clarify whether there are specific spatial configurations more adaptive to risky environments and whether there are spatial configurations more prone to environmental threats.

2. **Entities, state variables, and scales**
    - What kinds of entities are in the model? By what state variables, or attributes, are these entities characterized? What are the temporal and spatial resolutions and extents of the model?
        1. Agents/individuals: cells and patches.
        2. Spatial units (e.g., grid cells); grid cells represented by patches.
        3. Environment

Patches are characterized by an individual variable “pthreat” representing a local threat affecting survival of nearby cells (i.e., cells in a radius of 1); patches also have resources, and those resources diffuse themselves around; quality of resources degrades over time. Cluster of cells are initialized considering variable densities that may affect cells survival.

iv. Collectives

Different types of agents are characterized by specific behaviors and properties:

1. Cells are defined by
   a. a set of properties:
      1. Surface: this is the surface each cell exposes to the environment, affecting both the amount of resources each cell may have access to, and the vulnerability to environmental threat.
      2. Anoikis: this is an individual property modeling the tendency of cells to commit suicide when not in close proximity to other cells.
      3. Nlinks: this is the actual number of links to other cells each cell has; it is defined by a simple cut-off spatial distance.
      4. Vulnerability: this is an individual property of cells modeling how vulnerable they are to environmental threat; it is a nonlinear function (more precisely, a sigmoid function) of each cell surface.
      5. Energy: this is the energy budget each cell is equipped with at the very beginning of each run of the simulation and it is updated over time (it increases when a cell reaches a resource “food”, and it decreases when a cell is affected by a patch threat).
      6. Metabolic: this is an individual property modeling the metabolic rate of each cell.
   b. a set of behaviors:

i. Eat: cells get energy from “food” resources over time, accessing resources available locally (i.e., on the patch where a specific cell is).

2. Patches are defined by
    a. a set of properties:
        i. Threat: this is an individual property modeling the action of local threat negatively affecting the energy of cells on the patch.
        ii. Food: this is an individual property modeling the current availability of resources on the patch. It varies over time decreasing because of “food” degradation and increasing because “food” diffusion through the environment.
    b. a set of behaviors:
        i. Diffuse-food: patches diffuse resources.
        ii. Threat: patches affect cell energy.

3. Process overview and scheduling

- *Questions*: Who (i.e., what entity) does what, and in what order? When are state variables updated? How is time modeled, as discrete steps or as a continuum over which both continuous processes and discrete events can occur? Except for very simple schedules, one should use pseudo-code to describe the schedule in every detail, so that the model can be re-implemented from this code. Ideally, the pseudo-code corresponds fully to the actual code used in the program implementing the ABM.
- A particular environment is initialized; a cluster of cells with a specific density is initialized. The particular initial spatial configuration of the cluster (i.e., the network topology) is saved. A typical run of the model starts with the degradation of local resources and the update of cells properties. Then, cells check whether there is a local threat affecting their energy or not (threat affects cells in accordance with the actual surface they expose to the environment), and eventually check whether they die or not: cells die as soon as they reach the critical energetic threshold of zero or because of anoikis (in case there are no other cells in a given distance). Then, cells have access to environmental resources in accordance with the actual surface they expose to the environment. After a fixed period of time, new resources are available to cells. The run ends when the model reaches a given time t or when there are no cells anymore. At the end of the run information about state variables (N, density, availability of resources, patches threat, median energy of cells, median availability of resources, median metabolic rate of cells) is saved.

Moreover, the particular spatial configuration of the cluster (i.e., the network topology) resulting is saved.

4. Design concepts

- Basic principles: Which general concepts, theories, hypotheses, or modeling approaches are underlying the model's design?
- The model gets inspiration from the behavior of circulating tumor cell clusters. This model investigates a specific behavior of cluster cells exhibited when cluster cells move around the organism and before they disseminated at a new location. Specifically, we aim to better understand patterns of environmental conditions and cluster characteristics minimizing (or maximizing) cells survival during transit from primary tumor sites to metastatic seeding sites. Moreover, we aim to investigate the effects of specific cluster configurations (i.e., network topology) potentially affecting the ability of cells in clusters to survive in risky environments and the stability of the clusters. The model is potentially useful for different purposes:
    1. it can be used to identify crucial elements and factors affecting clusters' behavior;
    2. it can be used to test theoretical hypotheses about the role and relevance of those elements and factors by the manipulation of different parameters of the model;
    3. it can be used to generate a big amount of in-silico data that can be used to run analyses that otherwise it would be difficult to run with biological data (both in vivo and in vitro) because of many different reasons (ethical, temporal, spatial);
    4. finally, it can allow for fruitful interactions of between the model and experiments in the lab.

- ***Emergence***: What key results or outputs of the model are modeled as emerging from the adaptive traits, or behaviors, of individuals?
- Survival of cells depends on different factors (i.e., individual properties and environmental conditions) and results from the interactions between cells and between cells and the environment itself. Nonlinear dynamics are assumed to play a role in affecting the ability of cells to have access to resources and to interact with other cells and the environment. Finally, biological principles (e.g., anoikis) are assumed to play a relevant role, as well.

- ***Adaptation***: What adaptive traits do the individuals have? What rules do they have for making decisions or changing behavior in response to changes in themselves or their environment?
- In this model we do not assume adaptive traits.

- ***Objectives***: If adaptive traits explicitly act to increase some measure of the individual's success at meeting some objective, what exactly is that objective and how is it measured? When individuals make decisions by ranking alternatives, what criteria do they use?
- Cell agents are equipped with the implicit objective to survive. Patches are equipped with the implicit objective to undermine survival of cells.

- ***Learning***: Many individuals or agents (but also organizations and institutions) change their adaptive traits over time as a consequence of their experience? If so, how?
- Agents are not able to learn. They are assumed to be simple agents not able to do it.

- ***Prediction***: Prediction is fundamental to successful decision-making; if an agent's adaptive traits or learning procedures are based on estimating future consequences of decisions, how do agents predict the future conditions (either environmental or internal) they will experience? If appropriate, what internal models are agents assumed to use to estimate future conditions or consequences of their decisions? What tacit or hidden predictions are implied in these internal model assumptions?
- Agents of this model can not make any predictions.

- ***Sensing***: What internal and environmental state variables are individuals assumed to sense and consider in their decisions? What state variables of which other individuals and entities can an individual perceive; for example, signals that another individual may intentionally or unintentionally send?
- Cell agents perceive other cell agents and patch agents.

- ***Interaction***: What kinds of interactions among agents are assumed? Are there direct interactions in which individuals encounter and affect others, or are interactions indirect, e.g., via competition for a mediating resource? If the interactions involve communication, how are such communications represented?
- We assume that the model models both direct and indirect interactions. Direct interactions result from the actual interaction between agents, more

specifically cell-cell interactions and cell-patch interactions. Indirect interactions result from the actual cluster configuration and the specific environmental conditions.

- ***Stochasticity***: What processes are modeled by assuming they are random or partly random? Is stochasticity used, for example, to reproduce variability in processes for which it is unimportant to model the actual causes of the variability? Is it used to cause model events or behaviors to occur with a specified frequency?
- Stochasticity is used in different processes and to initialize all agents' properties.

- ***Collectives***: Do the individuals form or belong to aggregations that affect, and are affected by, the individuals? Such collectives can be an important intermediate level of organization in an ABM; examples include social groups, fish schools and bird flocks, and human networks and organizations. How are collectives represented? Is a particular collective an emergent property of the individuals, such as a flock of birds that assembles as a result of individual behaviors, or is the collective simply a definition by the modeler, such as the set of individuals with certain properties, defined as a separate *kind* of entity with its own state variables and traits?
- Collectives may affect the behavior of the system. Specific cluster configurations may strongly affect the survival of cells in combination with some particular local environmental conditions.

- ***Observation***: What data are collected from the ABM for testing, understanding, and analyzing it, and how and when are they collected? Are all output data freely used, or are only certain data sampled and used, to imitate what can be observed in an empirical study ("Virtual Ecologist" approach; Zurell et al., 2010)?
- The particular initial spatial configuration of the cluster (i.e., the network topology) is saved. Information about state variables (N, density, availability of resources, patches threat, median energy of cells, median availability of resources, median metabolic rate of cells) is saved at the end of each run. Moreover, the particular spatial configuration of the cluster (i.e., the network topology) resulting is saved at the end of each run.

5. Initialization

- *Questions*: What is the initial state of the model world, i.e., at time $t = 0$ of a simulation run? In detail, how many entities of what type are there initially,

and what are the exact values of their state variables (or how were they set stochastically)? Is initialization always the same, or is it allowed to vary among simulations? Are the initial values chosen arbitrarily or based on data? References to those data should be provided.

- A particular environment is initialized; a cluster of cells with a specific density is initialized. All agents properties are stochastically initialized.

6. **Input data**
    - *Question*: Does the model use input from external sources such as data files or other models to represent processes that change over time?
    - The model does not use input from external sources.

7. **Submodels**
    - *Questions*: What, in detail, are the submodels that represent the processes listed in 'Process overview and scheduling'? What are the model parameters, their dimensions, and reference values? How were submodels designed or chosen, and how were they parameterized and then tested?
    - The model consists of several submodels:
        i. Environment initialization: availability of resources; local threat.
        ii. Cell initialization.
        iii. Cell behavior: get resources; interact with the environment (i.e., check effects of local threat); check possible death (if reached critical threshold).
        iv. Update in availability of resources: new resources are available to cells at a given regular interval.

Table 1. A list of the model's parameters, value range and initial values.

**Model Parameter**	**Value range**	**Initial value (mean, sd)**
Anoikis	{0,1}	normal(0.5,0.02)
Nlinks	{0,1}	# of other cells in-radius 1
Surface	{0,1}	1 / (nlinks + 1)
Vulnerability	{0,1}	see Eq. 2 in main text
Energy	{0,1}	normal(0.5,0.02)
Metabolic	{0,1}	uniform(0,1)
Threat	{0,1}	normal({0.1,0.9},0.02)
N	{20,100}	{20,40,80,100}
Density	{low, high}	{low, medium, high}
Resources_availability	{0,1}	{0,1}
Resource	{0,1}	see Eq. 4 in main text
Resource_degradation	{0,1}	0.02

2. Explanations for the equations used in the model.

Equations are based on a combination of mathematical and biological considerations as well as common use in similar other modelling approaches (Cramer 2004).

Eq. 1

$$surface_{x_t} = \frac{1}{links_{x_t} + 1}$$

Equation 1 models cell surface as the portion of the cell accessible to both resources and threats, as a function of the number of other cells the current cell is linked to (i.e., in contact with).

Eq. 2

$$vulnerability_{x_t} = \frac{1}{\left(1 + e^{-\left(\left(\left(surface_{x_t} * a\right) - 1\right) * b\right)}\right)}$$

Equation 2 models “vulnerability” as a logistic function of its surface. A logistic function nicely models the nonlinear dynamics resulting from the transition from being an isolated cell where the entire surface is exposed to environmental nutrients and threats to a situation where a cell is in contact with many other cells and therefore the proportion of its surface exposed to the surrounding environment is very limited. The logistic function is a good representation of the transition phase from being an isolated cell to be a cell linked to (i.e., in contact with) other cells, taking into consideration the fact that there is probably a critical number of neighbours driving this transition.

Eq. 3

$$energy_{x_{t+1}} = energy_{x_t} + \left(resource_{y_{t+1}} * metabolic_x * surface_{x_{t+1}}\right) - \left(threat_y * vulnerability_{x_{t+1}}\right)$$

Equation 3 models the cell energy dynamics at each time step of the model. It considers some crucial aspects of this process, such as i) the initial energy level, ii) the energy intake resulting from the exposure to surrounding resources mediated by the available cell

surface and its individual metabolic rate, and finally iii) the energy consumption resulting from the cell surface exposure to surrounding threats.

Eq. 4

$$resource_{y_{t+1}} = resource_{y_t} - \left(resource_{y_t} * d\right) + f(resource_{y_t}, resource_{neighbors_t}, k)$$

Equation 4 models the dynamics of resources at each time step of the model as the linear combination of i) the initial amount of resources decreased by ii) the resources degradation parameter and iii) increased by the diffusion process driving the flow of resources through the environment.

3. Results “at the equilibrium”

In this section the complete collection of results obtained running the model(s) is presented.
Figures show the values of the Survival Ratio (SR) and the Stability Index (SI) calculated at the equilibrium, i.e., at the end of each simulation (for details about SR and SI, see the main text and Figure 3).
Here, we present all the results in the same order adopted in the main text. For each specific combination of parameters, plots of Survival Ratio are presented first, followed by Figures of Stability Index.
Results averaged 1000 replicates of the same combinations of parameters.

Figure S1. The impact of density and size on cluster resilience and stability in optimal environments

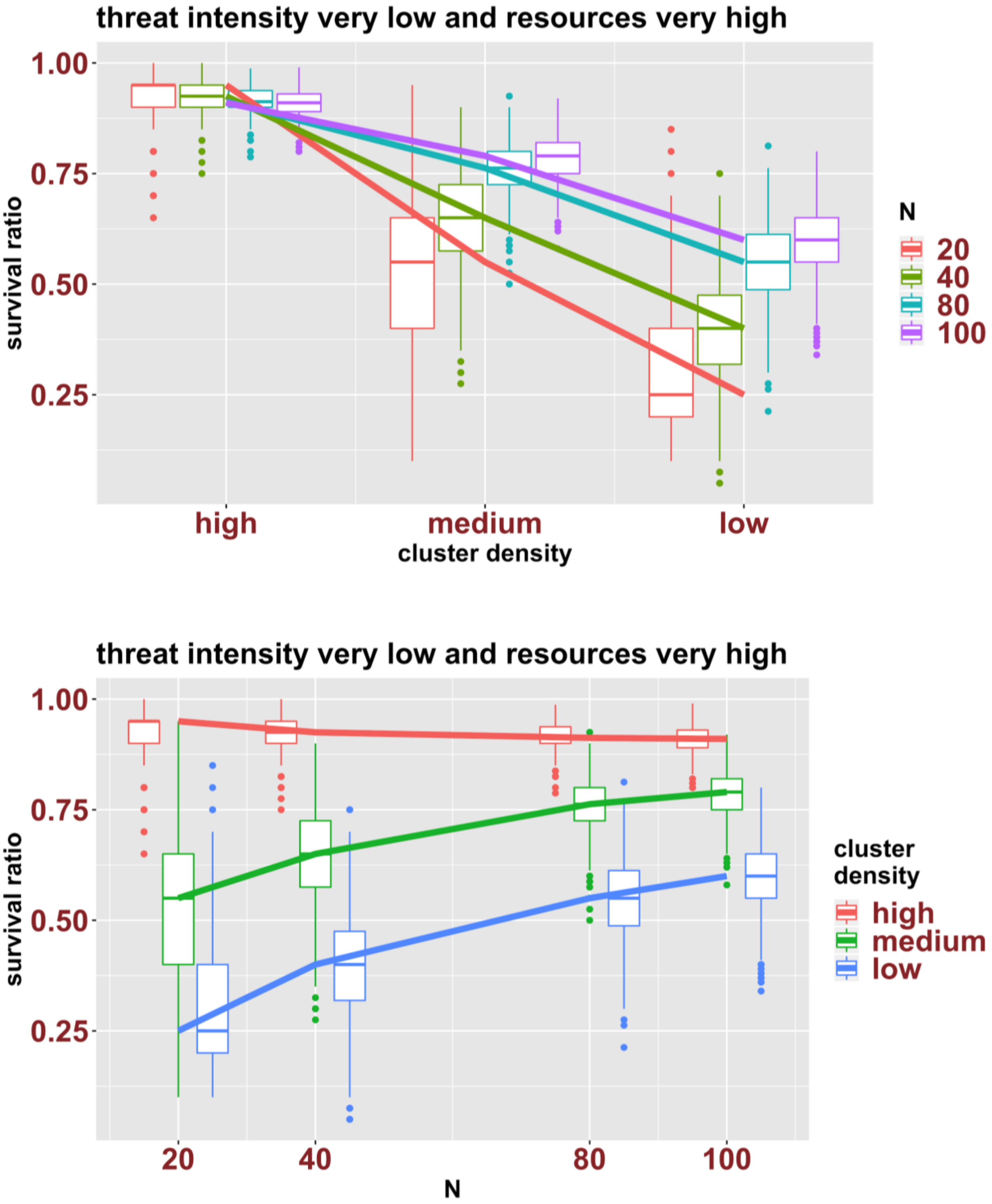

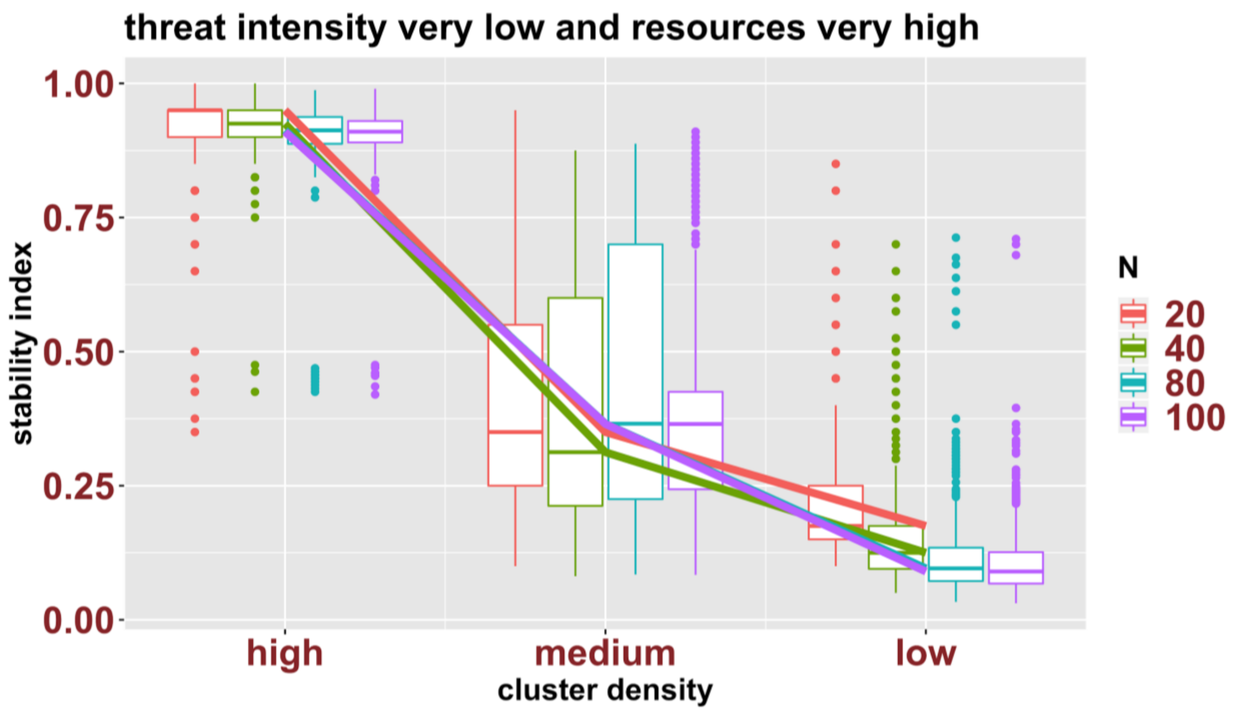
threat intensity very low and resources very high
stability index
1.00
0.75
0.50
0.25
0.00
high
medium
low
cluster density
N
20
40
80
100

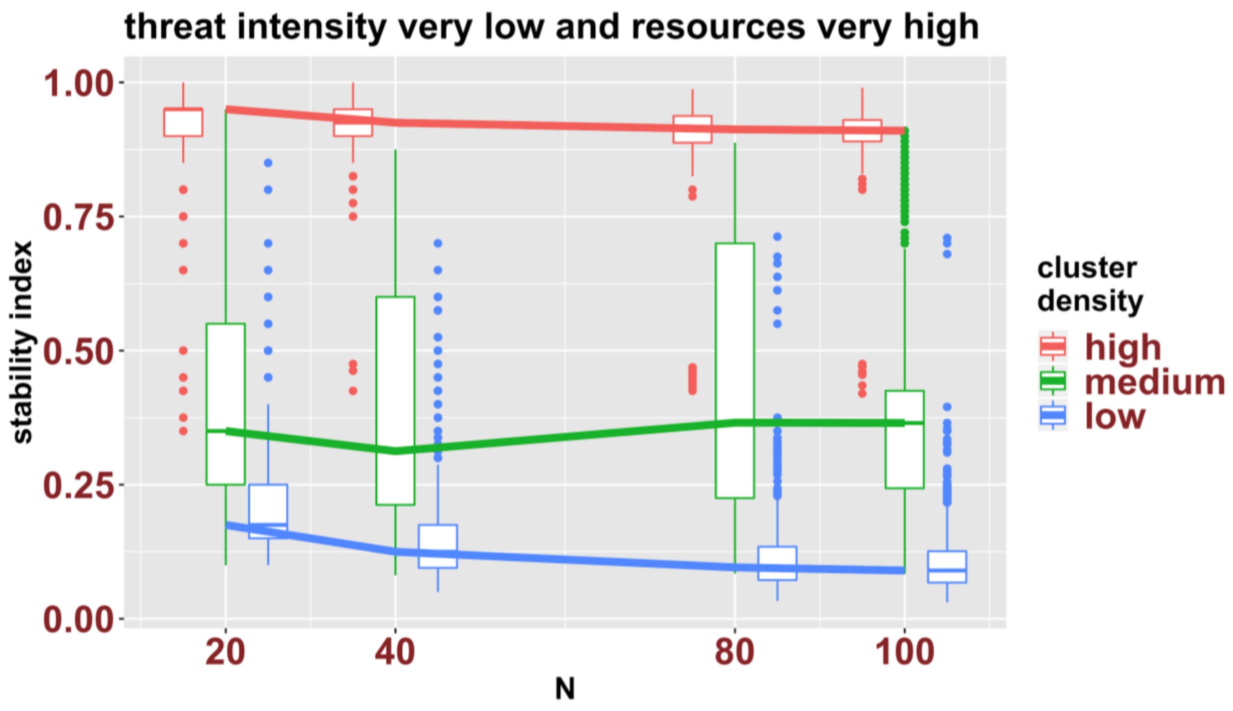
threat intensity very low and resources very high
stability index
1.00
0.75
0.50
0.25
0.00
20
40
80
100
N
cluster density
high
medium
low

Figure S2. The effect of resource availability on cluster persistence and stability

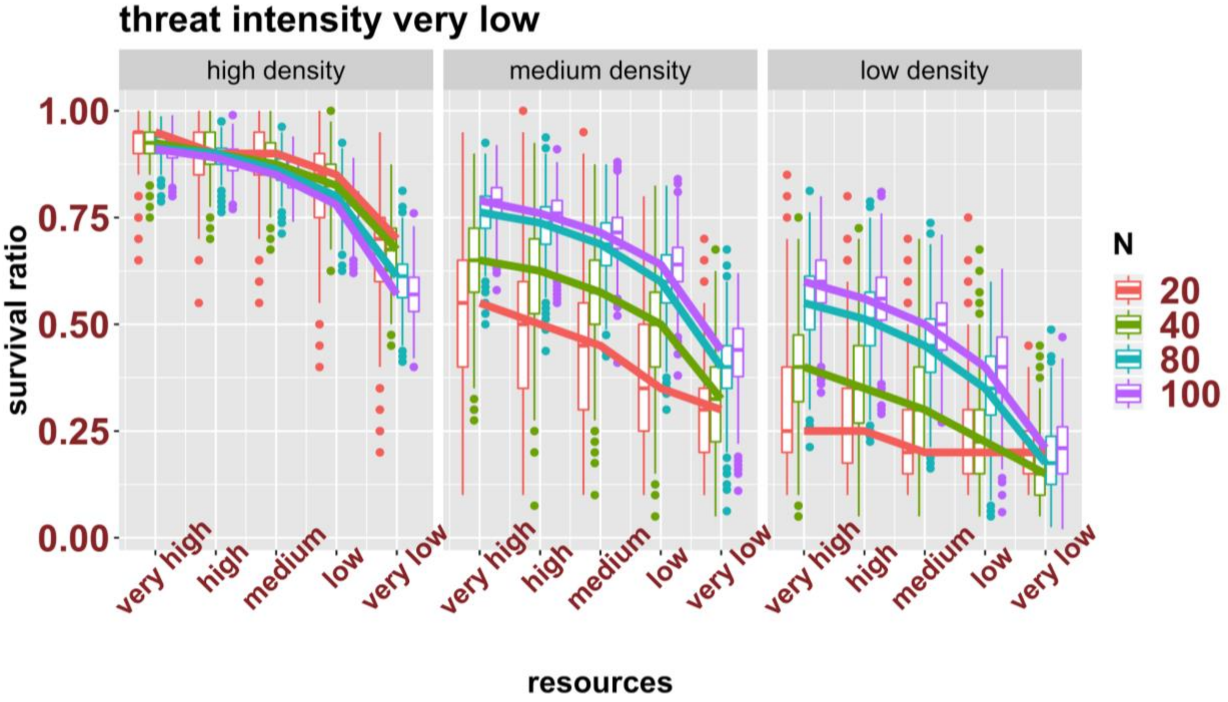

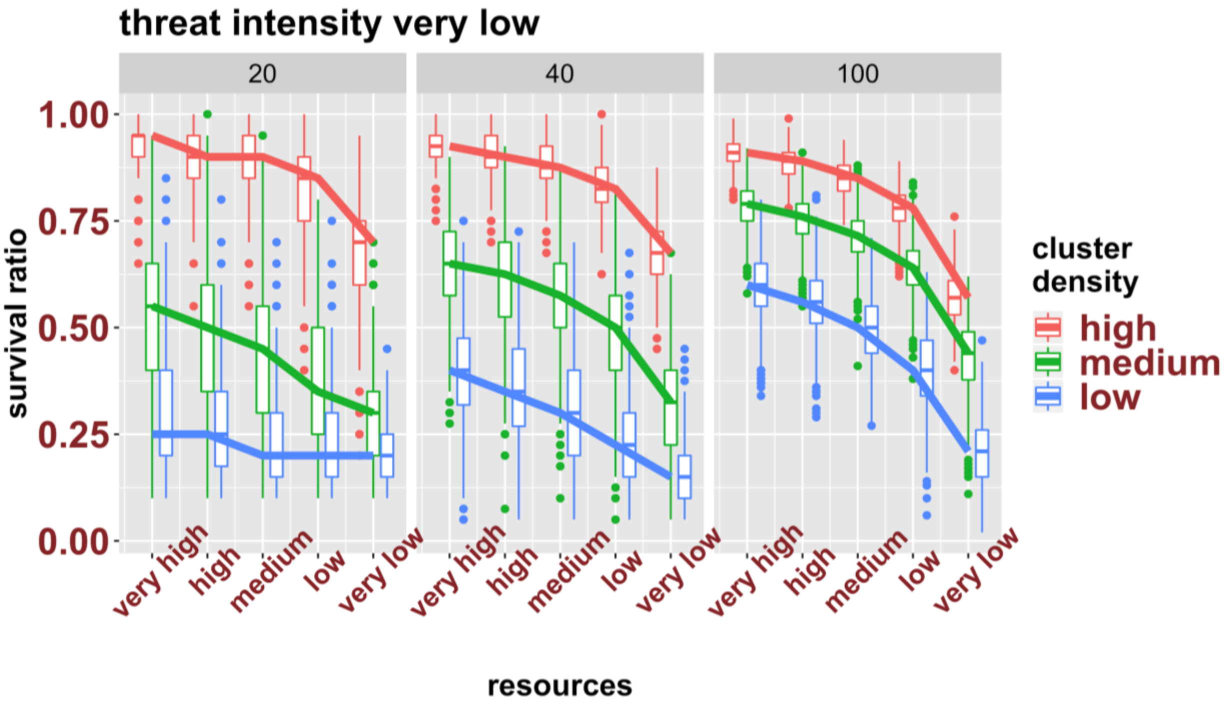

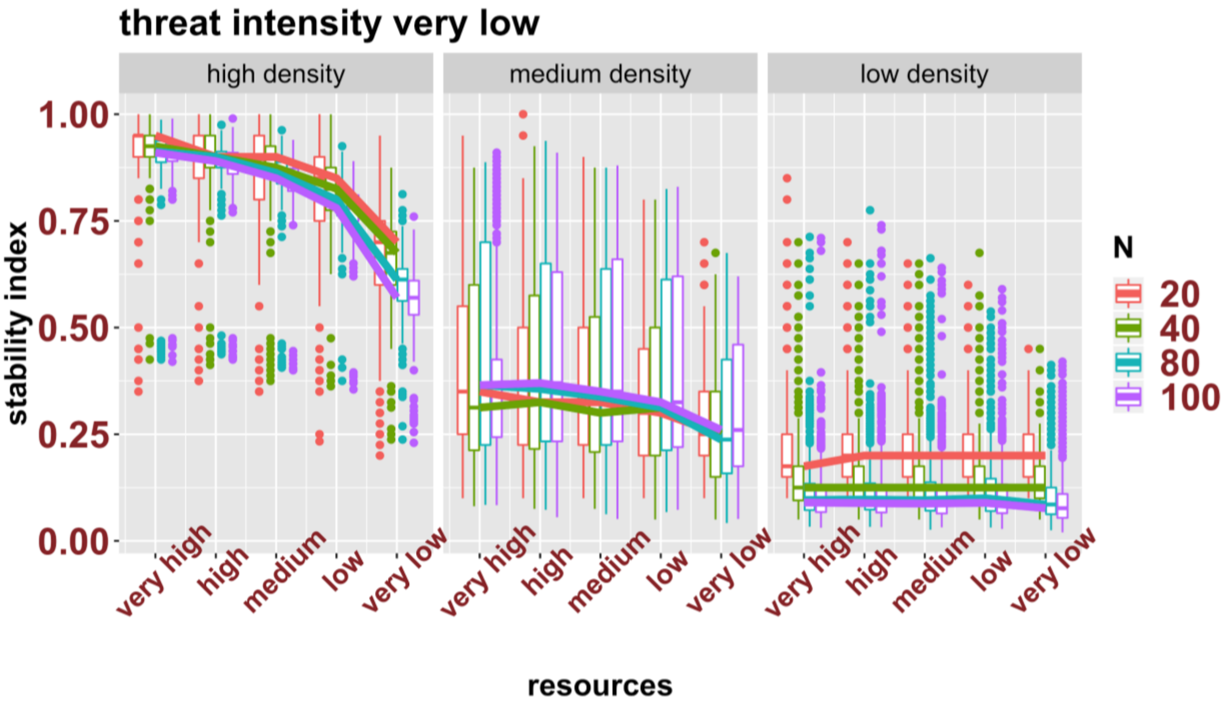

threat intensity very low
high density
medium density
low density
stability index
1.00
0.75
0.50
0.25
0.00
very high
high
medium
low
very low
resources
N
20
40
80
100

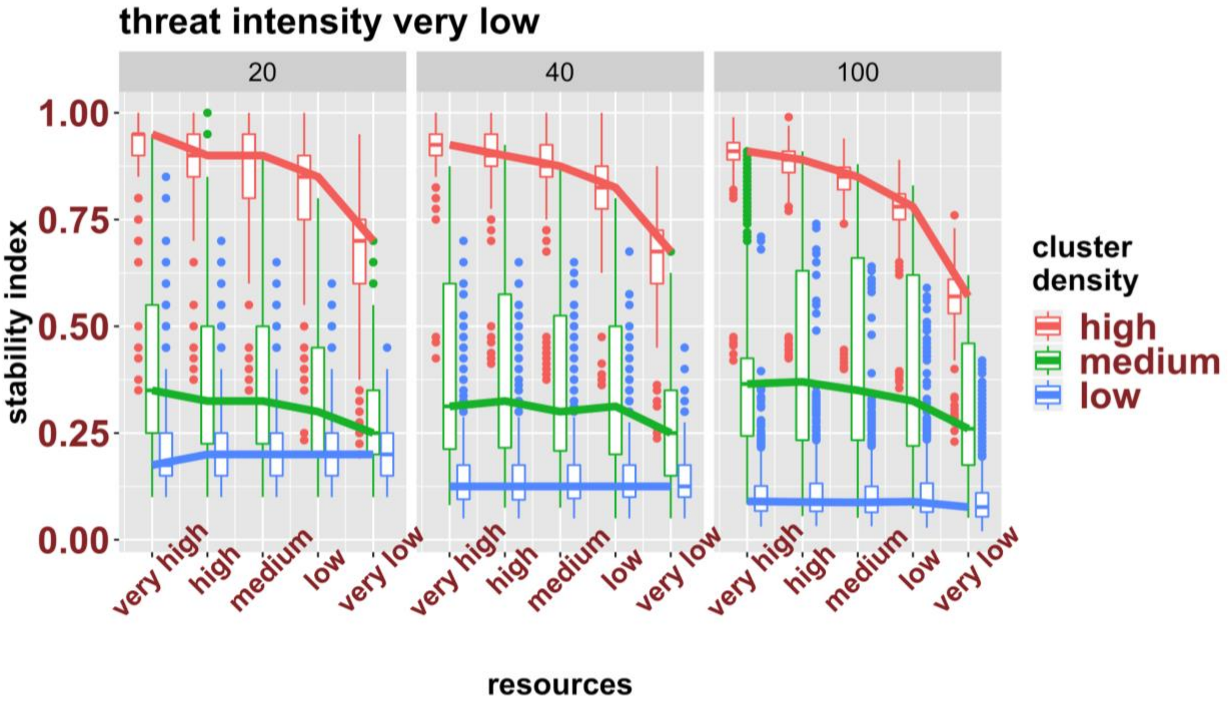

threat intensity very low
20
40
100
stability index
1.00
0.75
0.50
0.25
0.00
very high
high
medium
low
very low
resources
cluster density
high
medium
low

Figure S3. The effect of environmental threats on cluster persistence and stability

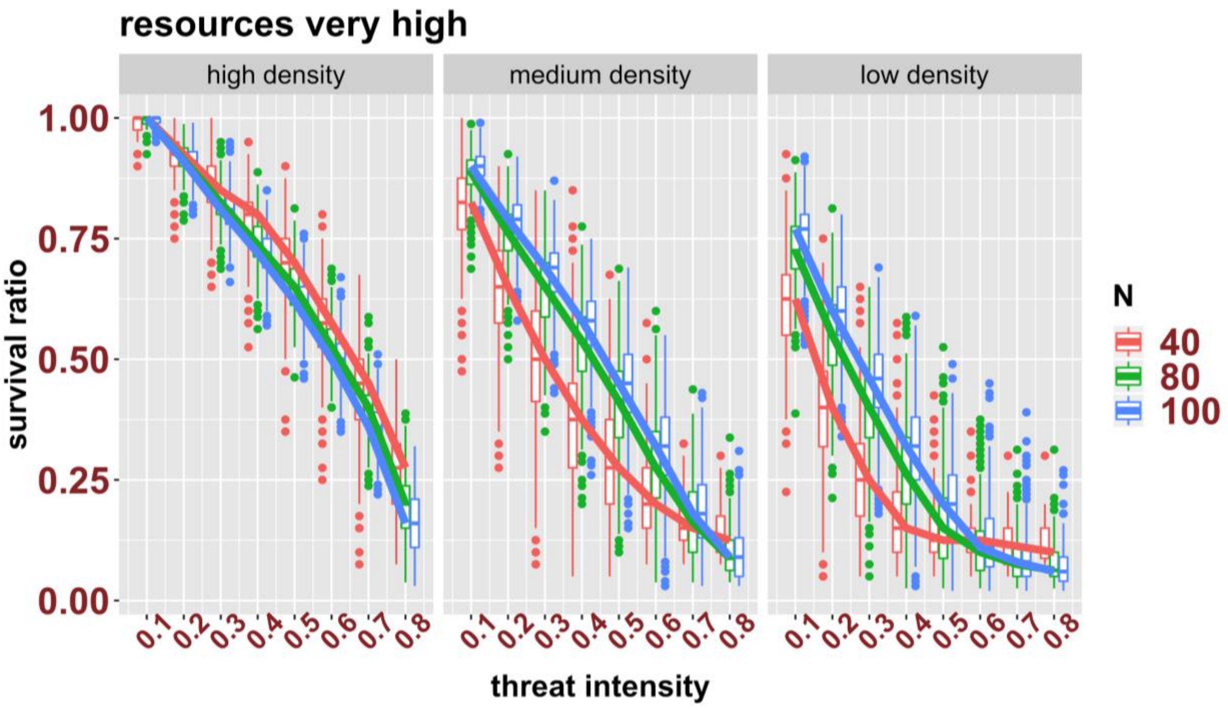

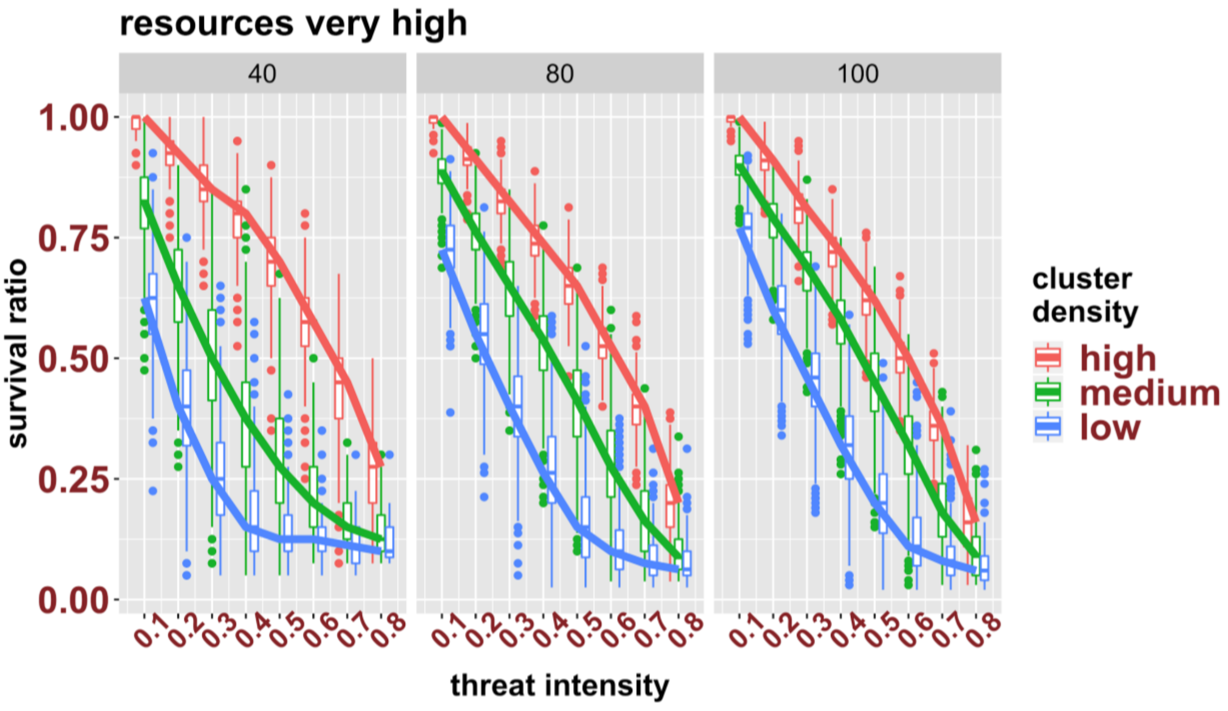

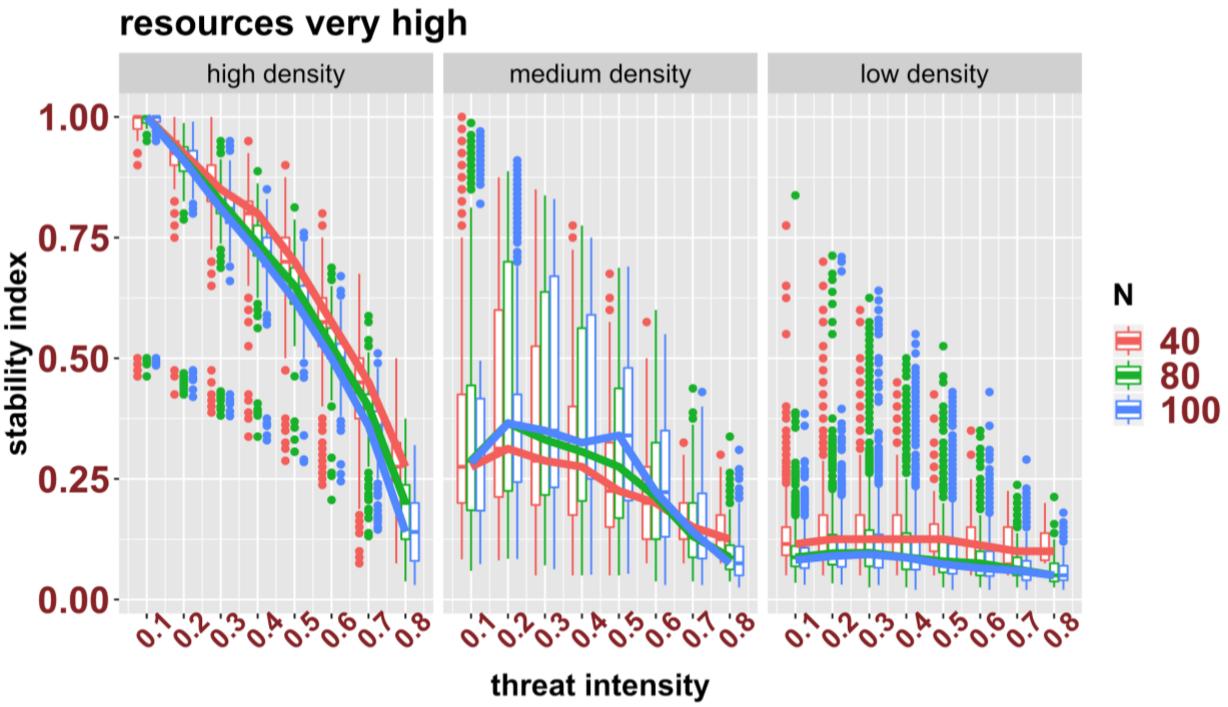

resources very high
high density
medium density
low density
stability index
1.00
0.75
0.50
0.25
0.00
0.1
0.2
0.3
0.4
0.5
0.6
0.7
0.8
threat intensity
N
40
80
100

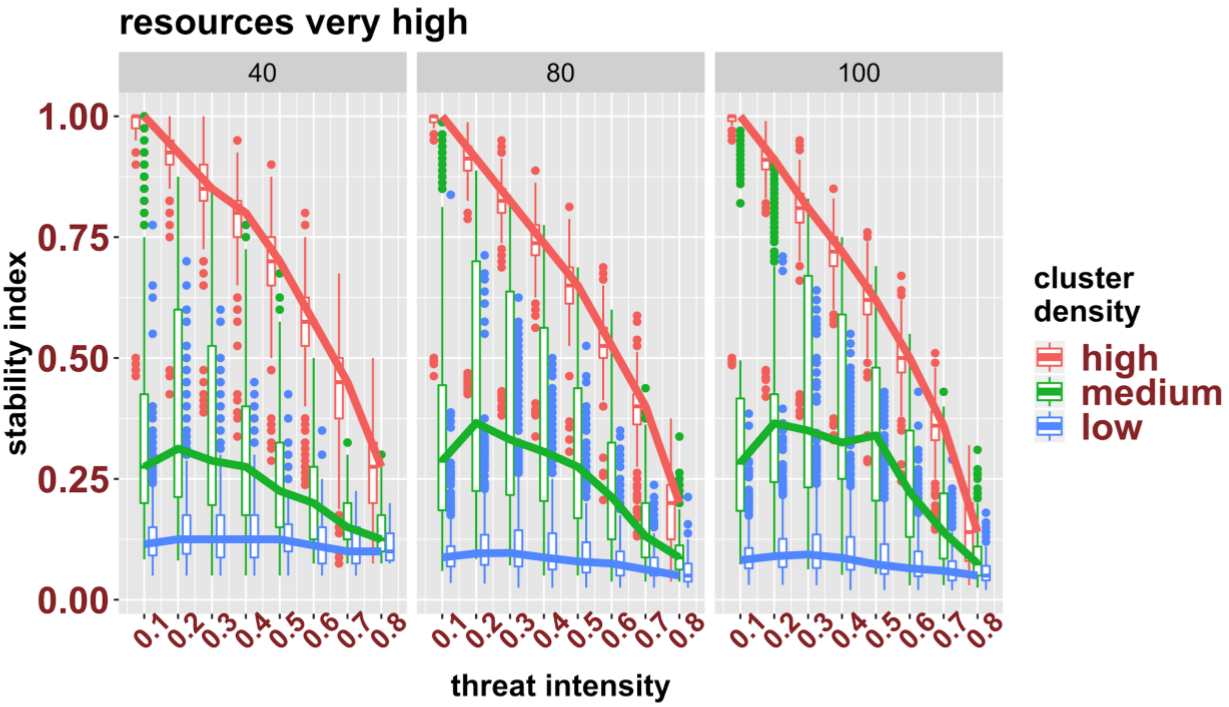

resources very high
40
80
100
stability index
1.00
0.75
0.50
0.25
0.00
0.1
0.2
0.3
0.4
0.5
0.6
0.7
0.8
threat intensity
cluster density
high
medium
low

Figure S4. The combined effect of environmental threats and resource availability on cluster survival

A. ***Survival Ratio***

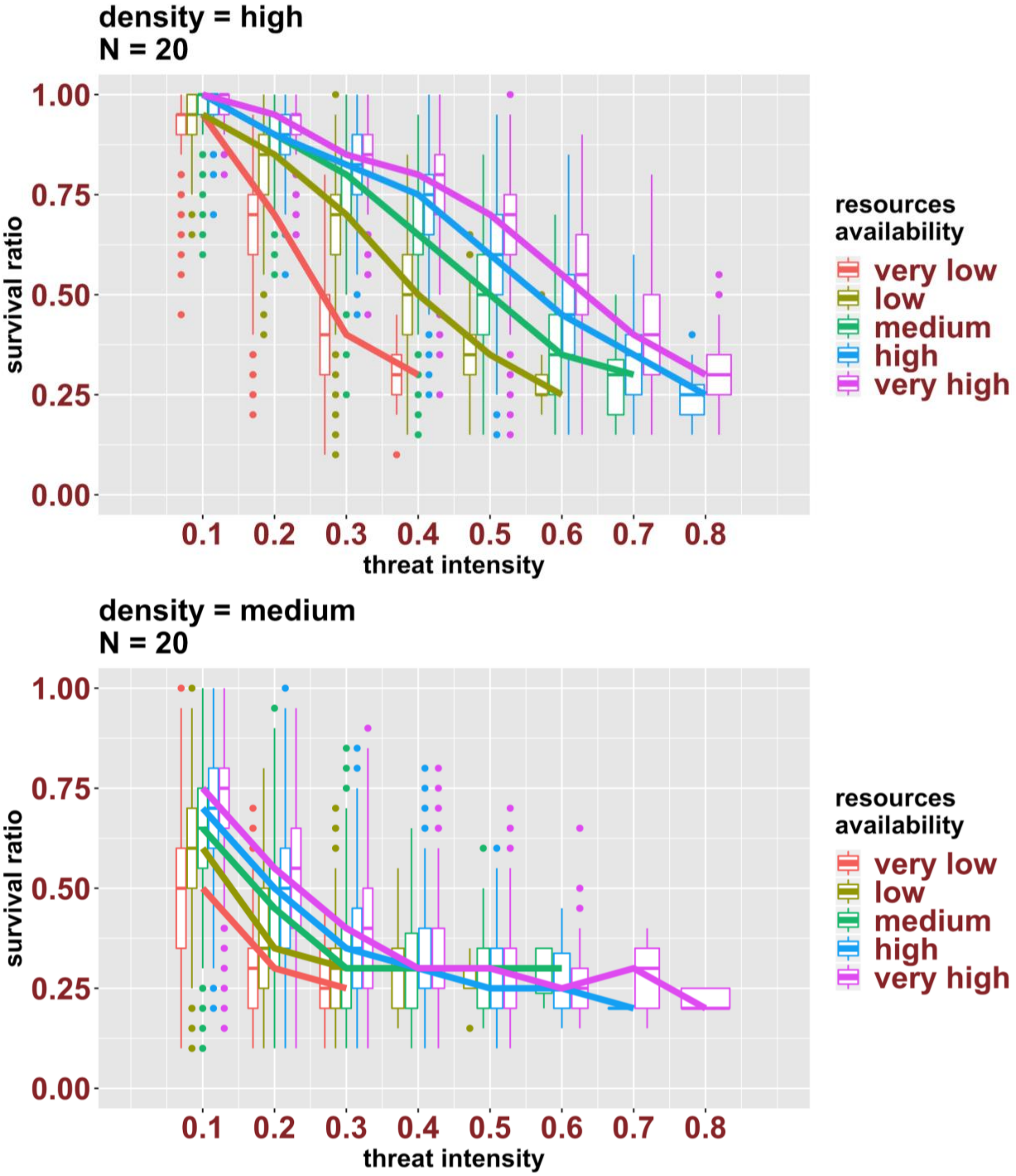

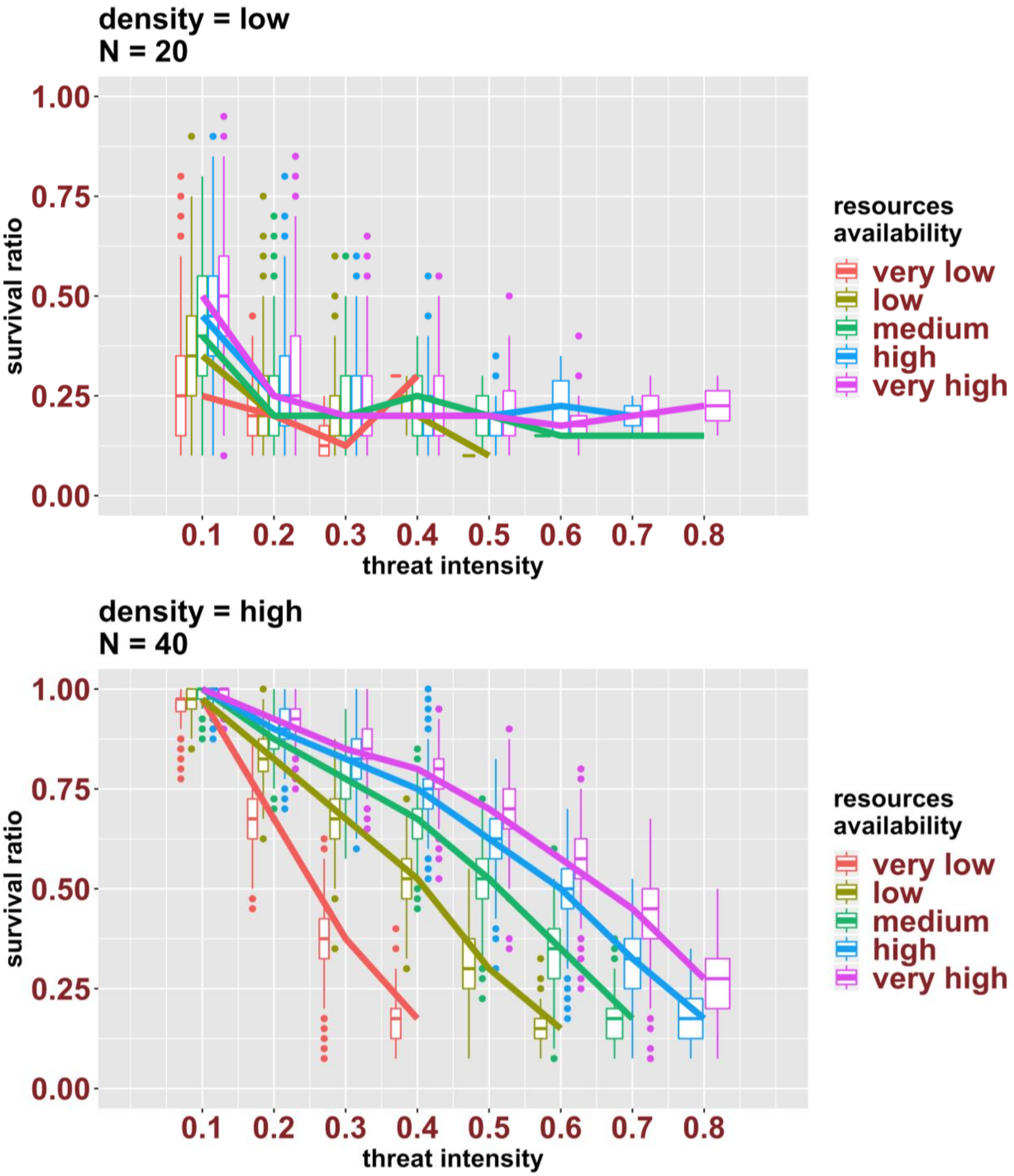

density = low
N = 20
survival ratio
1.00
0.75
0.50
0.25
0.00
0.1
0.2
0.3
0.4
0.5
0.6
0.7
0.8
threat intensity
resources
availability
very low
low
medium
high
very high
density = high
N = 40
survival ratio
1.00
0.75
0.50
0.25
0.00
0.1
0.2
0.3
0.4
0.5
0.6
0.7
0.8
threat intensity
resources
availability
very low
low
medium
high
very high

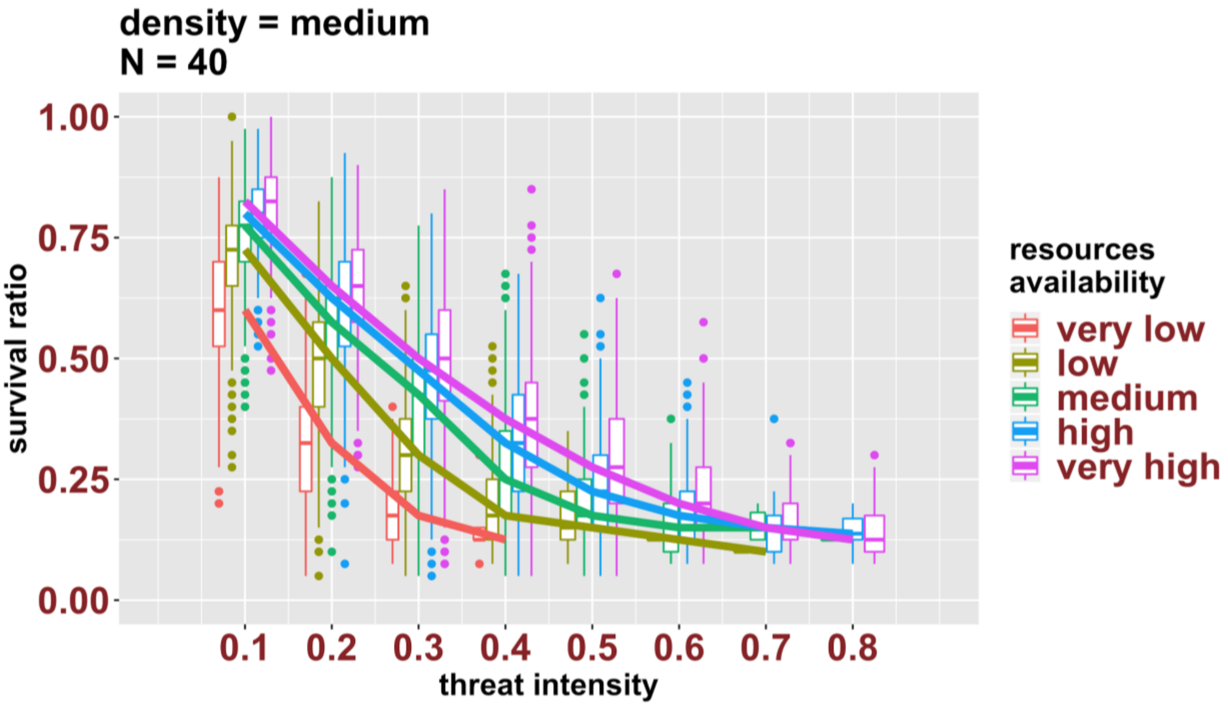
density = medium
N = 40
survival ratio
1.00
0.75
0.50
0.25
0.00
0.1
0.2
0.3
0.4
0.5
0.6
0.7
0.8
threat intensity
resources
availability
very low
low
medium
high
very high

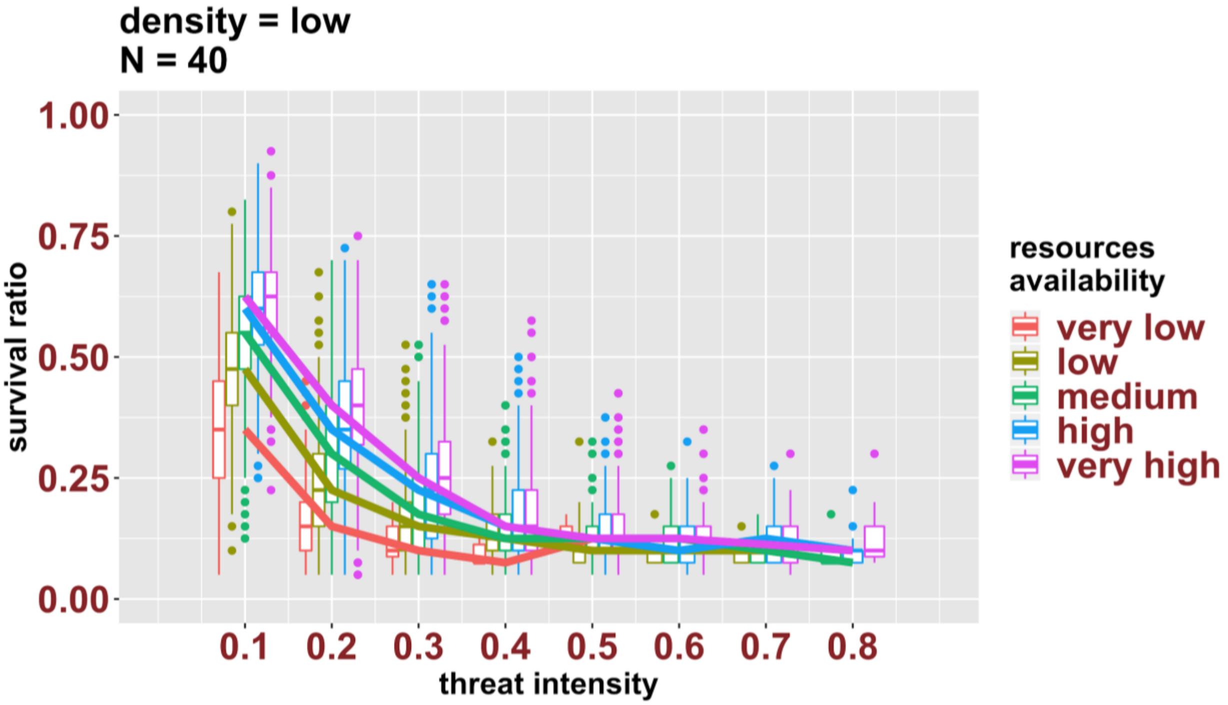
density = low
N = 40
survival ratio
1.00
0.75
0.50
0.25
0.00
0.1
0.2
0.3
0.4
0.5
0.6
0.7
0.8
threat intensity
resources
availability
very low
low
medium
high
very high

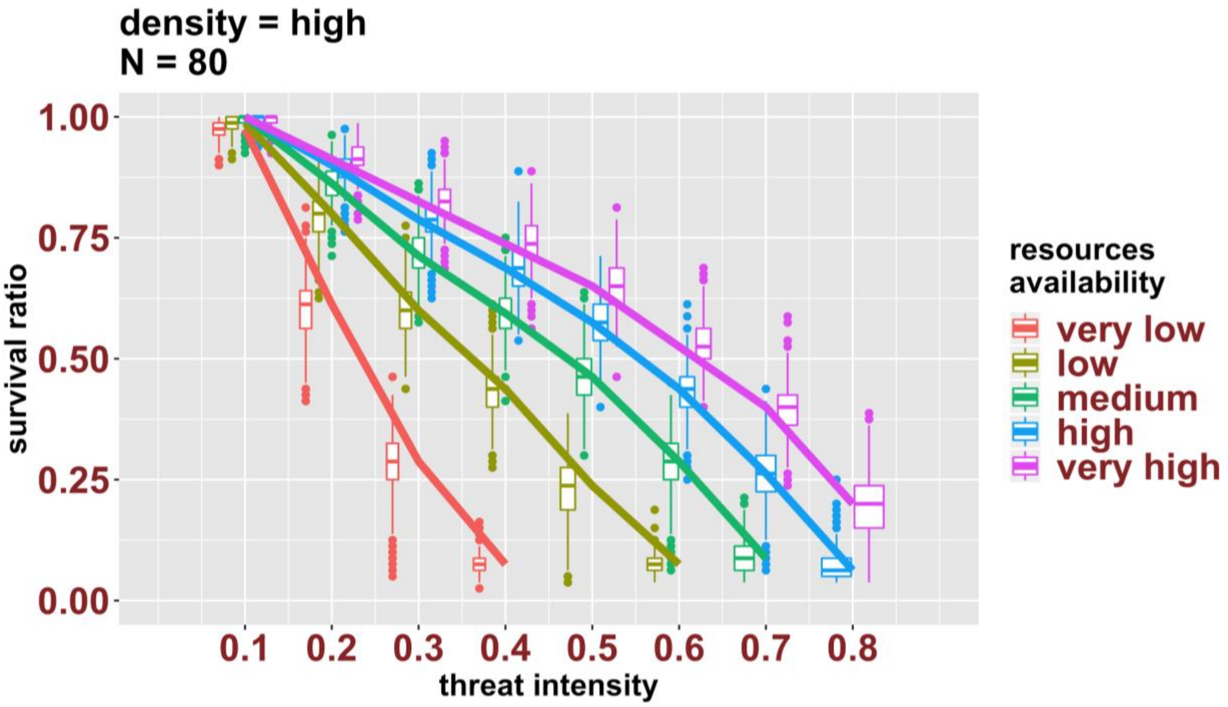
density = high
N = 80
1.00
0.75
0.50
0.25
0.00
survival ratio
0.1
0.2
0.3
0.4
0.5
0.6
0.7
0.8
threat intensity
resources
availability
very low
low
medium
high
very high

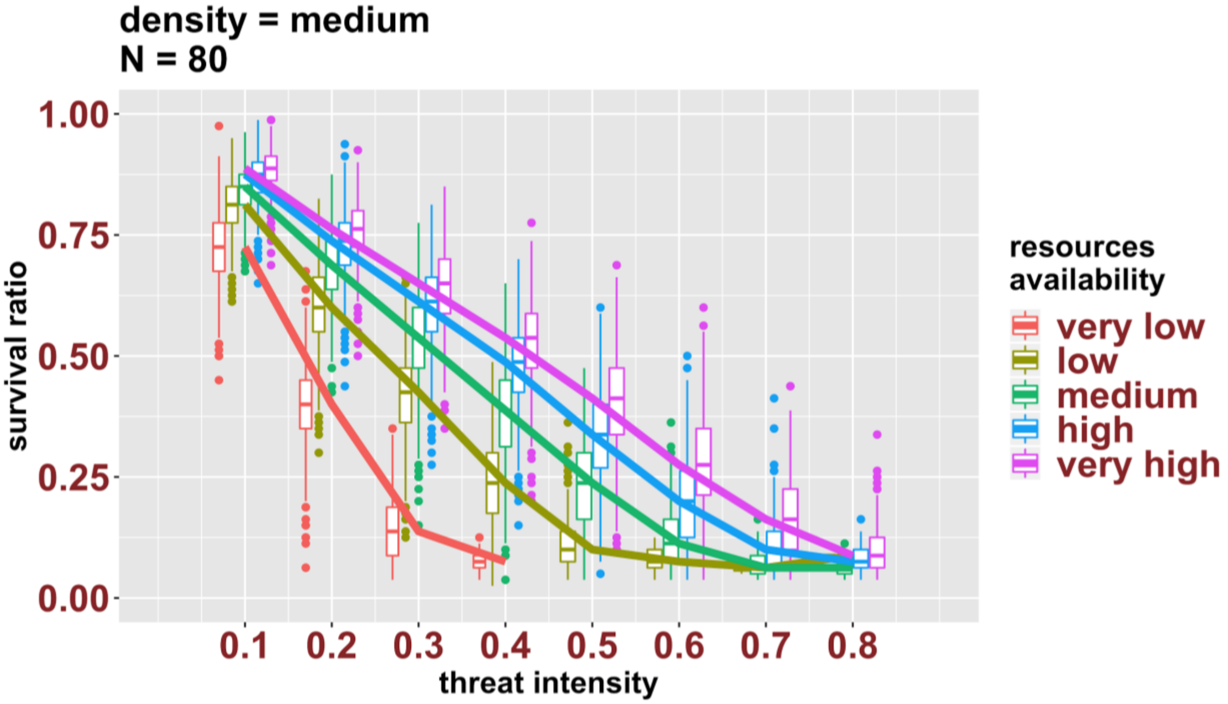
density = medium
N = 80
1.00
0.75
0.50
0.25
0.00
survival ratio
0.1
0.2
0.3
0.4
0.5
0.6
0.7
0.8
threat intensity
resources
availability
very low
low
medium
high
very high

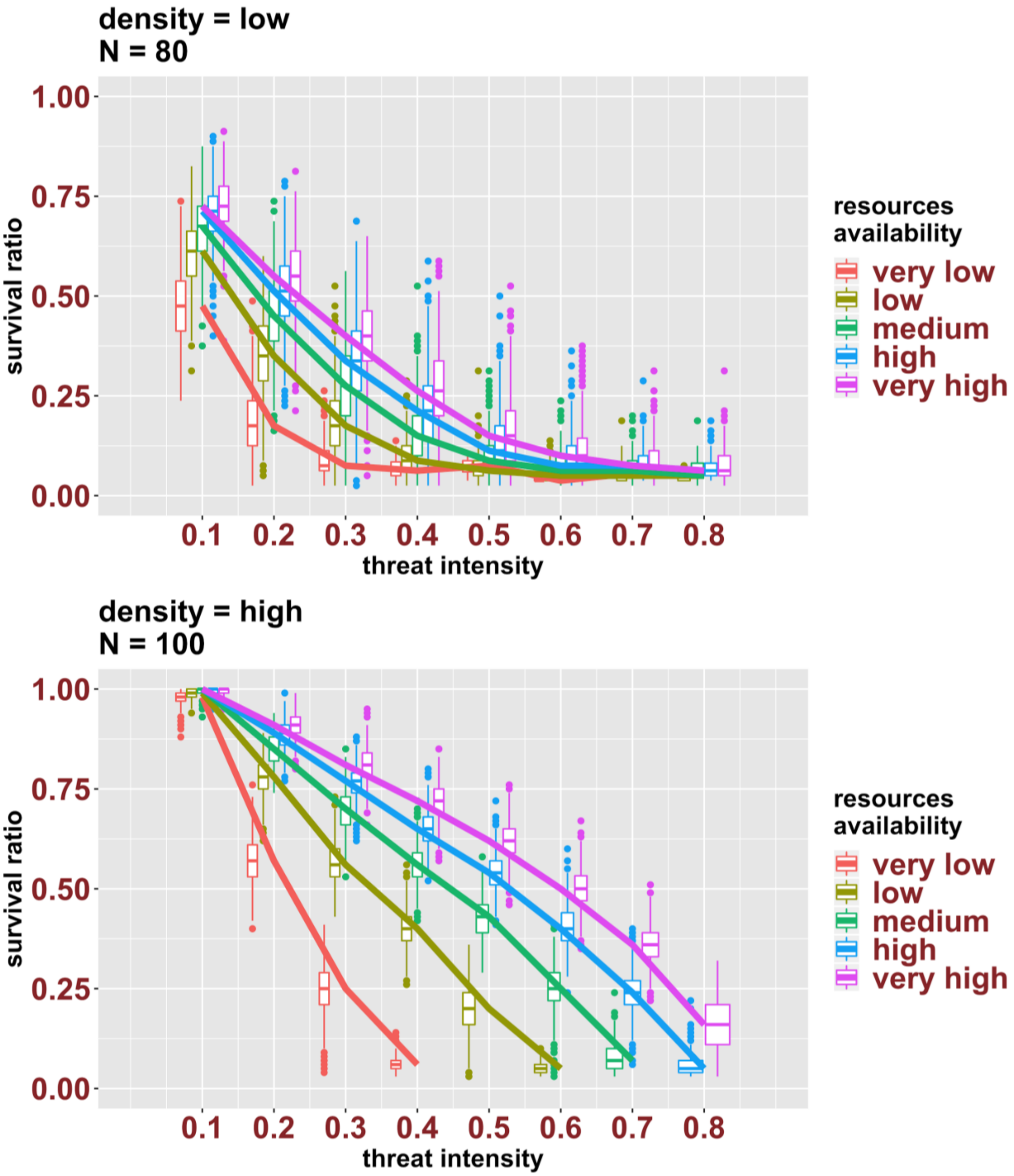
density = low
N = 80
survival ratio
1.00
0.75
0.50
0.25
0.00
0.1
0.2
0.3
0.4
0.5
0.6
0.7
0.8
threat intensity
resources availability
very low
low
medium
high
very high
density = high
N = 100
survival ratio
1.00
0.75
0.50
0.25
0.00
0.1
0.2
0.3
0.4
0.5
0.6
0.7
0.8
threat intensity
resources availability
very low
low
medium
high
very high

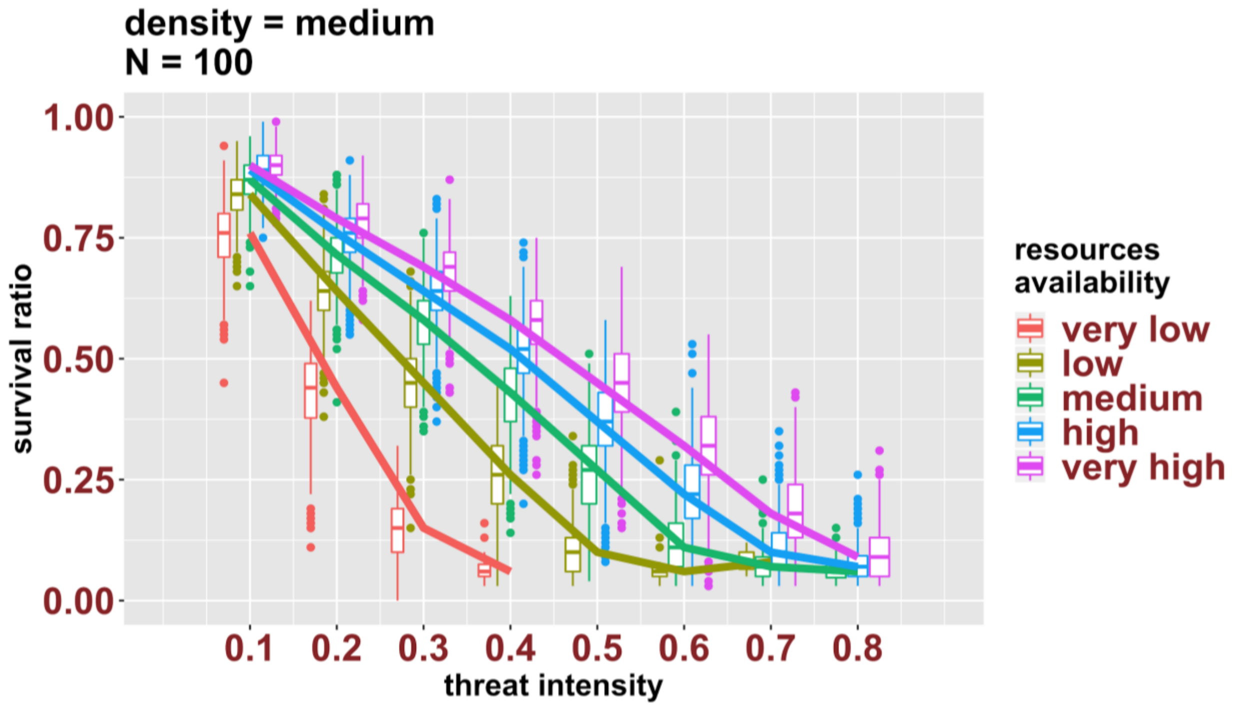
density = medium
N = 100
survival ratio
1.00
0.75
0.50
0.25
0.00
0.1
0.2
0.3
0.4
0.5
0.6
0.7
0.8
threat intensity
resources availability
very low
low
medium
high
very high

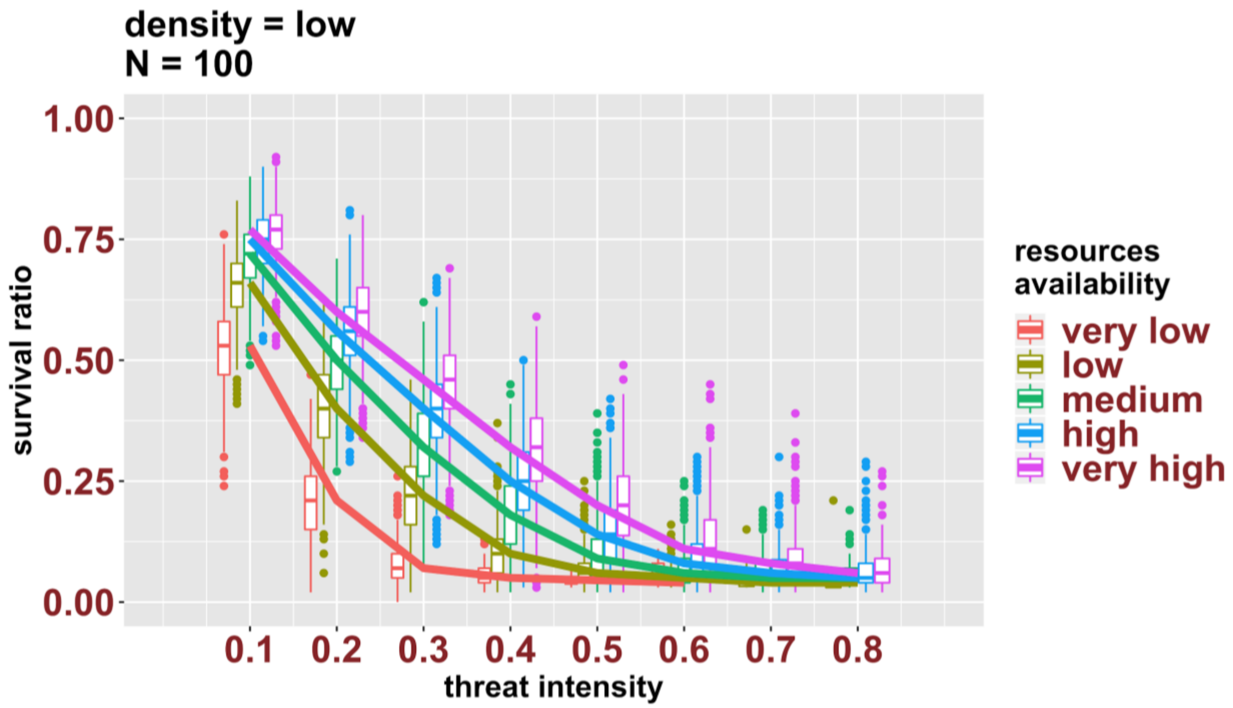
density = low
N = 100
survival ratio
1.00
0.75
0.50
0.25
0.00
0.1
0.2
0.3
0.4
0.5
0.6
0.7
0.8
threat intensity
resources availability
very low
low
medium
high
very high

A. *Stability Index*

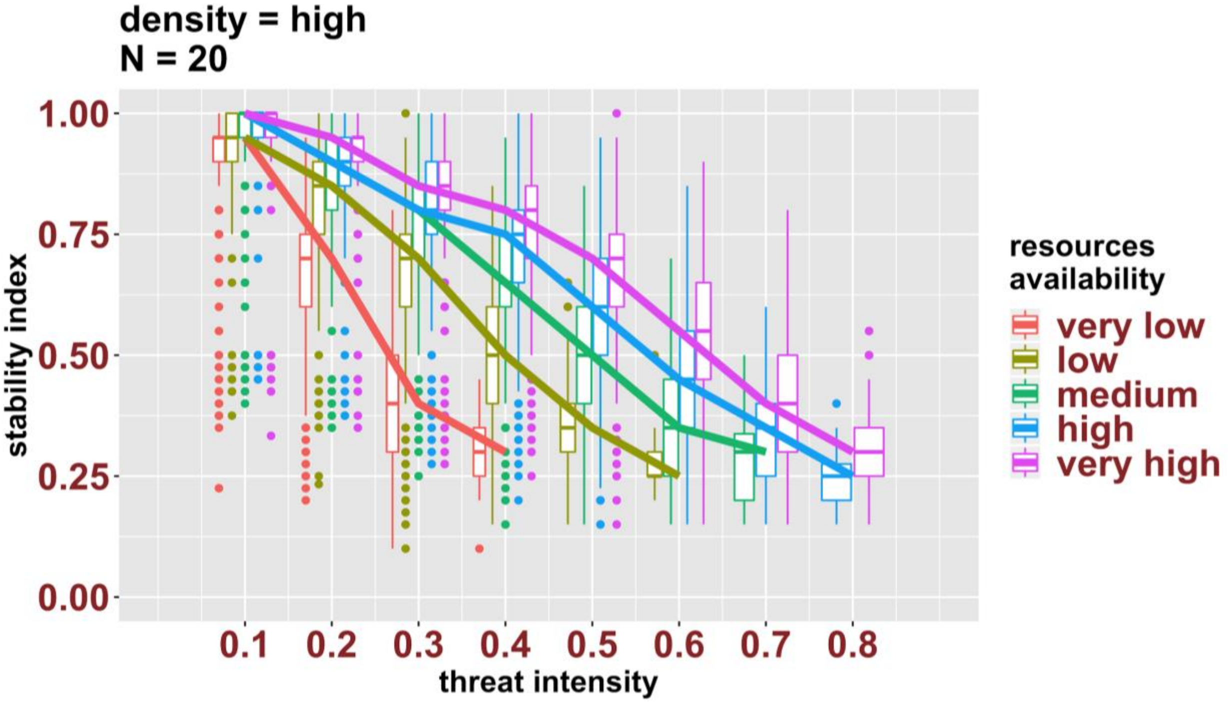

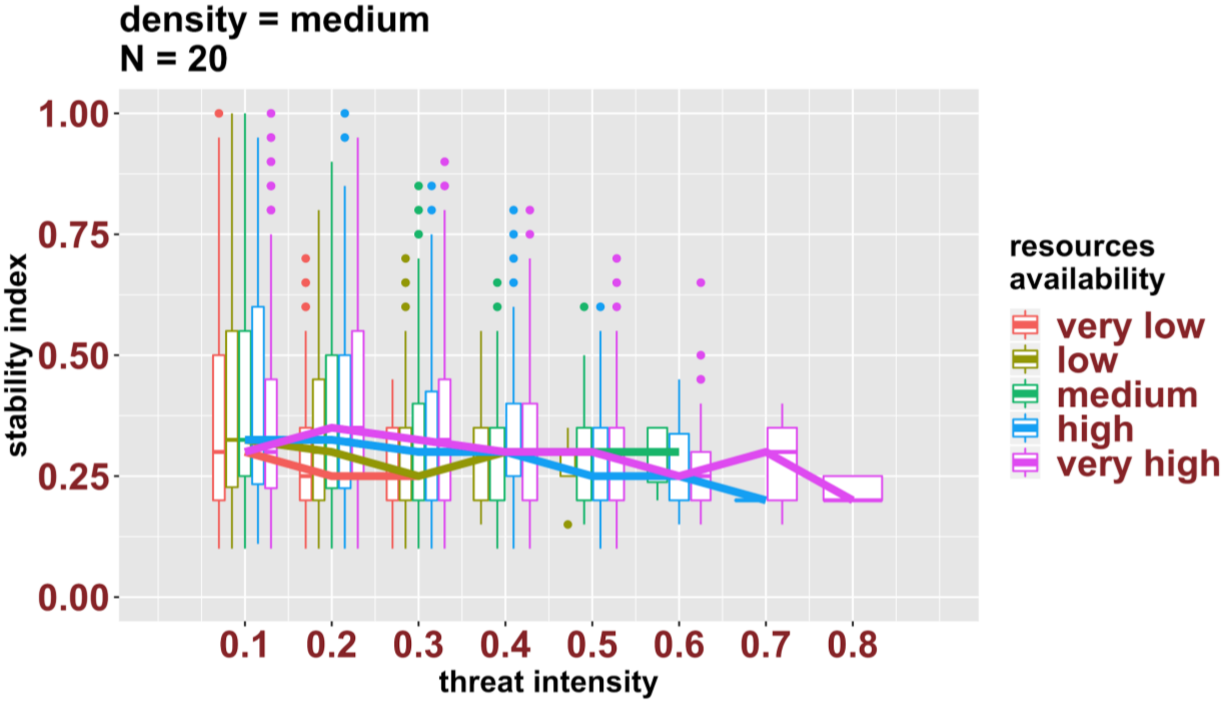

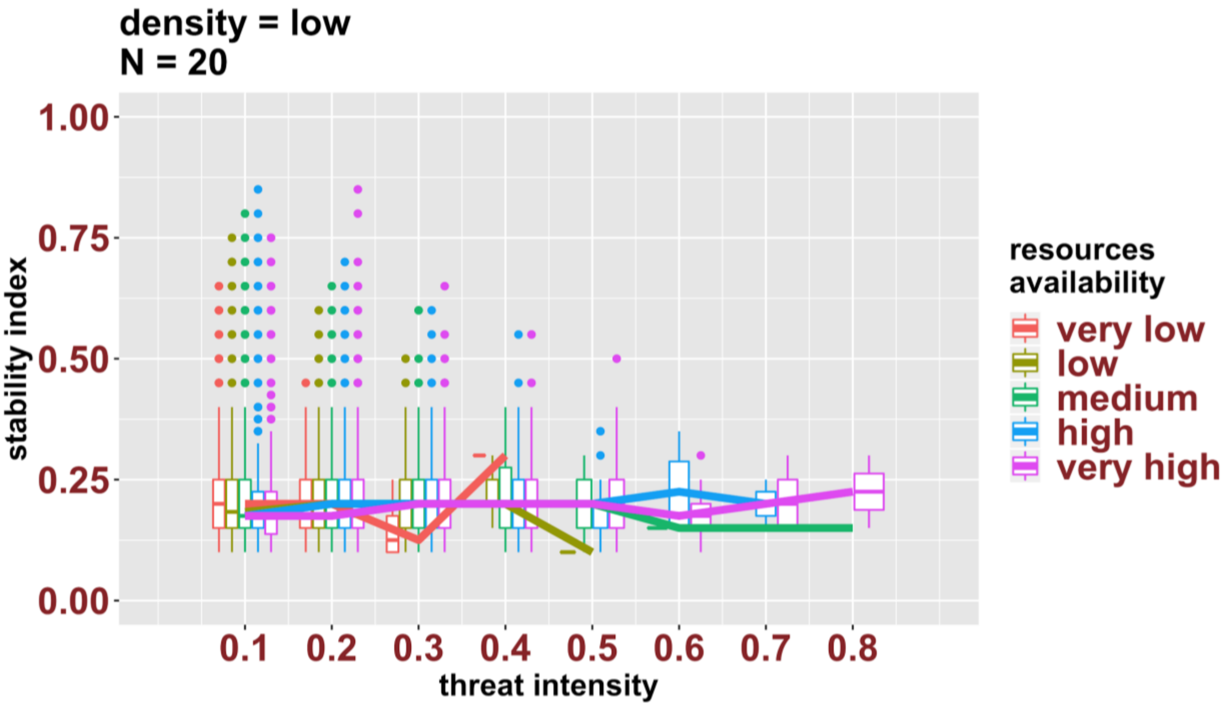

density = low
N = 20
stability index
1.00
0.75
0.50
0.25
0.00
0.1
0.2
0.3
0.4
0.5
0.6
0.7
0.8
threat intensity
resources
availability
very low
low
medium
high
very high

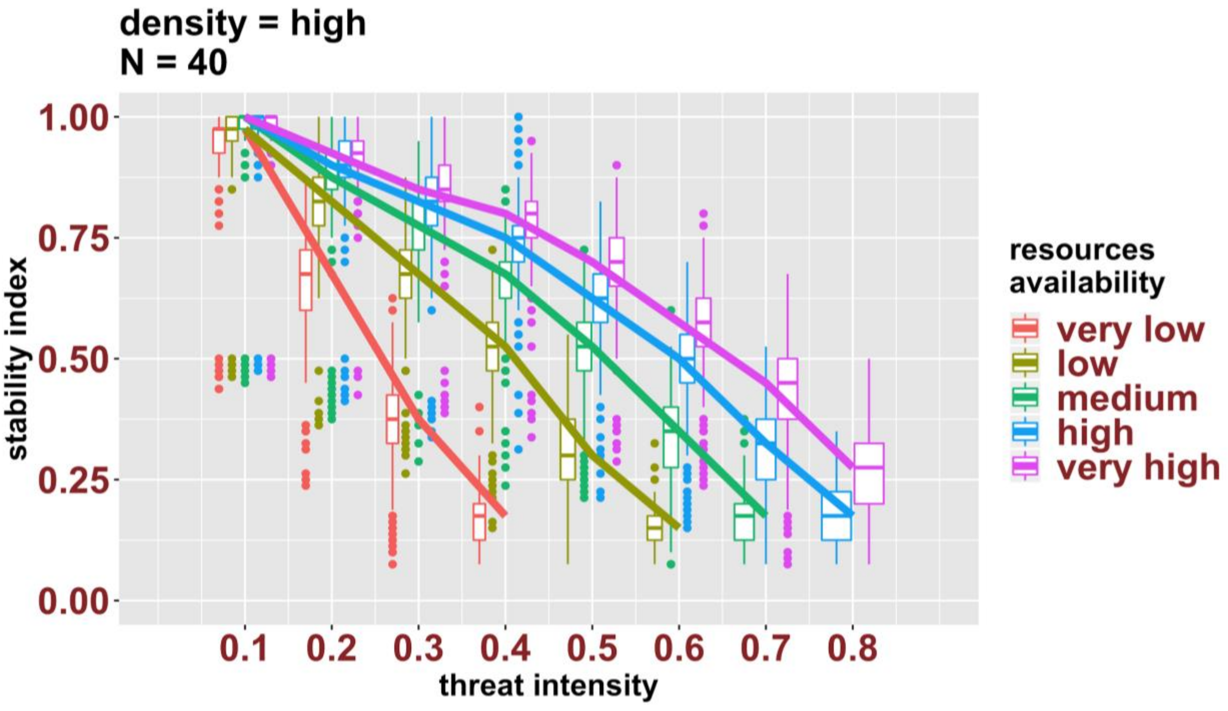

density = high
N = 40
stability index
1.00
0.75
0.50
0.25
0.00
0.1
0.2
0.3
0.4
0.5
0.6
0.7
0.8
threat intensity
resources
availability
very low
low
medium
high
very high

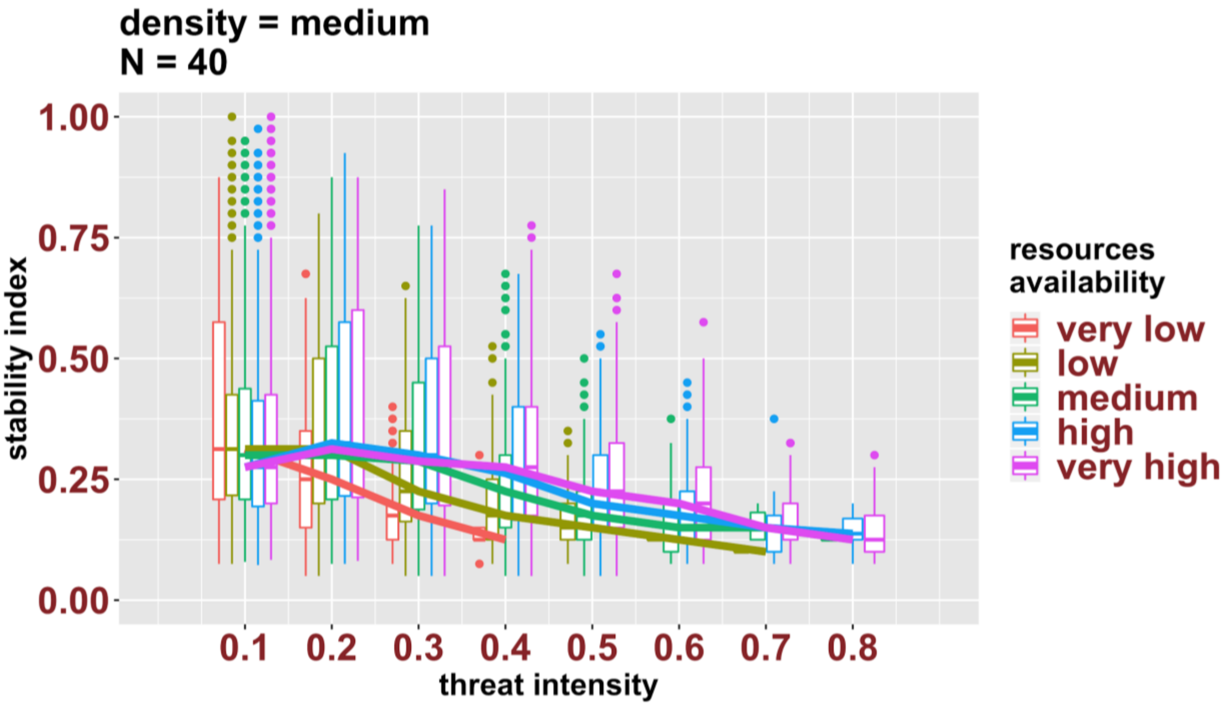
density = medium
N = 40
1.00
0.75
0.50
0.25
0.00
stability index
0.1
0.2
0.3
0.4
0.5
0.6
0.7
0.8
threat intensity
resources
availability
very low
low
medium
high
very high

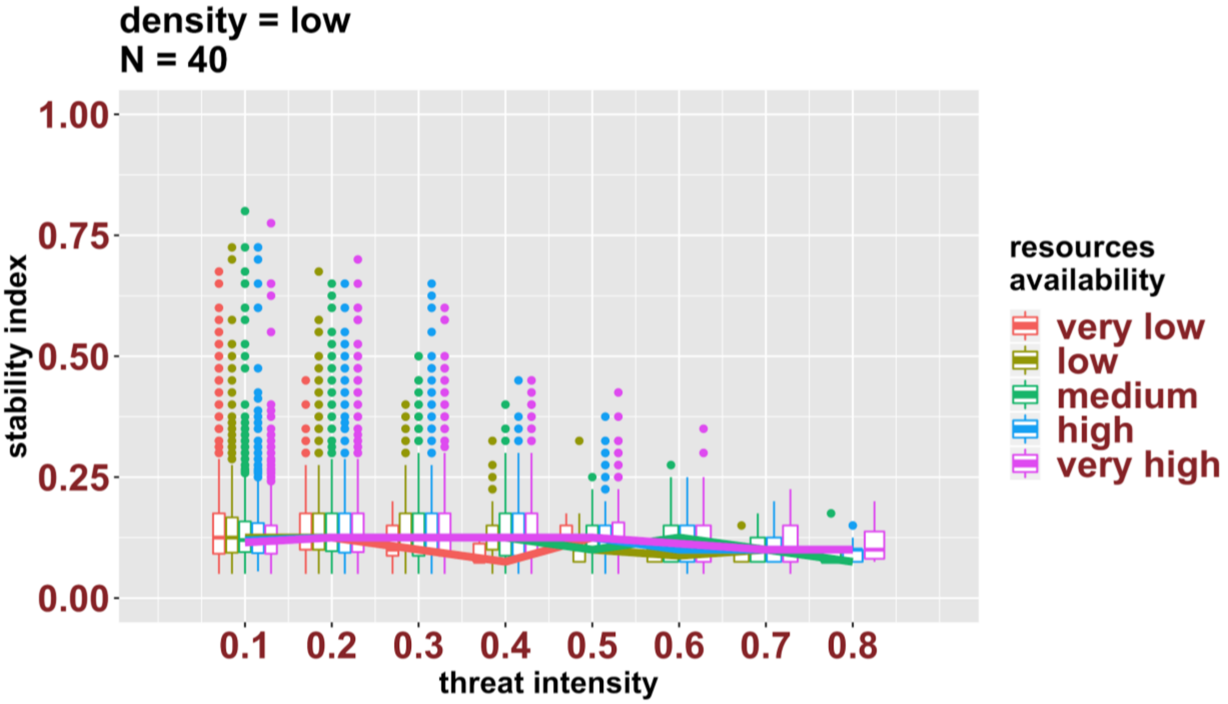
density = low
N = 40
1.00
0.75
0.50
0.25
0.00
stability index
0.1
0.2
0.3
0.4
0.5
0.6
0.7
0.8
threat intensity
resources
availability
very low
low
medium
high
very high

density = high
N = 80

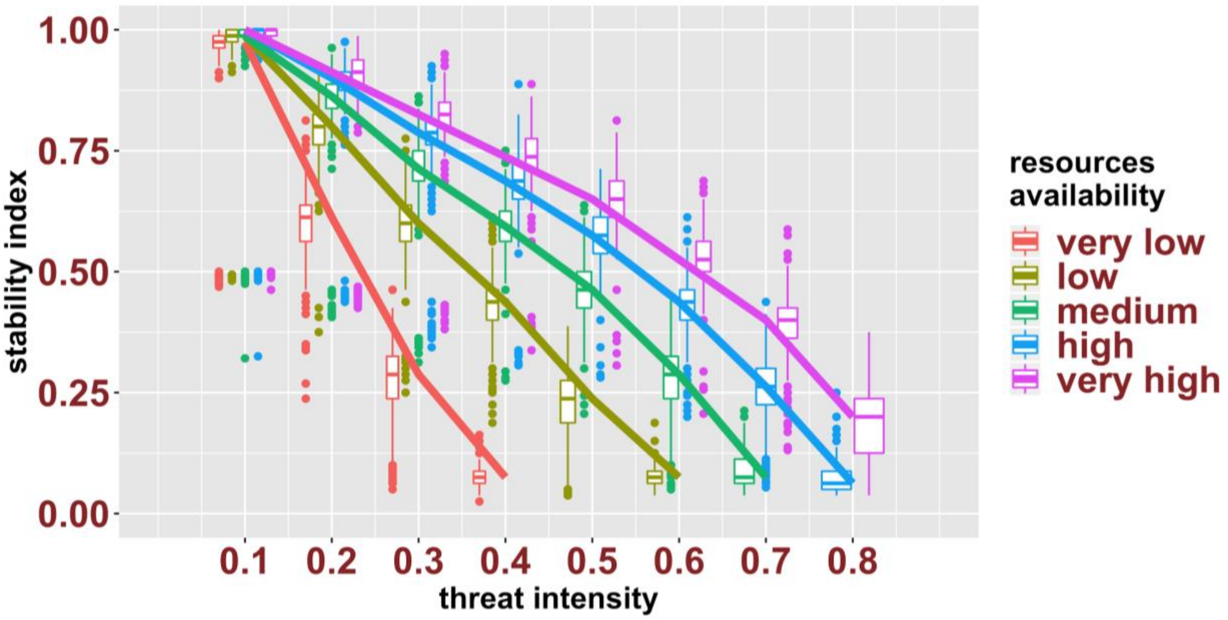

density = medium
N = 80

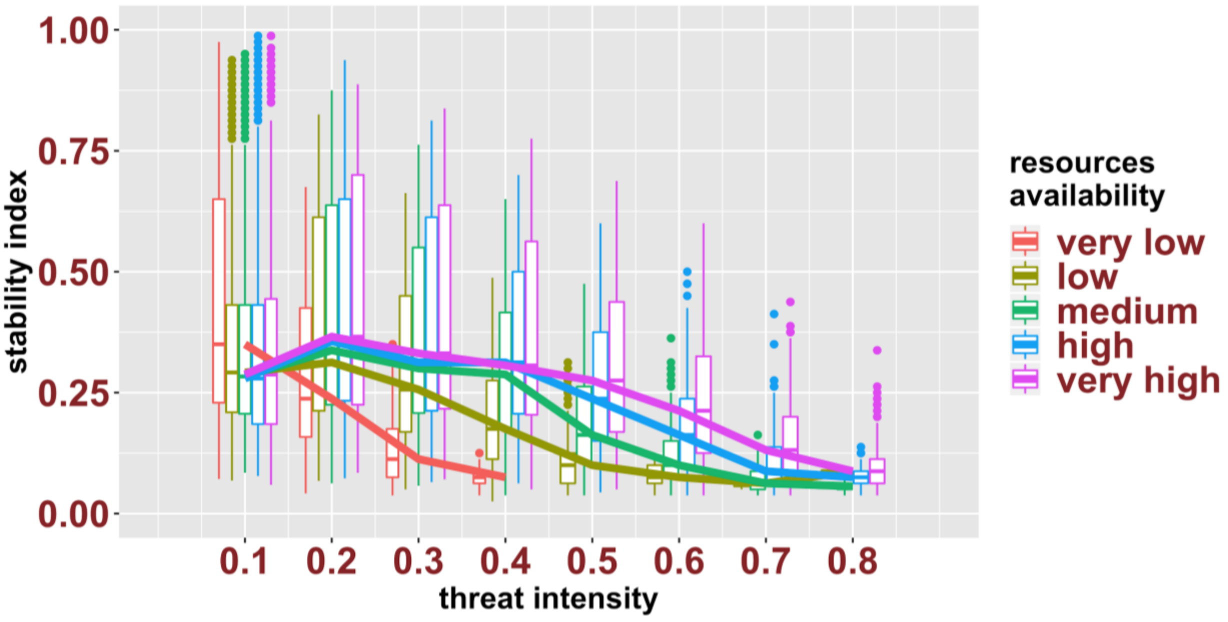

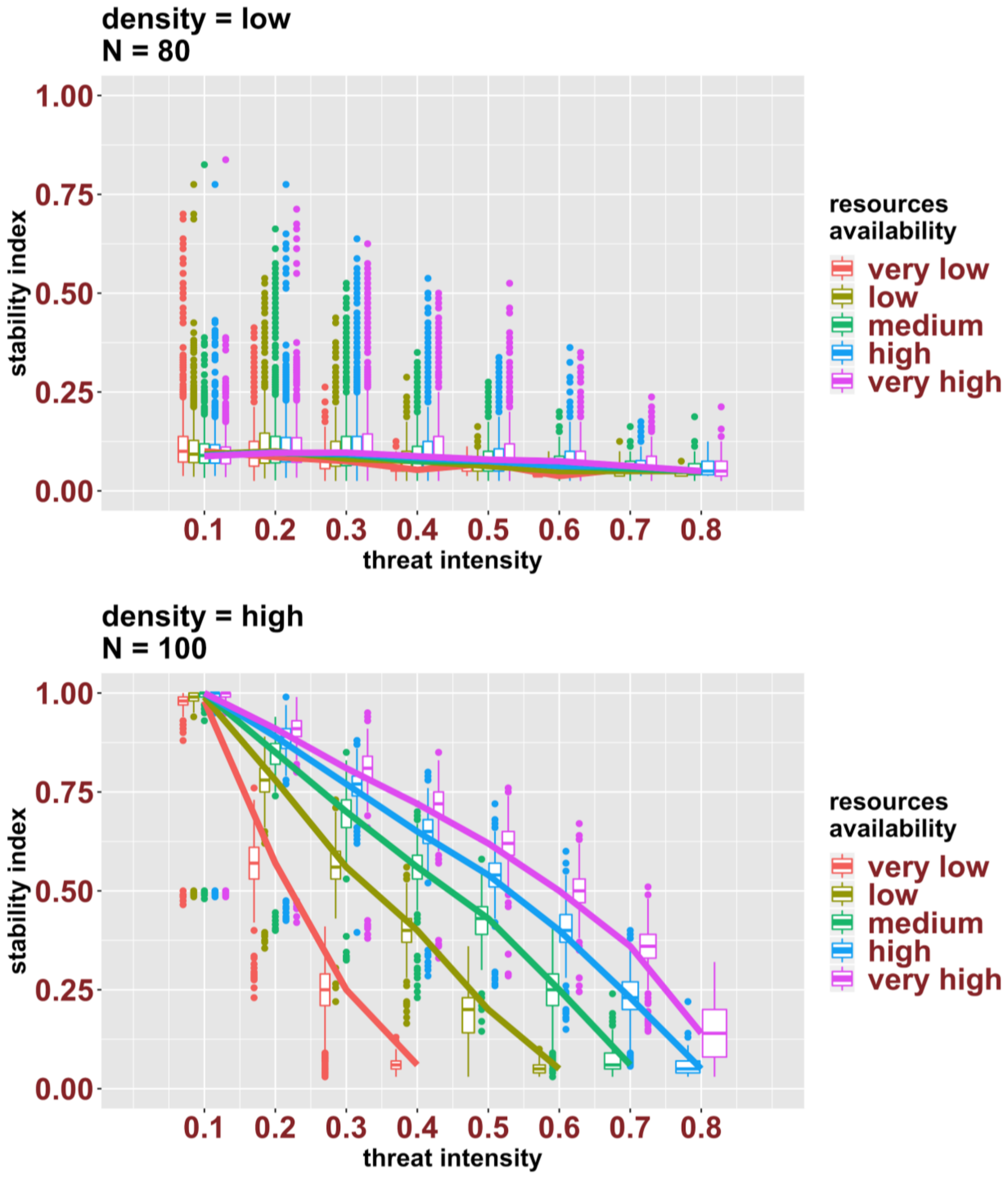
density = low
N = 80
stability index
1.00
0.75
0.50
0.25
0.00
0.1
0.2
0.3
0.4
0.5
0.6
0.7
0.8
threat intensity
resources
availability
very low
low
medium
high
very high
density = high
N = 100
stability index
1.00
0.75
0.50
0.25
0.00
0.1
0.2
0.3
0.4
0.5
0.6
0.7
0.8
threat intensity
resources
availability
very low
low
medium
high
very high

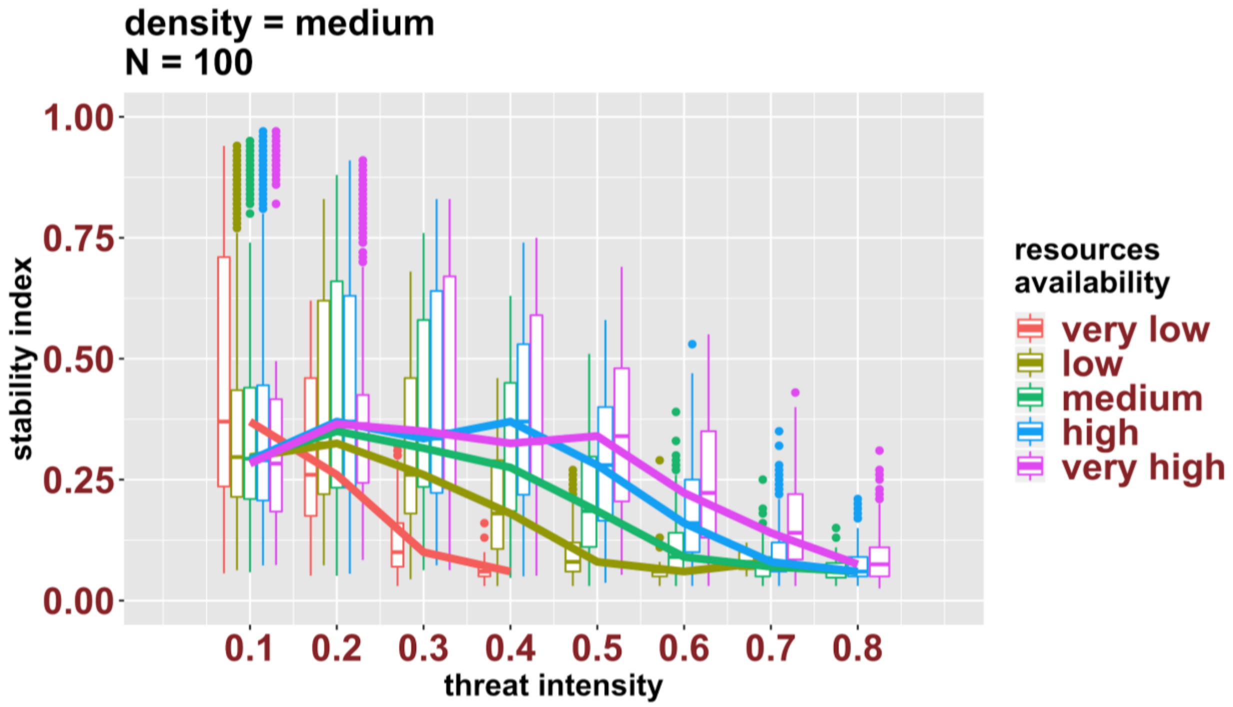
density = medium
N = 100
stability index
1.00
0.75
0.50
0.25
0.00
0.1
0.2
0.3
0.4
0.5
0.6
0.7
0.8
threat intensity
resources availability
very low
low
medium
high
very high

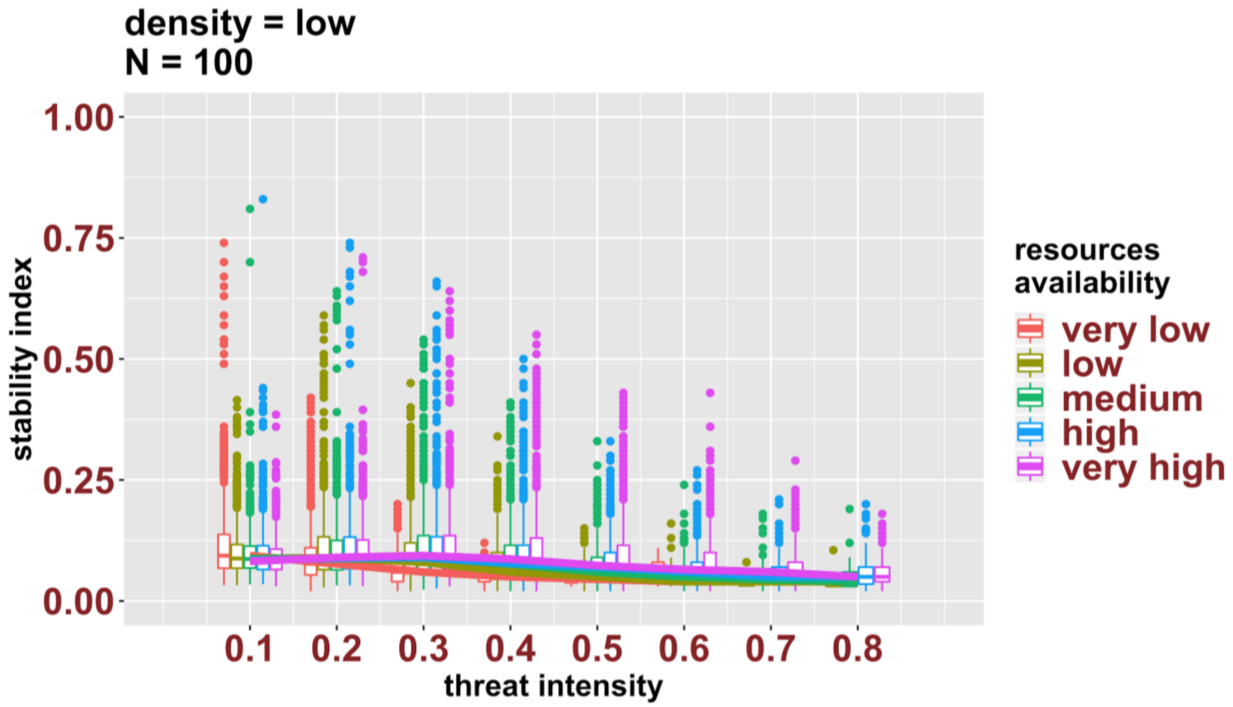
density = low
N = 100
stability index
1.00
0.75
0.50
0.25
0.00
0.1
0.2
0.3
0.4
0.5
0.6
0.7
0.8
threat intensity
resources availability
very low
low
medium
high
very high

4. Results of the Dynamics

Figures below show the dynamics of the *Survival Ratio* (SR), i.e., the values calculated over time (for details about SR, see the main text and Figure 3).

For each specific combination of parameters, plots of dynamics of *Survival Ratio* are presented.

Results are averaged 1000 replicates of the same combinations of parameters.

Figure S5. The impact of density and size on cluster resilience in optimal environments. Time is denoted on the x-axis (from 0 to 200), and y-axis indicates survival ratio. Panels are organized horizontally by initial number of cells (N) – from 5 to 100, and vertically by cluster density (2=high density, 4=medium density, and 6=low density).

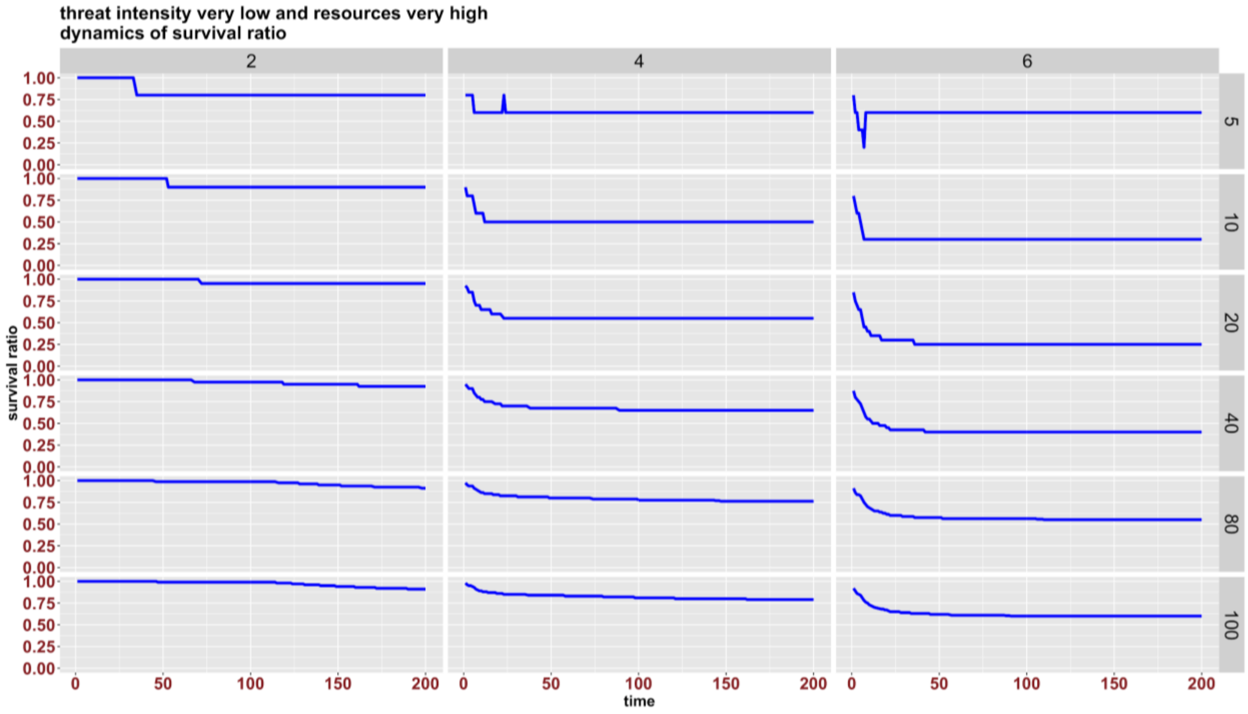

Figure S6. The effect of resource availability on cluster persistence and stability

Time is denoted on the x-axis (from 0 to 200), and y-axis indicates survival ratio. Panels are organized horizontally by initial number of cells (N) – from 5 to 100, and vertically by resources availability, from 4=very low to 20=very high; each separate figure shows results for a different cluster density.

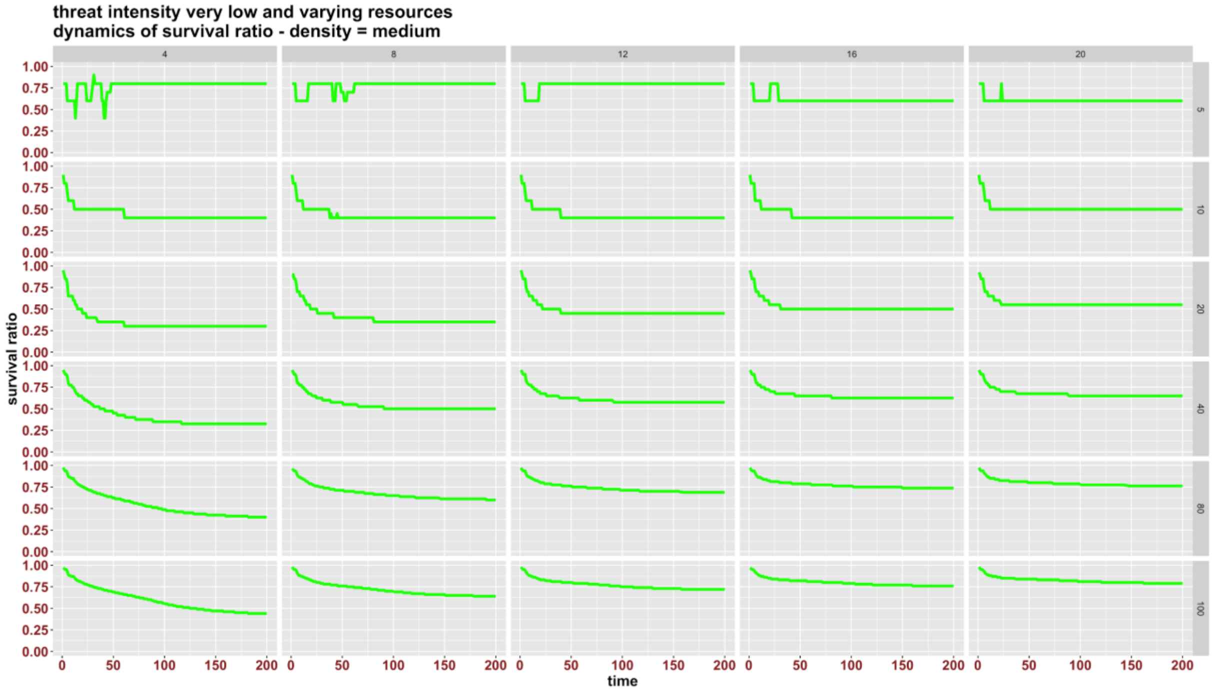

threat intensity very low and varying resources
dynamics of survival ratio - density = high

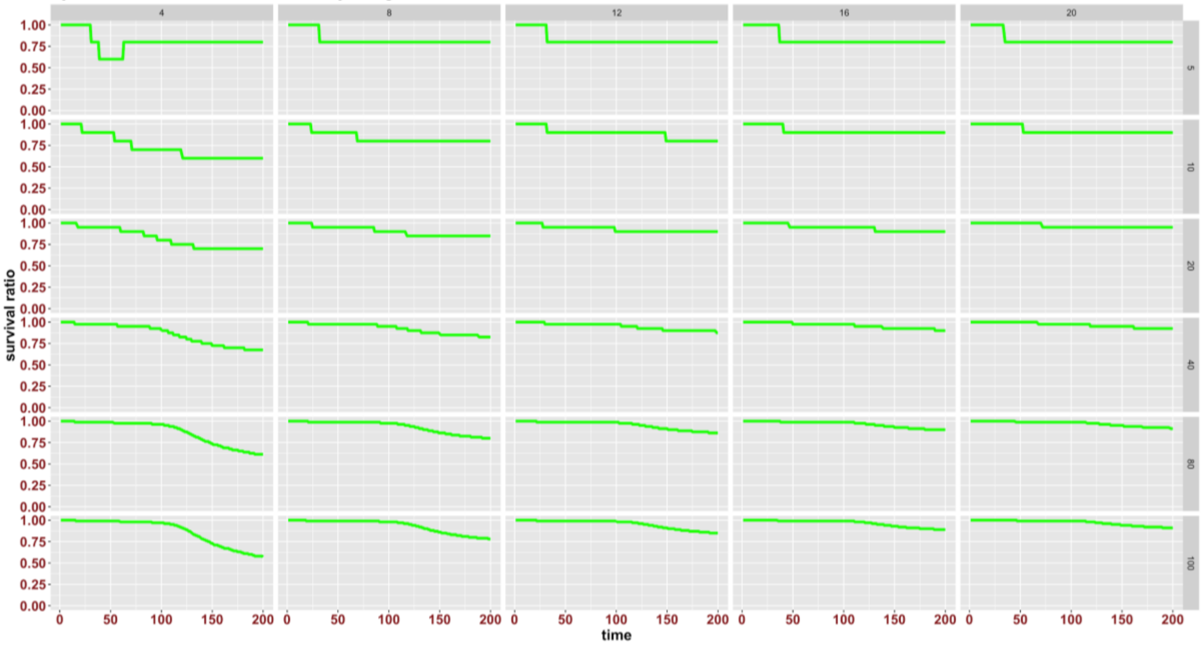

threat intensity very low and varying resources
dynamics of survival ratio - density = low

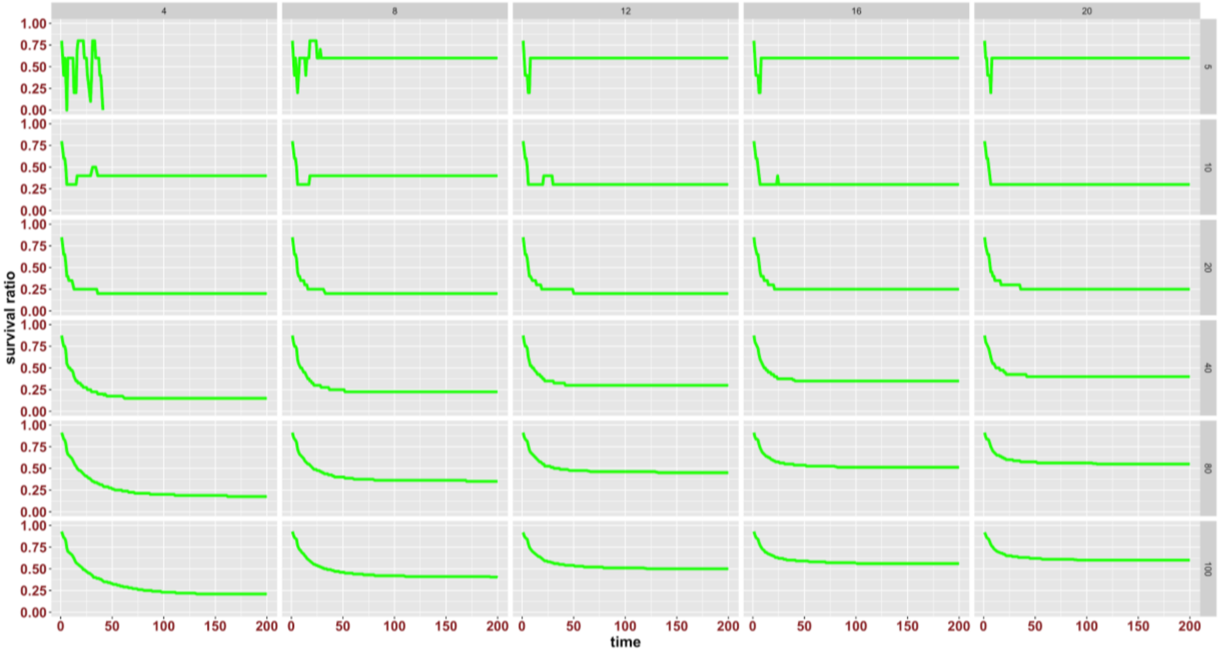

Figure S7. The effect of environmental threats on cluster persistence and stability
Time is denoted on the x-axis (from 0 to 200), and y-axis indicates survival ratio. Panels are organized horizontally by initial number of cells (N) – from 5 to 100, and vertically by threat intensity, where 0.1 is a very low threat intensity and 0.8 is a very high threat intensity; each separate figure shows results for a different cluster density.

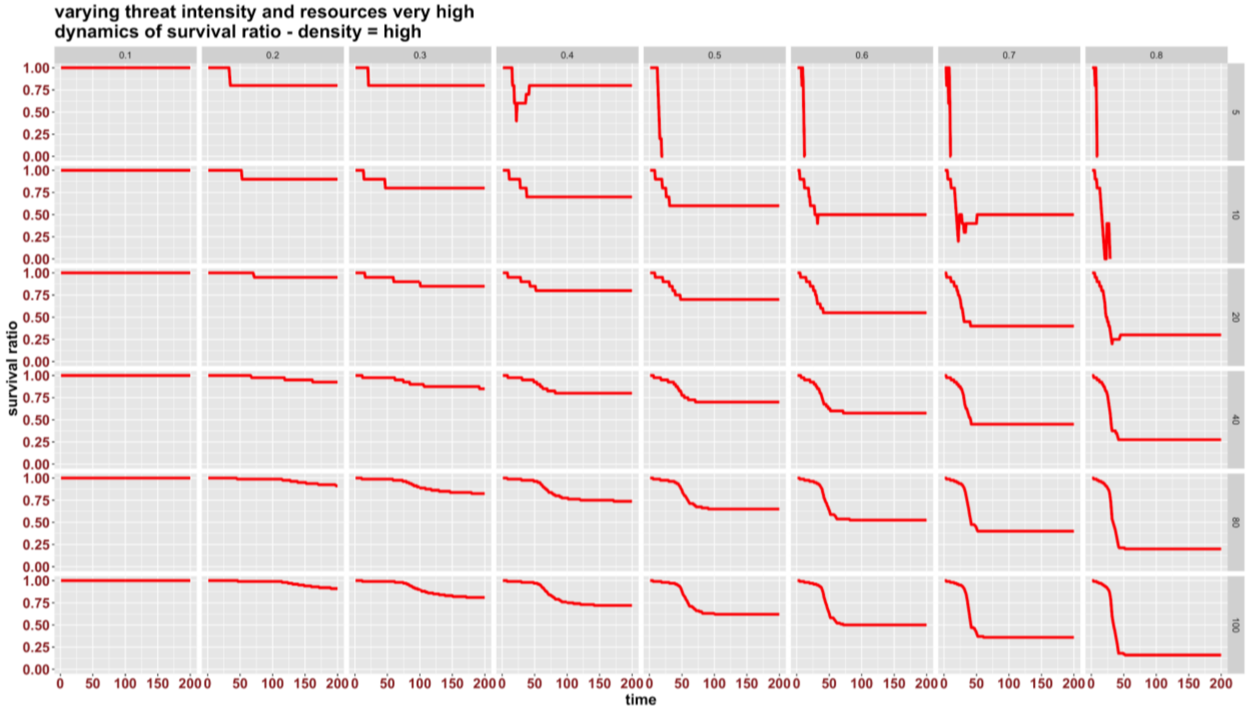

varying threat intensity and resources very high
dynamics of survival ratio - density = medium

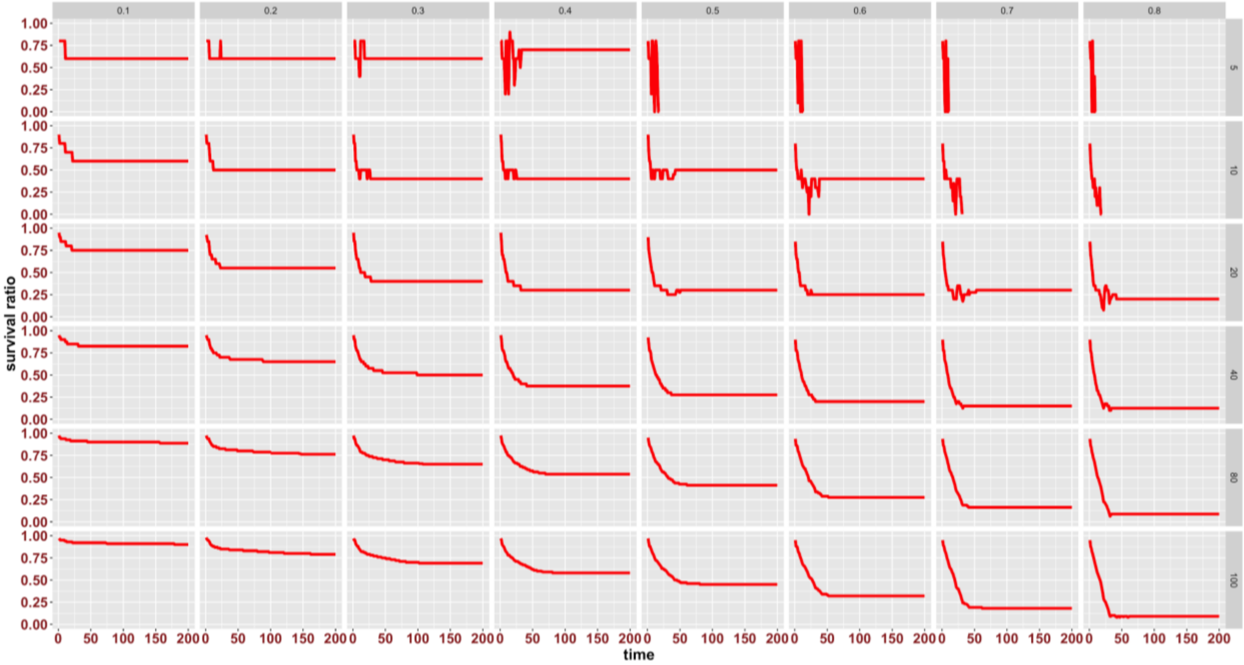

varying threat intensity and resources very high
dynamics of survival ratio - density = low

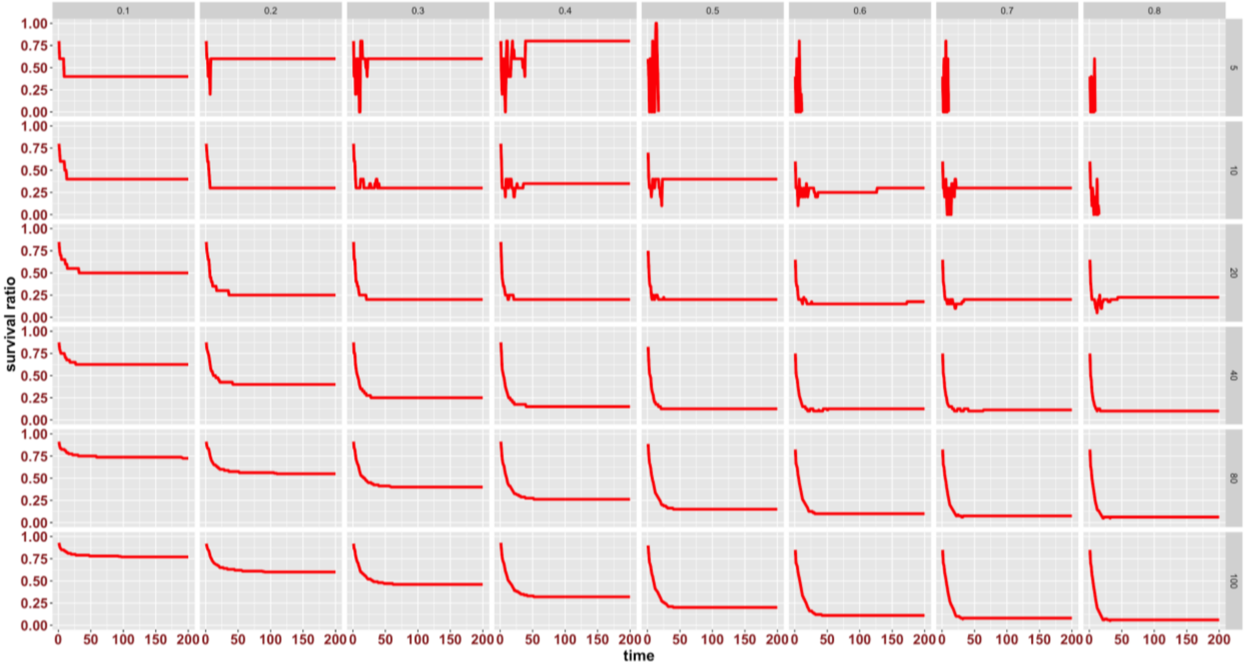

Figure S8. The combined effect of environmental threats and resource availability on cluster survival

Time is denoted on the x-axis (from 0 to 200), and y-axis indicates survival ratio. Panels are organized horizontally by threat intensity, where 0.1 is a very low threat intensity and 0.8 is a very high threat intensity, and vertically by resources availability, from 4=very low to 20=very high; each separate figure shows results for a different combination of cluster size N and cluster density.

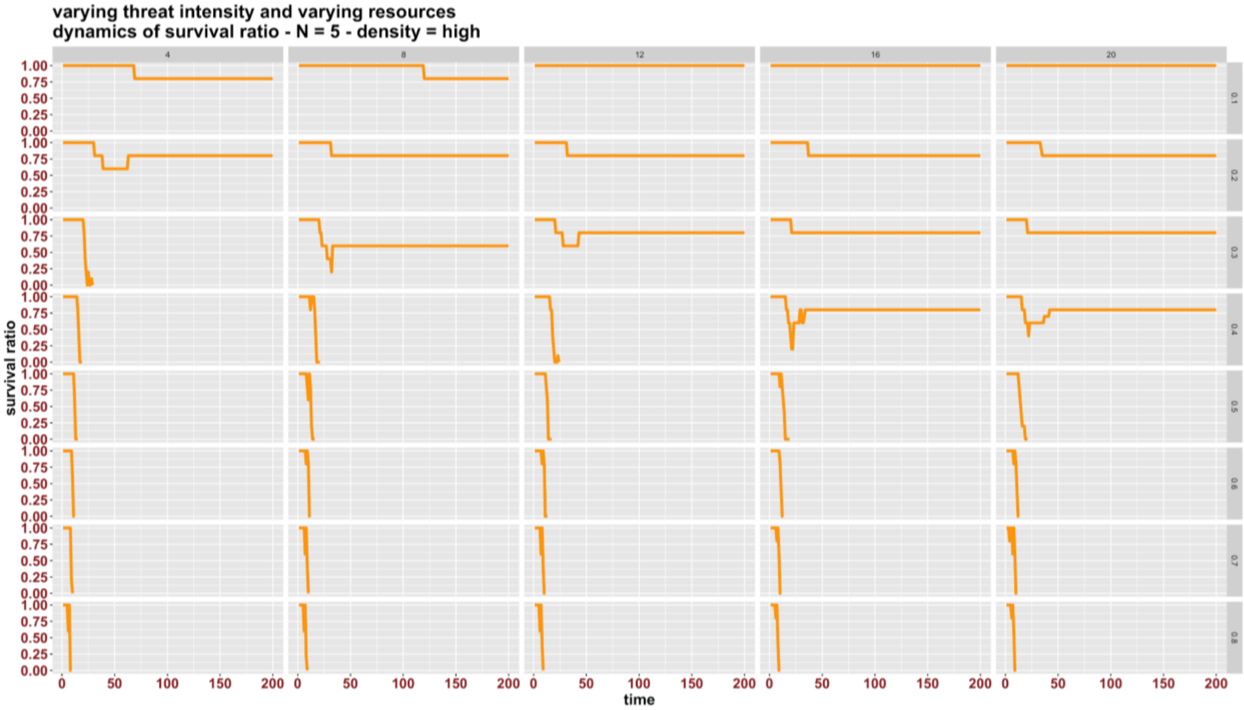

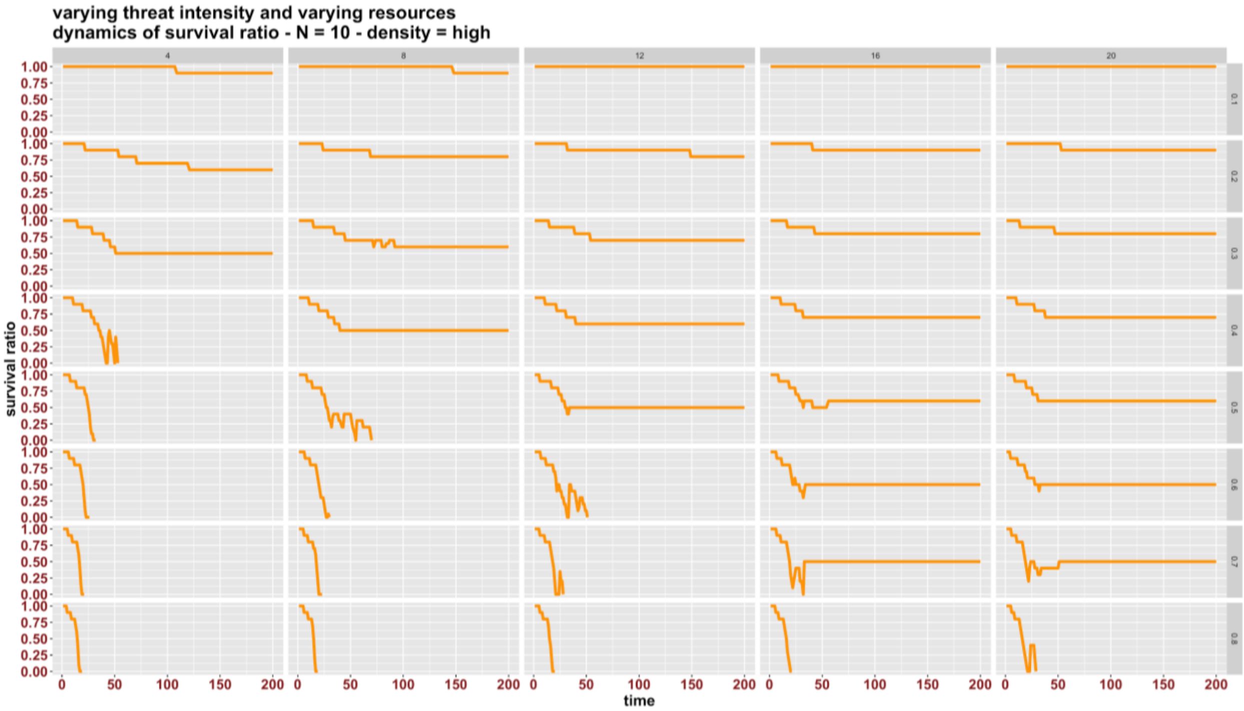
varying threat intensity and varying resources
dynamics of survival ratio - N = 10 - density = high
4
8
12
16
20
0.1
0.2
0.3
0.4
0.5
0.6
0.7
0.8
survival ratio
1.00
0.75
0.50
0.25
0.00
0
50
100
150
200
time

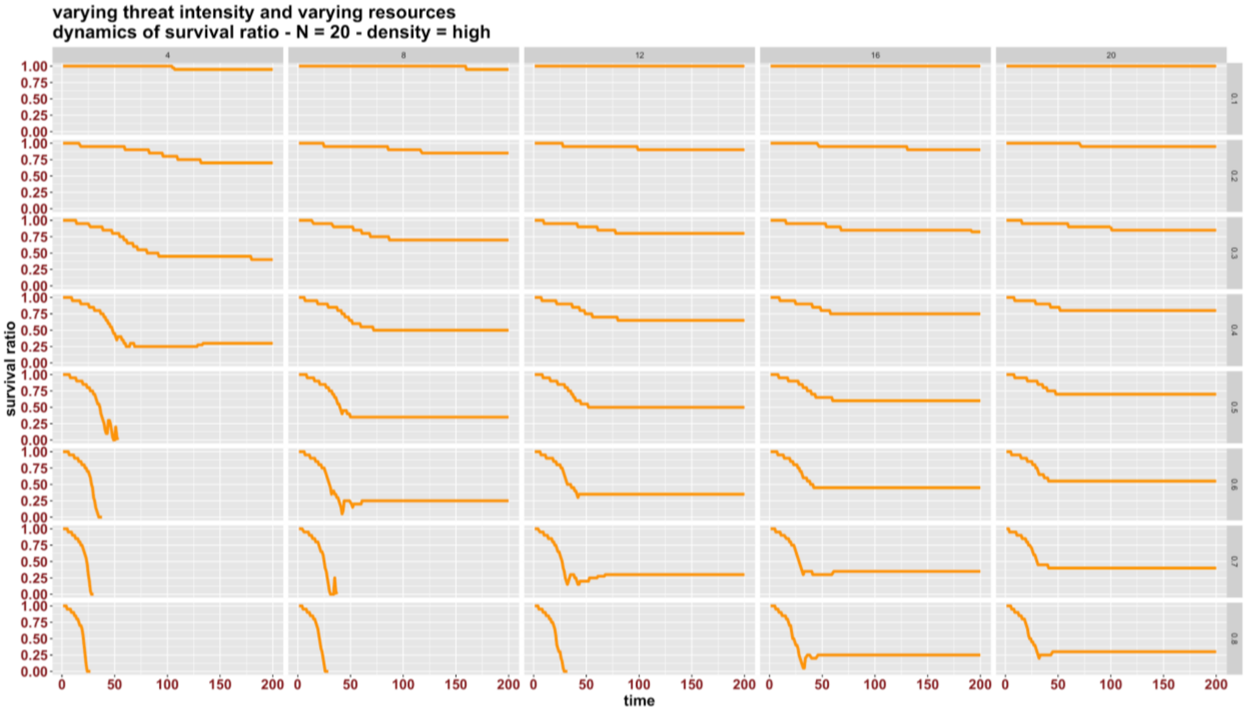
varying threat intensity and varying resources
dynamics of survival ratio - N = 20 - density = high
4
8
12
16
20
0.1
0.2
0.3
0.4
0.5
0.6
0.7
0.8
survival ratio
1.00
0.75
0.50
0.25
0.00
0
50
100
150
200
time

varying threat intensity and varying resources
dynamics of survival ratio - N = 40 - density = high

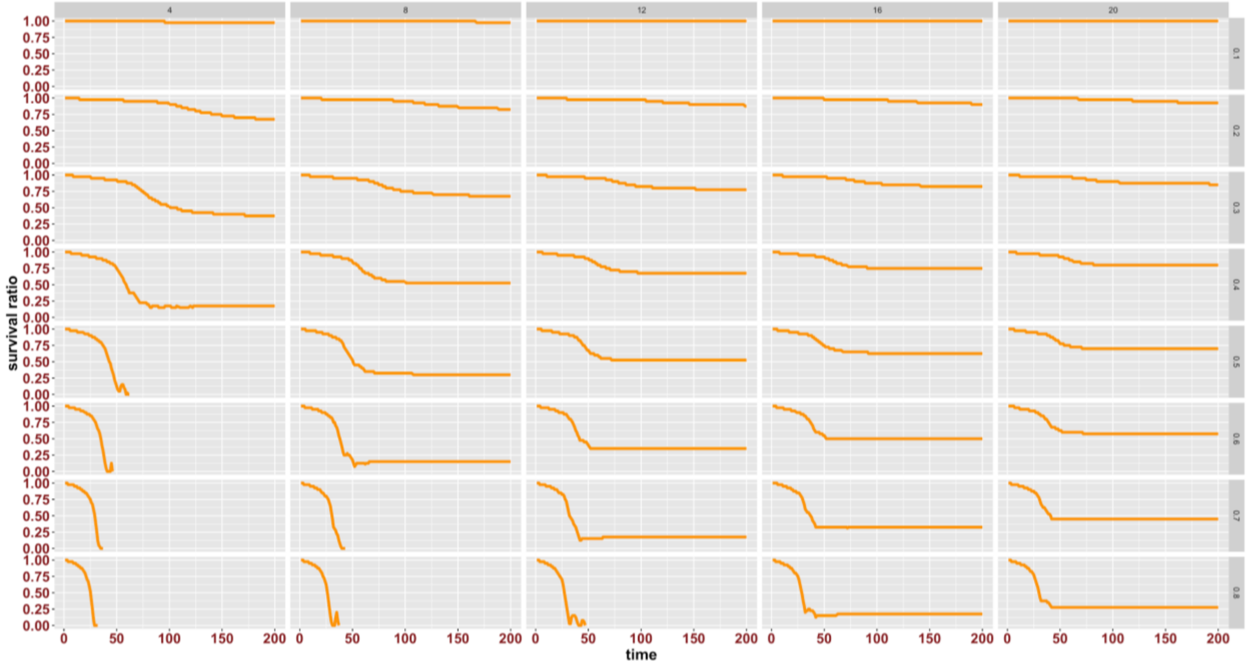

varying threat intensity and varying resources
dynamics of survival ratio - N = 80 - density = high

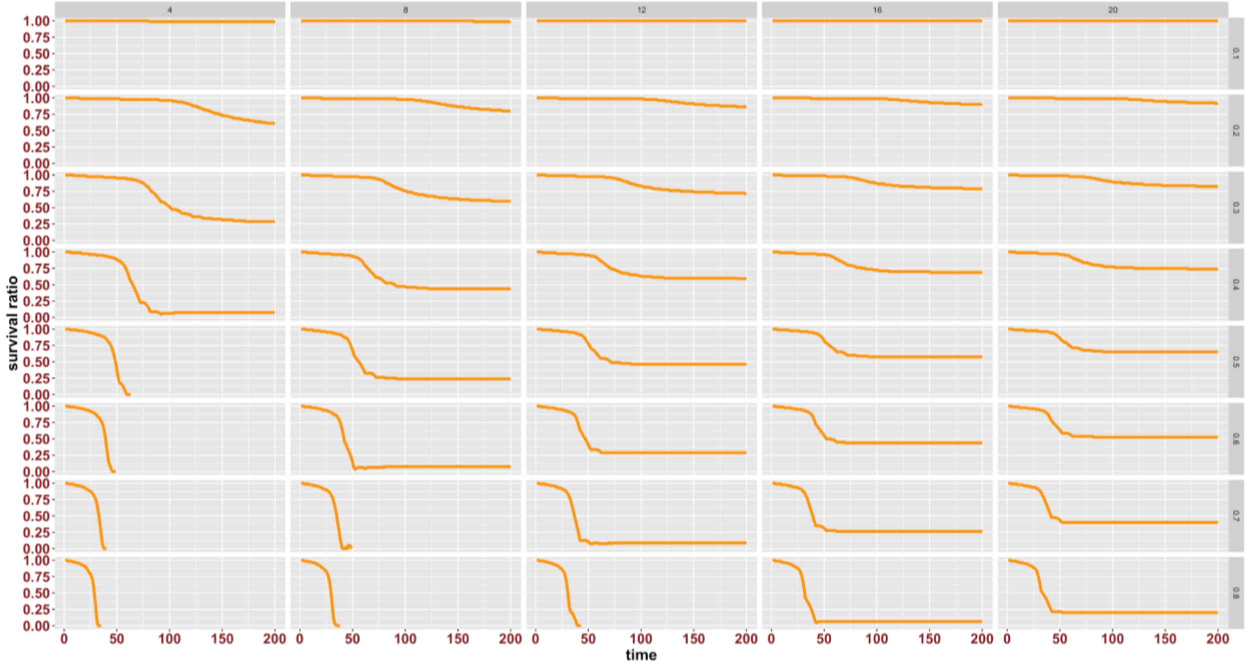

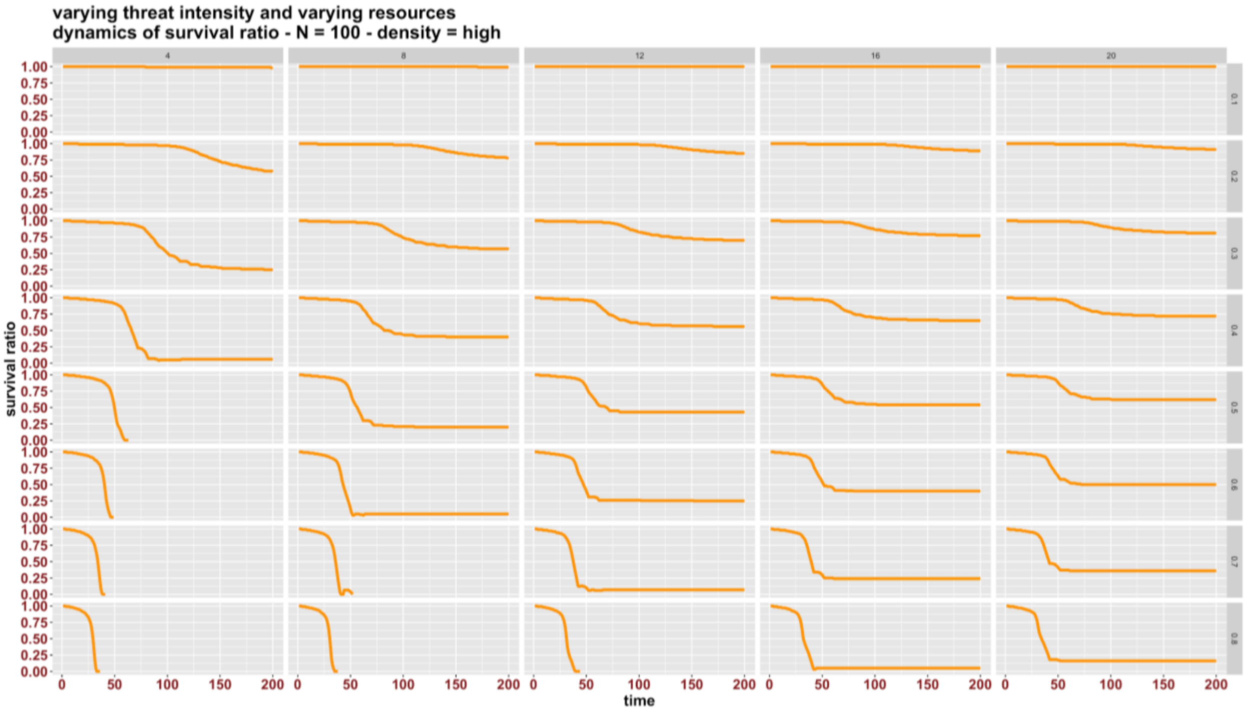
varying threat intensity and varying resources
dynamics of survival ratio - N = 100 - density = high
4
8
12
16
20
0.1
0.2
0.3
0.4
0.5
0.6
0.7
0.8
survival ratio
1.00
0.75
0.50
0.25
0.00
0
50
100
150
200
time

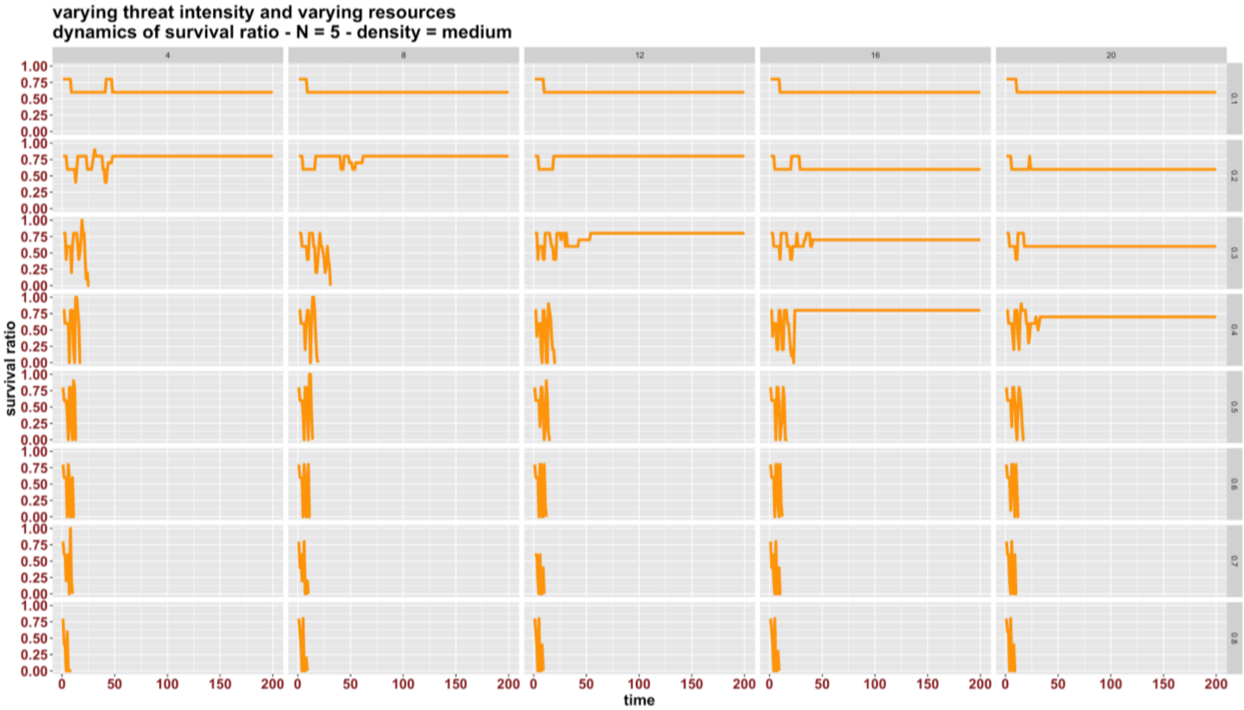
varying threat intensity and varying resources
dynamics of survival ratio - N = 5 - density = medium
4
8
12
16
20
0.1
0.2
0.3
0.4
0.5
0.6
0.7
0.8
survival ratio
1.00
0.75
0.50
0.25
0.00
0
50
100
150
200
time

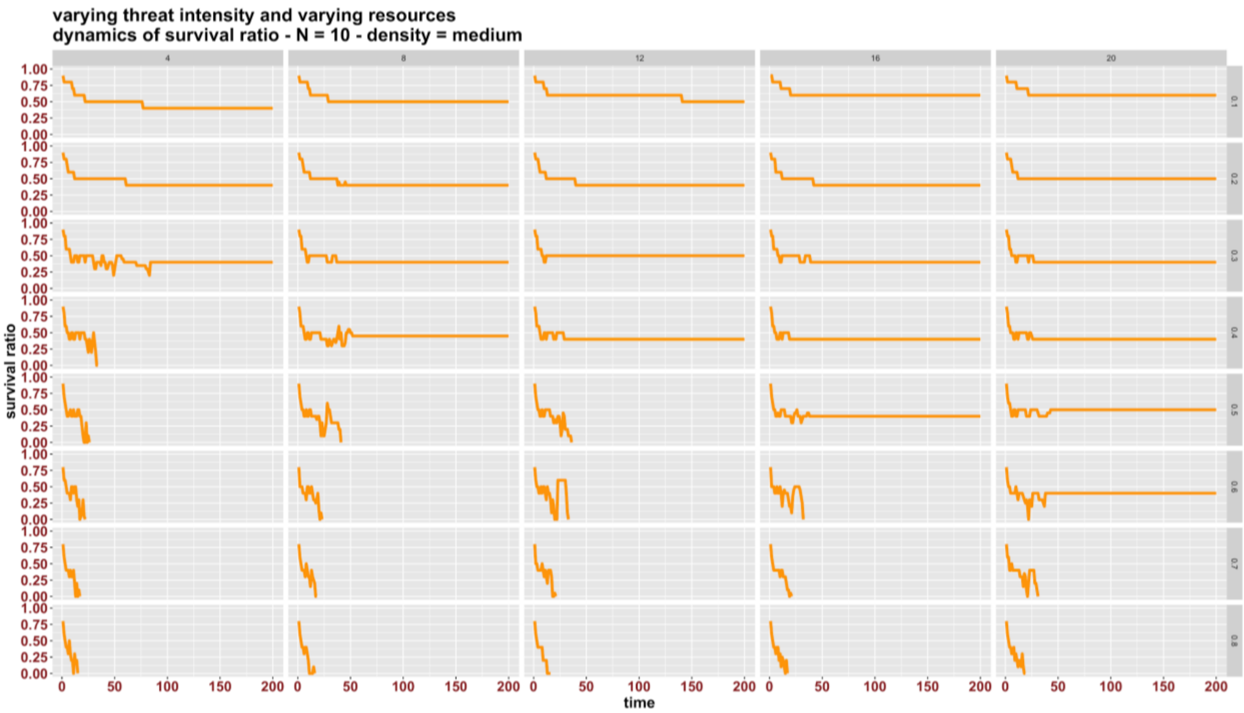
varying threat intensity and varying resources
dynamics of survival ratio - N = 10 - density = medium
4
8
12
16
20
0.1
0.2
0.3
0.4
0.5
0.6
0.7
0.8
survival ratio
1.00
0.75
0.50
0.25
0.00
0
50
100
150
200
time

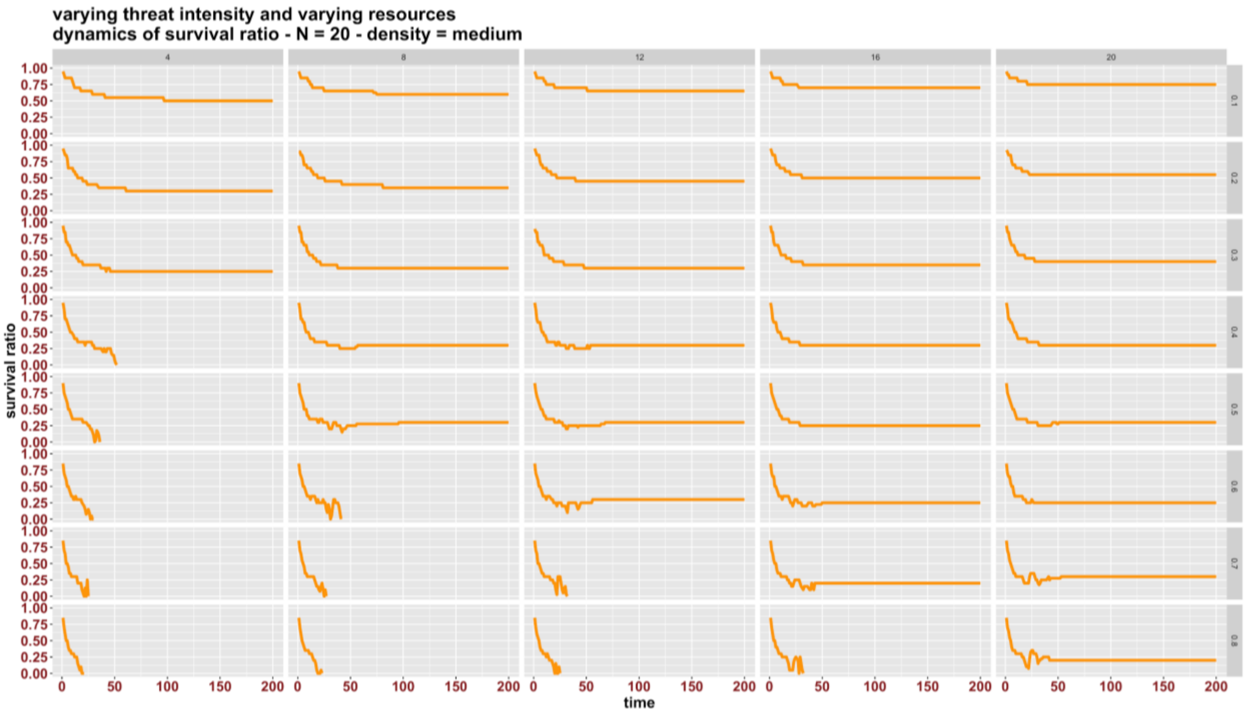
varying threat intensity and varying resources
dynamics of survival ratio - N = 20 - density = medium
4
8
12
16
20
0.1
0.2
0.3
0.4
0.5
0.6
0.7
0.8
survival ratio
1.00
0.75
0.50
0.25
0.00
0
50
100
150
200
time

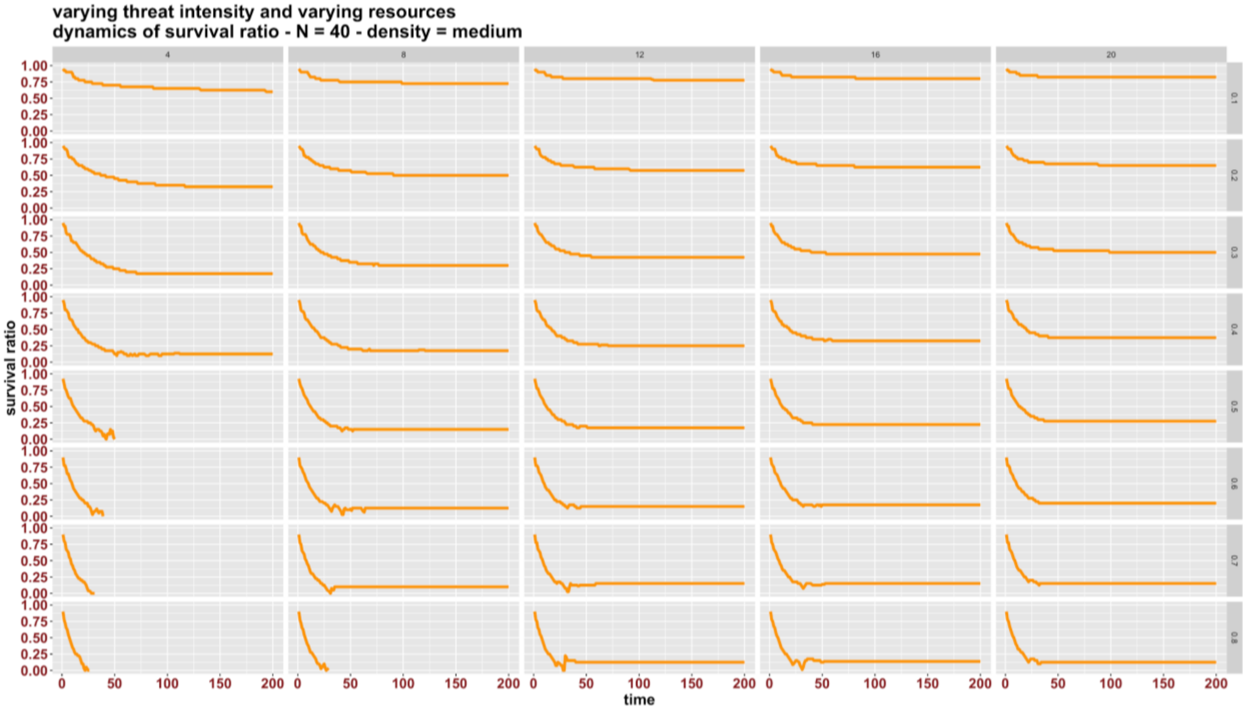
varying threat intensity and varying resources
dynamics of survival ratio - N = 40 - density = medium
4
8
12
16
20
0.1
0.2
0.3
0.4
0.5
0.6
0.7
0.8
survival ratio
time
0.00
0.25
0.50
0.75
1.00
0
50
100
150
200

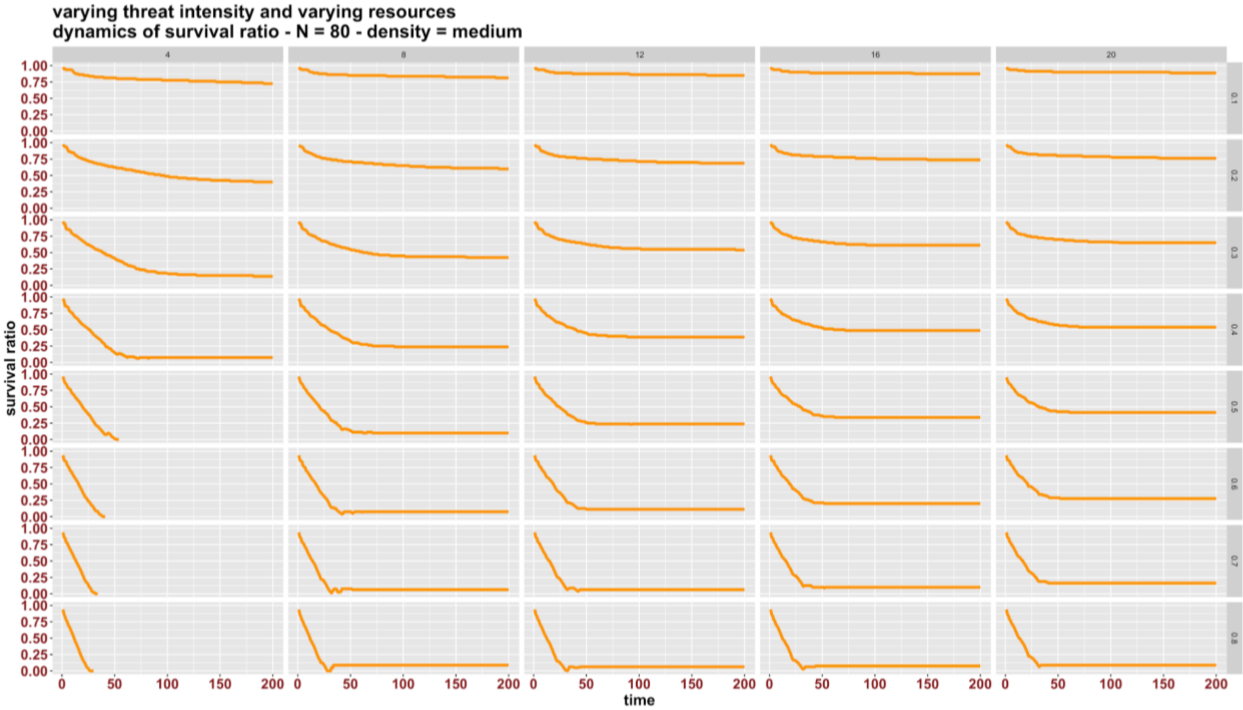
varying threat intensity and varying resources
dynamics of survival ratio - N = 80 - density = medium
4
8
12
16
20
0.1
0.2
0.3
0.4
0.5
0.6
0.7
0.8
survival ratio
time
0.00
0.25
0.50
0.75
1.00
0
50
100
150
200

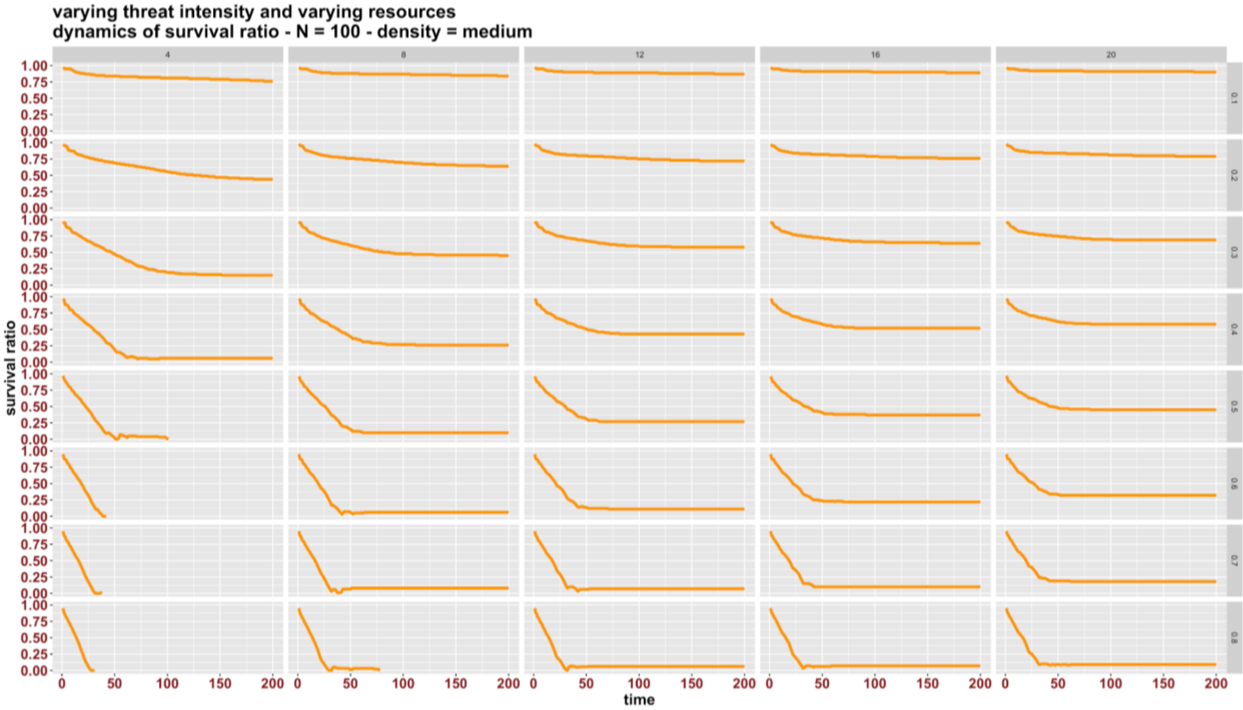
varying threat intensity and varying resources
dynamics of survival ratio - N = 100 - density = medium
4
8
12
16
20
0.1
0.2
0.3
0.4
0.5
0.6
0.7
0.8
1.00
0.75
0.50
0.25
0.00
survival ratio
0
50
100
150
200
time

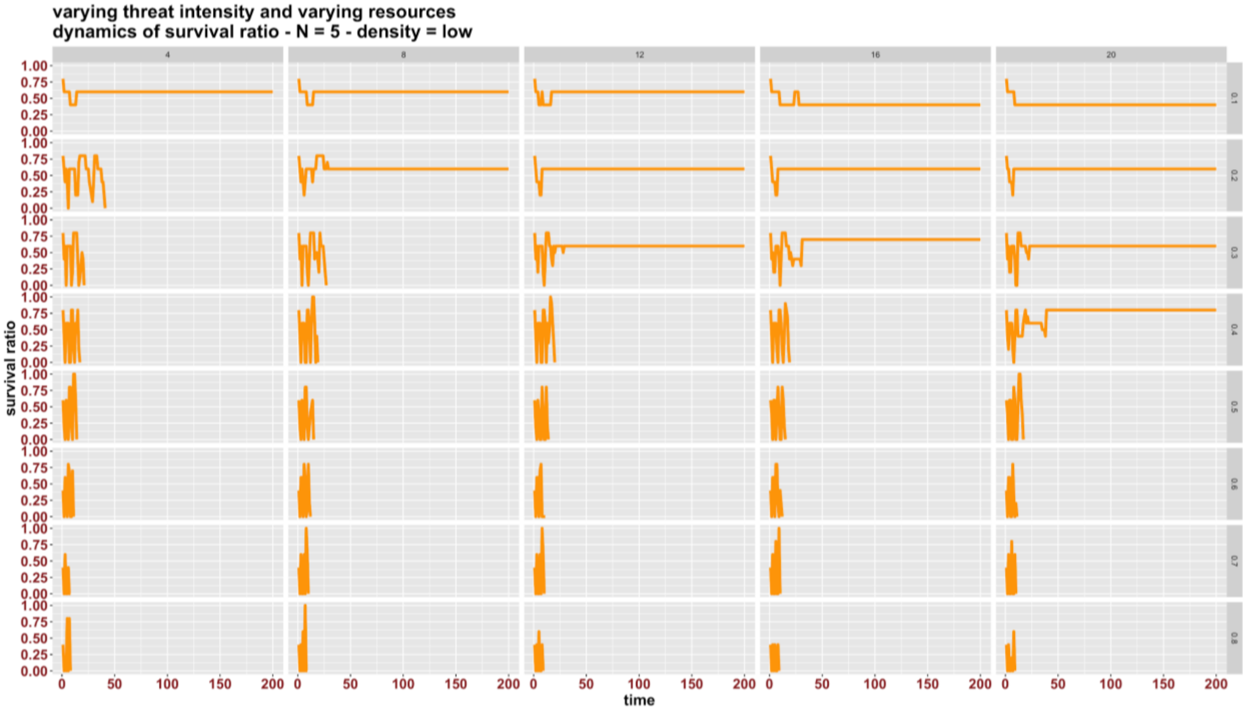
varying threat intensity and varying resources
dynamics of survival ratio - N = 5 - density = low
4
8
12
16
20
0.1
0.2
0.3
0.4
0.5
0.6
0.7
0.8
1.00
0.75
0.50
0.25
0.00
survival ratio
0
50
100
150
200
time

varying threat intensity and varying resources
dynamics of survival ratio - N = 10 - density = low

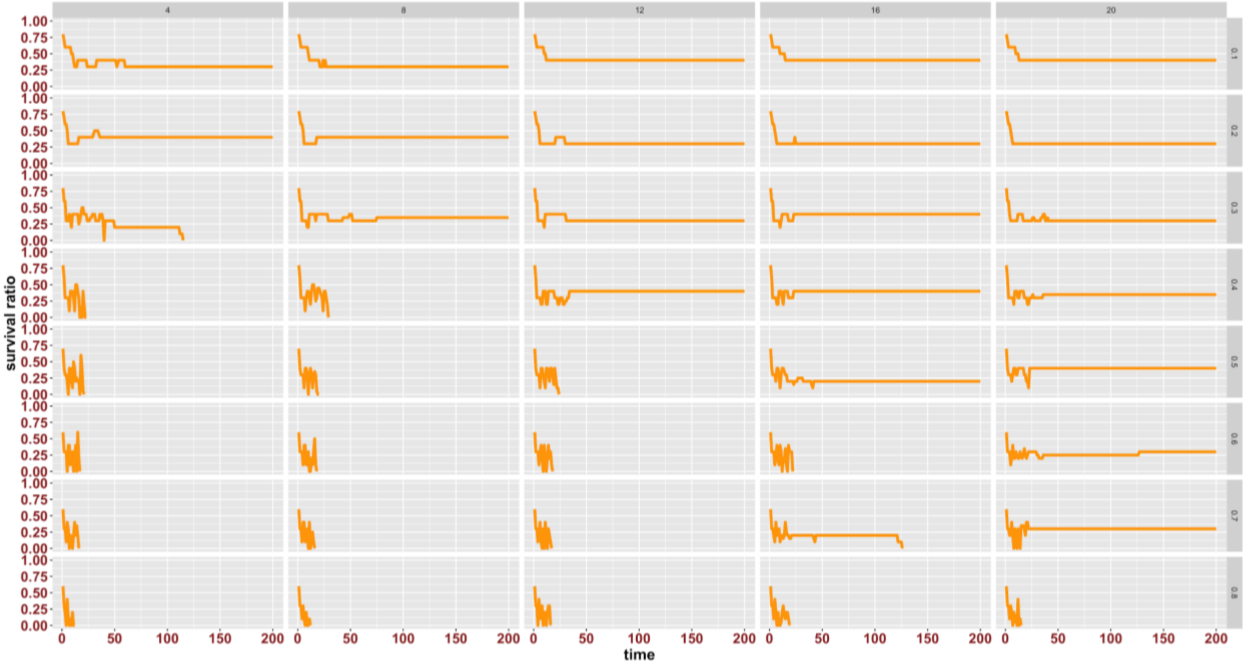

varying threat intensity and varying resources
dynamics of survival ratio - N = 20 - density = low

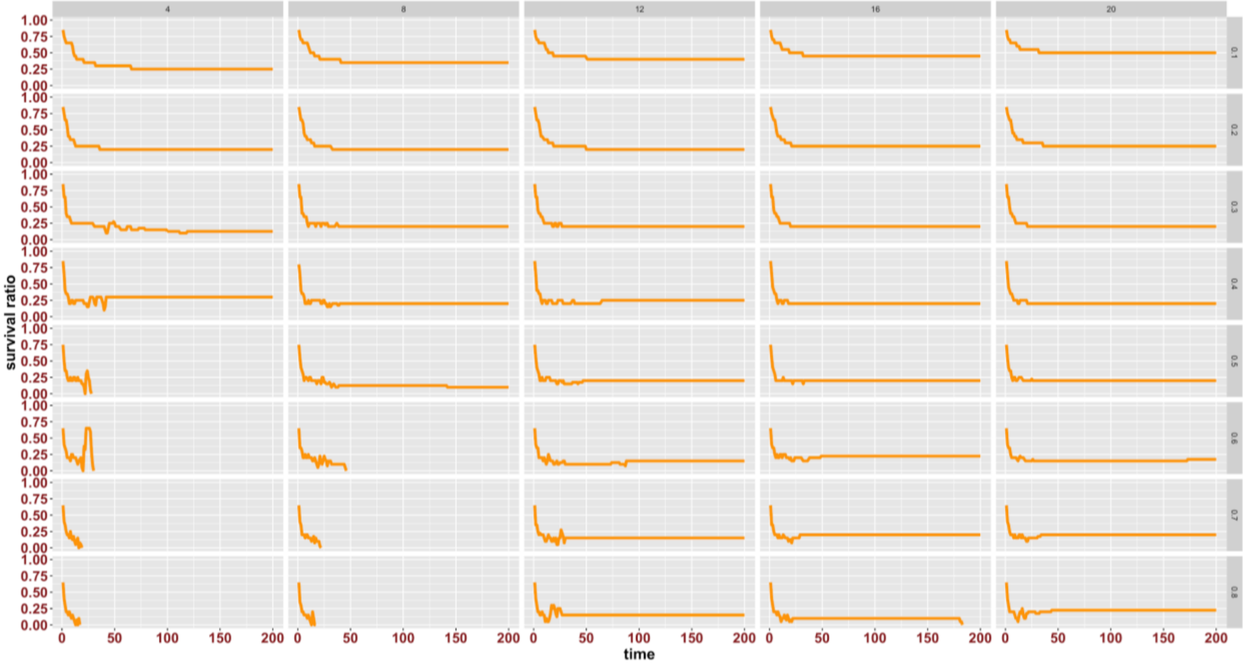

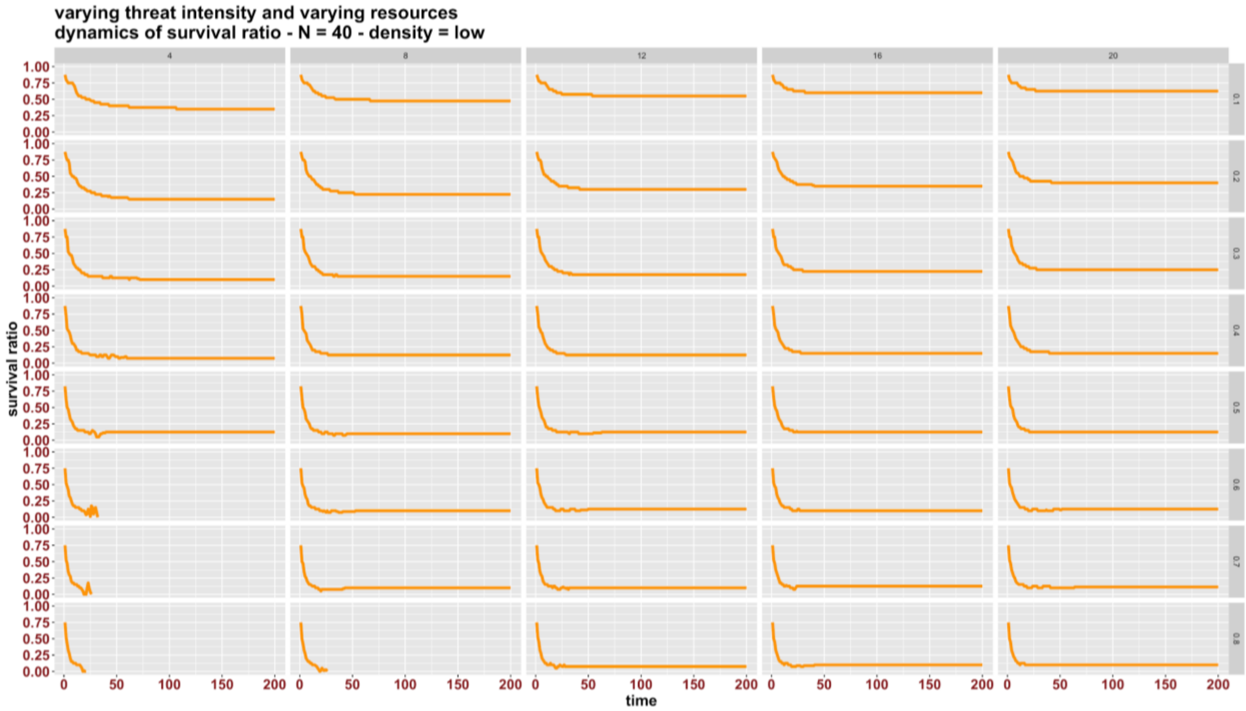
varying threat intensity and varying resources
dynamics of survival ratio - N = 40 - density = low
4
8
12
16
20
0.1
0.2
0.3
0.4
0.5
0.6
0.7
0.8
survival ratio
time
1.00
0.75
0.50
0.25
0.00
0
50
100
150
200

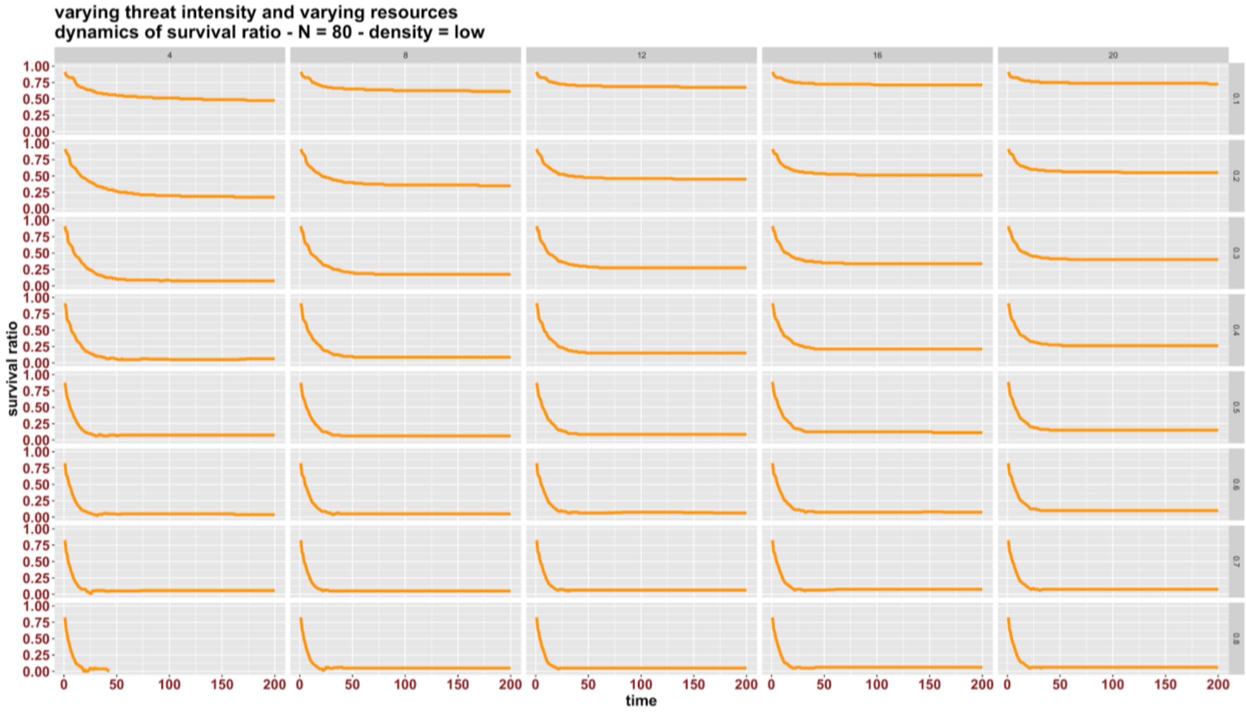
varying threat intensity and varying resources
dynamics of survival ratio - N = 80 - density = low
4
8
12
16
20
0.1
0.2
0.3
0.4
0.5
0.6
0.7
0.8
survival ratio
time
1.00
0.75
0.50
0.25
0.00
0
50
100
150
200

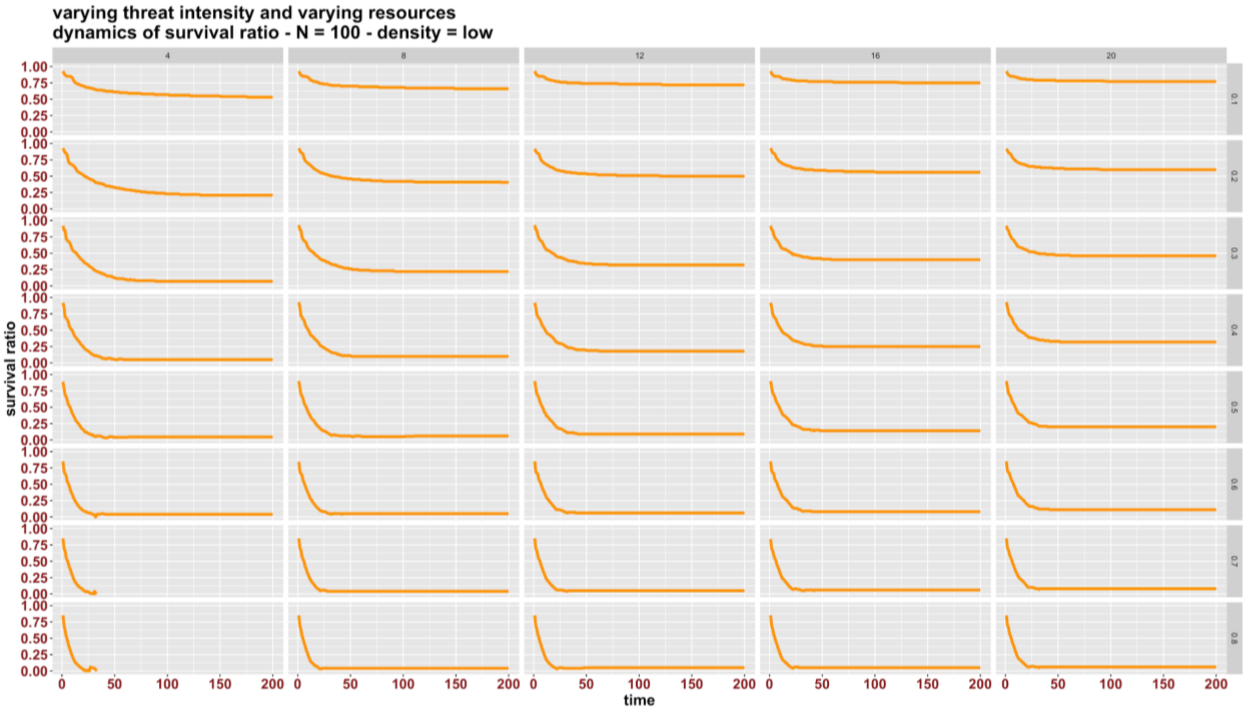
varying threat intensity and varying resources
dynamics of survival ratio - N = 100 - density = low
4
8
12
16
20
0.1
0.2
0.3
0.4
0.5
0.6
0.7
0.8
survival ratio
1.00
0.75
0.50
0.25
0.00
time
0
50
100
150
200

References

Cramer, J. S. (2004). "The early origins of the logit model". *Studies in History and Philosophy of Science Part C: Studies in History and Philosophy of Biological and Biomedical Sciences*. **35** (4): 613–626. doi:10.1016/j.shpsc.2004.09.003

Grimm, V., Berger, U., DeAngelis, D.L., Polhill, J.G., Giske, J., Railsback S.F. (2010). The ODD protocol: A review and first update, Ecological Modelling, 221(23): 2760-2768, doi.org/10.1016/j.ecolmodel.2010.08.019.

Zurell, D., Berger, U., Cabral, J. S., Jeltsch, F., Meynard, C. N., Münkemüller, T., ... & Grimm, V. (2010). The virtual ecologist approach: simulating data and observers. *Oikos*, *119*(4): 622-635.
